# Supplementary material for: Transcription factor family‐specific DNA shape readout revealed by quantitative specificity models
Source: Mol Syst Biol. 2017 Feb 6;13(2):910. doi: 10.15252/msb.20167238 (PMC5327724; doi:10.15252/msb.20167238)
Supplement: Supplementary file 1 — Appendix [file MSB-13-910-s001.pdf]

APPENDIX

Transcription factor family-specific DNA shape readout revealed by quantitative specificity models

Lin Yang[1,†], Yaron Orenstein[2,†,‡], Arttu Jolma[3], Yimeng Yin[3], Jussi Taipale[3], Ron Shamir[2,*] & Remo Rohs[1,**]

[1] Molecular and Computational Biology Program, Departments of Biological Sciences, Chemistry, Physics, and Computer Science, University of Southern California, Los Angeles, CA 90089, USA
[2] Blavatnik School of Computer Science, Tel Aviv University, Tel Aviv 69978, Israel
[3] Division of Functional Genomics and Systems Biology, Department of Medical Biochemistry and Biophysics, Karolinska Institutet, Stockholm SE 141 83, Sweden

[†] These authors contributed equally to this work.
[‡] Present address: Computer Science and Artificial Intelligence Laboratory, Massachusetts Institute of Technology, Cambridge, MA 02139, USA
* Corresponding author. Tel: +972-3-640-5383; E-mail: rshamir@tau.ac.il
** Corresponding author. Tel: +1-213-740-0552; E-mail: rohs@usc.edu

TABLE OF CONTENTS

Appendix Figures

Appendix Figure S1. *Processed data compared to gcPBM binding score.* 2

Appendix Figure S2. *Principal component analysis (PCA) using randomly generated features.* 3

Appendix Figure S3. *Performance comparison between different models.* 4

Appendix Figure S4. *Positional DNA shape importance revealed by feature selection for different TF families.* 6

Appendix Figure S5. *Feature-specific positional DNA shape importance revealed by feature selection for different TF families.* 11

Appendix Figure S6. *Structure analysis of a nuclear receptor protein in complex with DNA revealed distinct characteristics of DNA shape features in the central region which is also highlighted in the heat map analysis.* 15

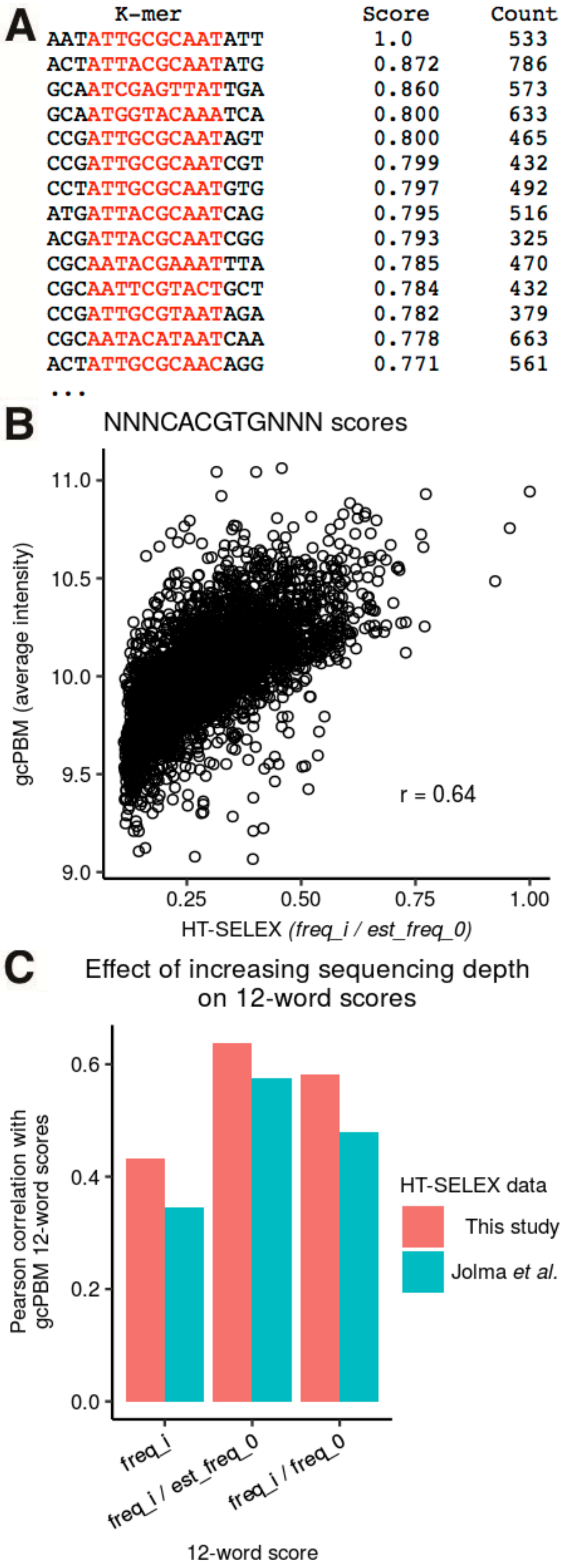

Appendix Figure S1. Processed data compared to gcPBM binding score.

A. HT-SELEX *M*-word scores. For each *M*-word with the core consensus in the center, we produced an *M*-word score by the ratio of the frequency in cycle 3 (or later cycles) over estimated frequency in the initial cycle. The core consensus is highlighted in red.

B. The gcPBM and HT-SELEX 12-word scores for the Max homodimer. The gcPBM scores were the average of log-normalized binding intensities. HT-SELEX scores were the log of the ratio of the frequency in cycle 3 over the estimated frequency of the initial cycle.

C. Comparison of *M*-word scores applied to previously published data (Jolma et al, 2013) and new augmented data (this study) in correlation to gcPBM 12-word scores for the Max homodimer. *Freq_i* is the frequency at cycle *i*; *est_freq_0* is the estimated frequency at the initial cycle based on a fifth-order Markov model

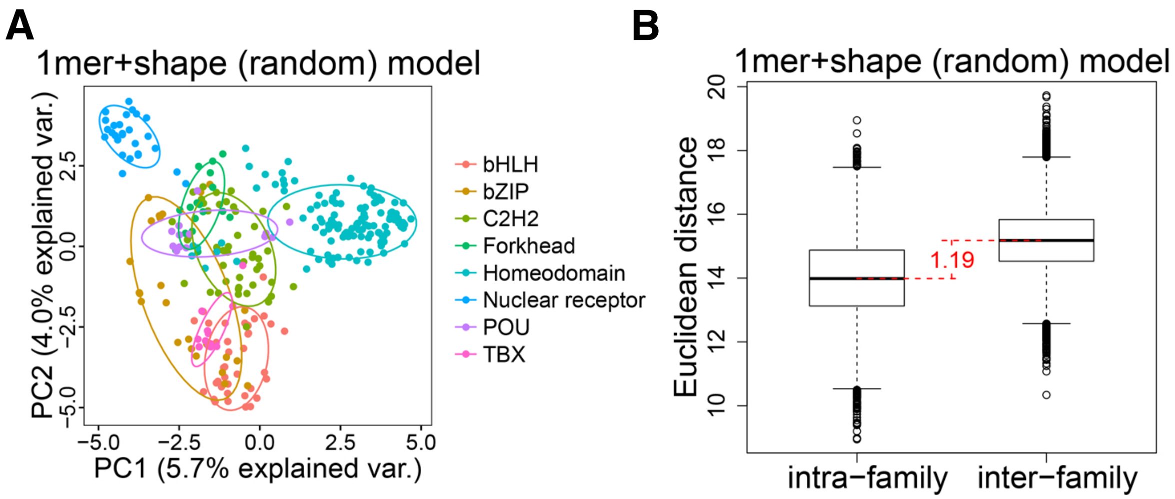

Appendix Figure S2. Principal component analysis (PCA) using randomly generated features.

A. PCA using 1-mer and randomly generated shape features. Each dot represents a transcription factor (TF). Dots of the same color belong to the same TF family. An ellipse was drawn for each TF family. The ellipse is a contour of a fitted two-variate normal distribution that encloses 0.68 (R package default) probability.

B. Boxplots of inter- and intra-family TF distances derived from A. Difference between medians of inter- and intra-family distances is 1.19 (red).

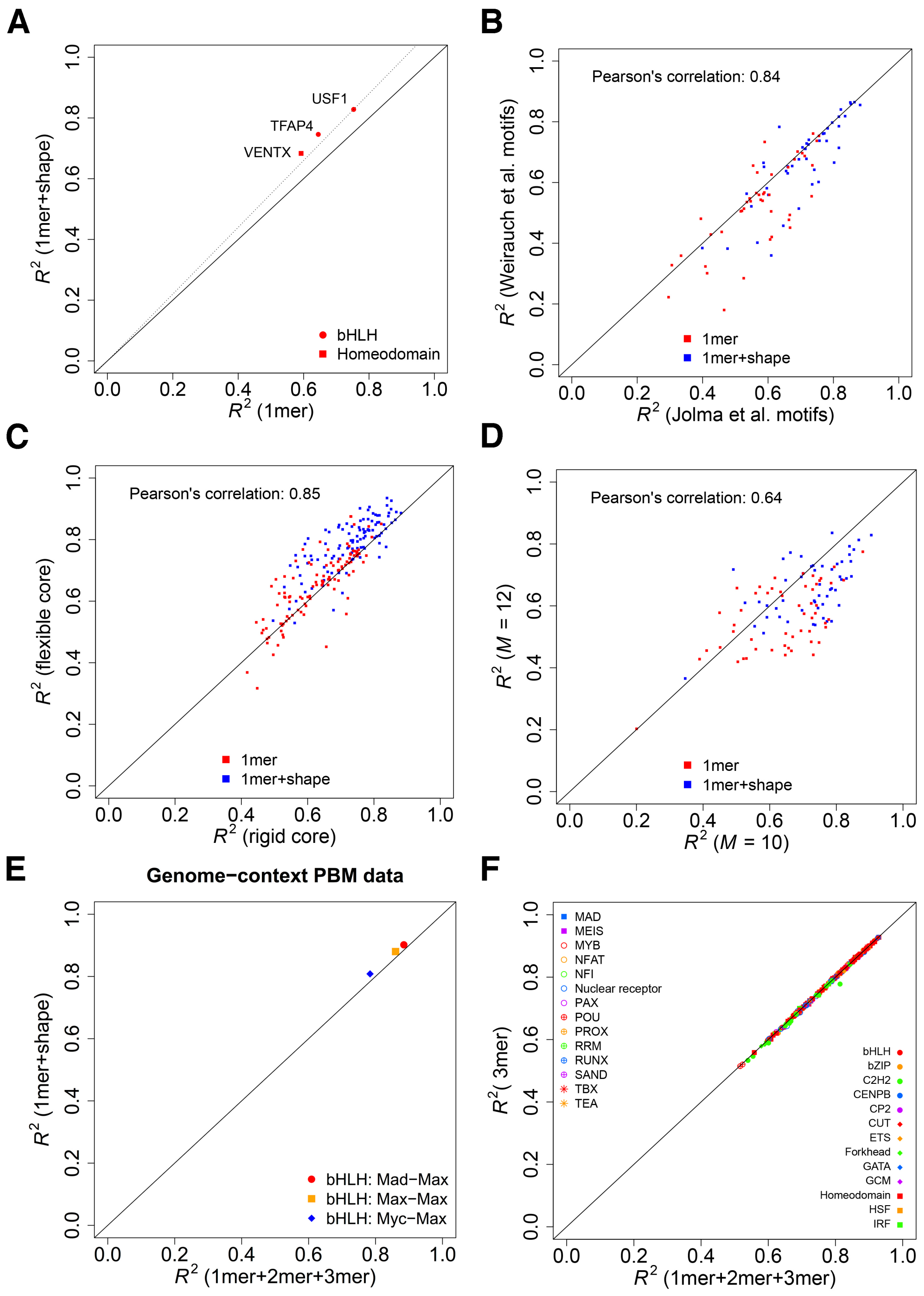
A
USF1
TFAP4
VENTX
bHLH
Homeodomain
R^2 (1mer+shape)
R^2 (1mer)
B
Pearson's correlation: 0.84
1mer
1mer+shape
R^2 (Weirauch et al. motifs)
R^2 (Jolma et al. motifs)
C
Pearson's correlation: 0.85
1mer
1mer+shape
R^2 (flexible core)
R^2 (rigid core)
D
Pearson's correlation: 0.64
1mer
1mer+shape
R^2 (M = 12)
R^2 (M = 10)
E
Genome−context PBM data
bHLH: Mad−Max
bHLH: Max−Max
bHLH: Myc−Max
R^2 (1mer+shape)
R^2 (1mer+2mer+3mer)
F
MAD
MEIS
MYB
NFAT
NFI
Nuclear receptor
PAX
POU
PROX
RRM
RUNX
SAND
TBX
TEA
bHLH
bZIP
C2H2
CENPB
CP2
CUT
ETS
Forkhead
GATA
GCM
Homeodomain
HSF
IRF
R^2(3mer)
R^2 (1mer+2mer+3mer)

Appendix Figure S3. Performance comparison between different models.

A. Performance comparison between 1mer and 1mer+shape models for replicate HT-SELEX experiments.

B. Comparison of model performance for preprocessing that used Jolma et al. (Jolma et al, 2013) seeds and Weirauch & Hughes (Weirauch & Hughes, 2011) seeds.

C. Comparison of model performance for preprocessing that allowed different numbers of mismatches at the core motif positions.

D. Comparison of model performance for preprocessing using different lengths of the flanking regions.

E. Performance comparison between 1mer+2mer+3mer and 1mer+shape models for gcPBM data.

F. Performance comparison between 1mer+2mer+3mer and 3mer models for HT-SELEX data.

Each dot represents one dataset. Coordinates of the dot are determined by the performance, measured in R^2 based on 10-fold cross-validation, of the corresponding models indicated in parentheses. Shape and color of the dots indicate the TF family. Dashed line in A has a slope of 1.1, indicating 10% performance increase.

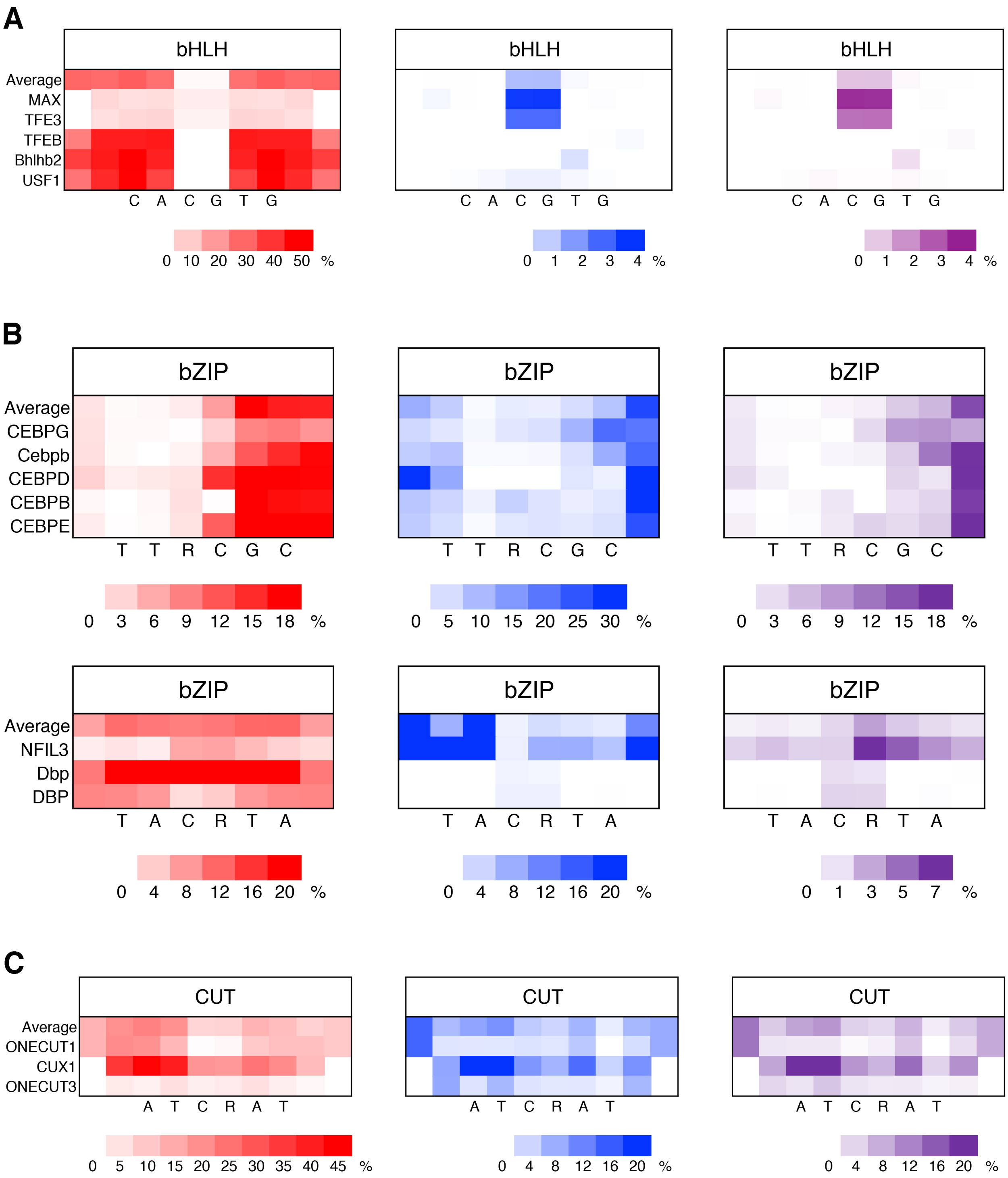
A
bHLH
Average
MAX
TFE3
TFEB
Bhlhb2
USF1
C A C G T G
0 10 20 30 40 50 %
bHLH
C A C G T G
0 1 2 3 4 %
bHLH
C A C G T G
0 1 2 3 4 %
B
bZIP
Average
CEBPG
Cebpb
CEBPD
CEBPB
CEBPE
T T R C G C
0 3 6 9 12 15 18 %
bZIP
T T R C G C
0 5 10 15 20 25 30 %
bZIP
T T R C G C
0 3 6 9 12 15 18 %
bZIP
Average
NFIL3
Dbp
DBP
T A C R T A
0 4 8 12 16 20 %
bZIP
T A C R T A
0 4 8 12 16 20 %
bZIP
T A C R T A
0 1 3 5 7 %
C
CUT
Average
ONECUT1
CUX1
ONECUT3
A T C R A T
0 5 10 15 20 25 30 35 40 45 %
CUT
A T C R A T
0 4 8 12 16 20 %
CUT
A T C R A T
0 4 8 12 16 20 %

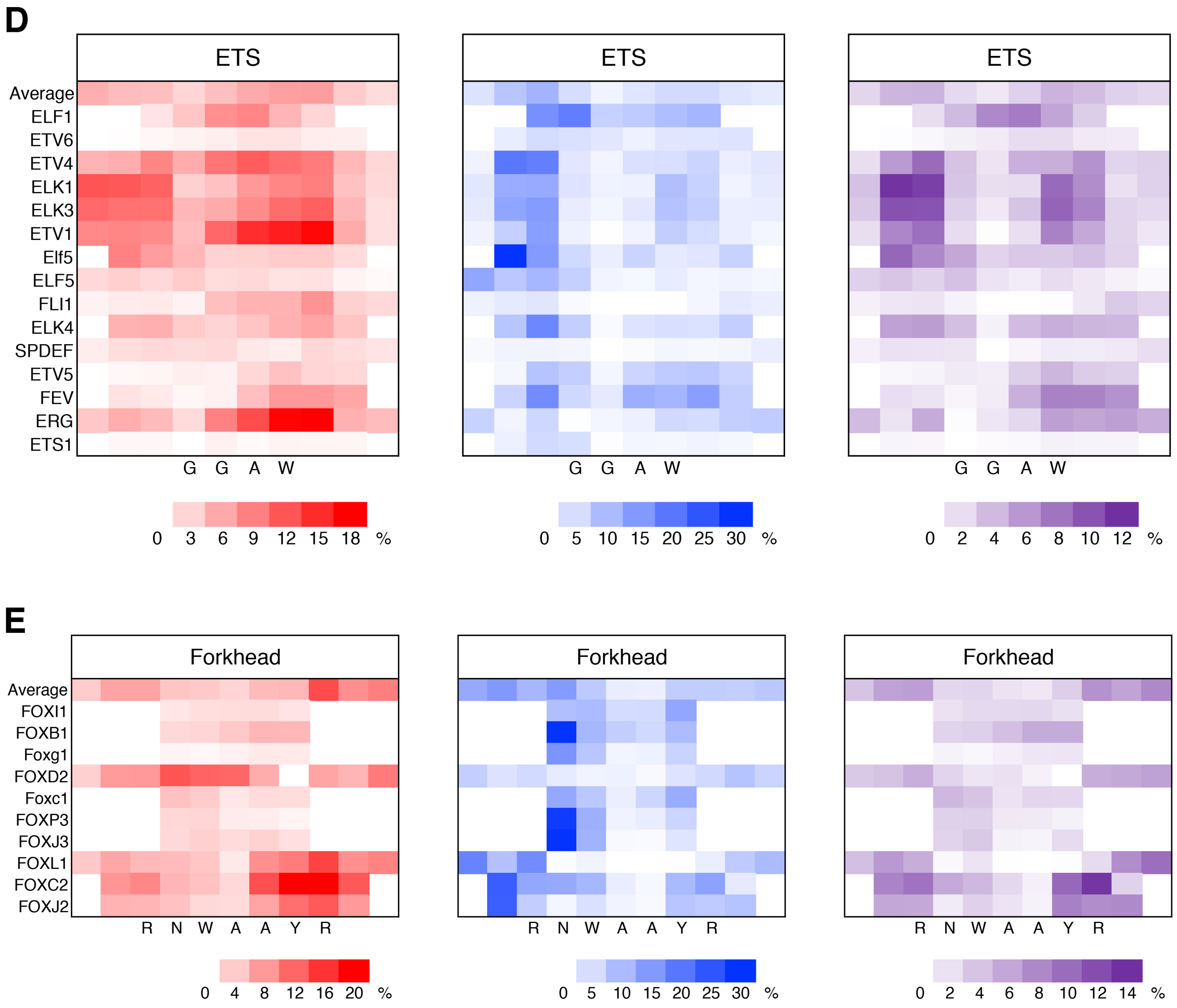
D
ETS
ETS
ETS
Average
ELF1
ETV6
ETV4
ELK1
ELK3
ETV1
Elf5
ELF5
FLI1
ELK4
SPDEF
ETV5
FEV
ERG
ETS1
G G A W
G G A W
G G A W
0 3 6 9 12 15 18 %
0 5 10 15 20 25 30 %
0 2 4 6 8 10 12 %
E
Forkhead
Forkhead
Forkhead
Average
FOXI1
FOXB1
Foxg1
FOXD2
Foxc1
FOXP3
FOXJ3
FOXL1
FOXC2
FOXJ2
R N W A A Y R
R N W A A Y R
R N W A A Y R
0 4 8 12 16 20 %
0 5 10 15 20 25 30 %
0 2 4 6 8 10 12 14 %

F

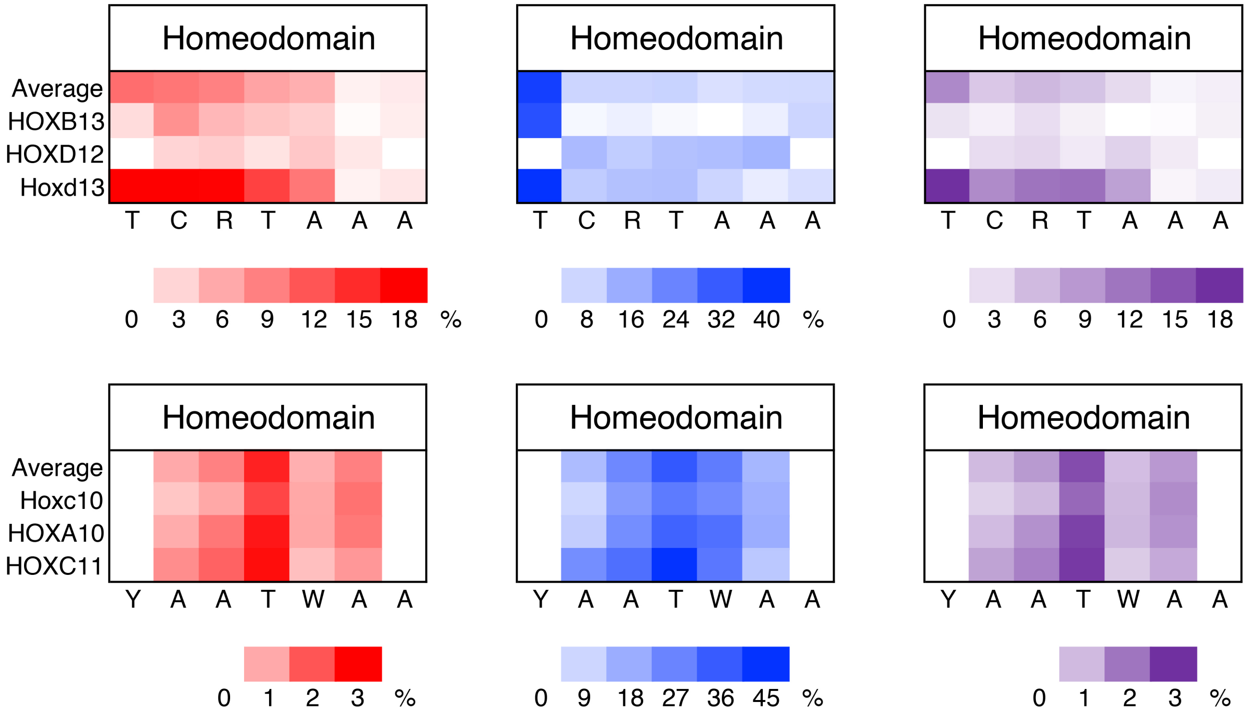
Homeodomain
Homeodomain
Homeodomain
Average
HOXB13
HOXD12
Hoxd13
T C R T A A A
T C R T A A A
T C R T A A A
0 3 6 9 12 15 18 %
0 8 16 24 32 40 %
0 3 6 9 12 15 18
Homeodomain
Homeodomain
Homeodomain
Average
Hoxc10
HOXA10
HOXC11
Y A A T W A A
Y A A T W A A
Y A A T W A A
0 1 2 3 %
0 9 18 27 36 45 %
0 1 2 3 %

G

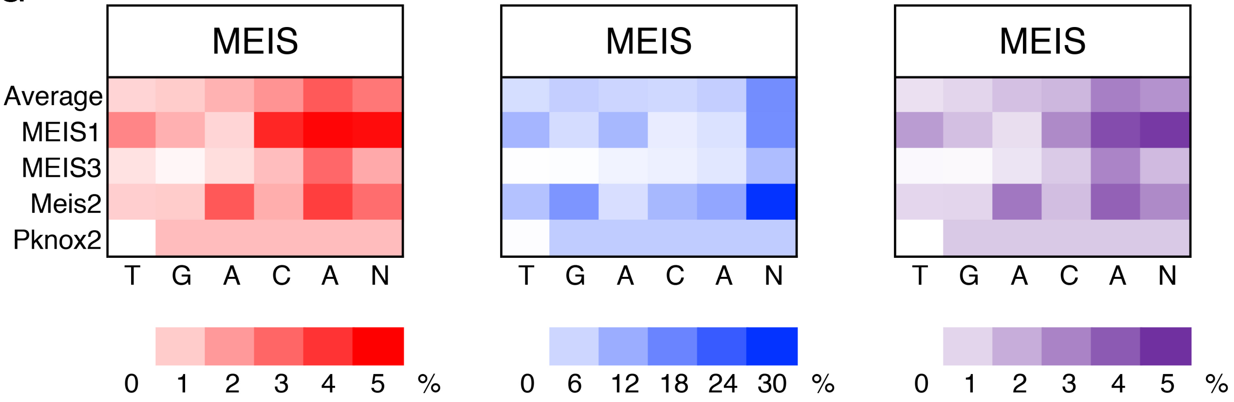
MEIS
MEIS
MEIS
Average
MEIS1
MEIS3
Meis2
Pknox2
T G A C A N
T G A C A N
T G A C A N
0 1 2 3 4 5 %
0 6 12 18 24 30 %
0 1 2 3 4 5 %

H

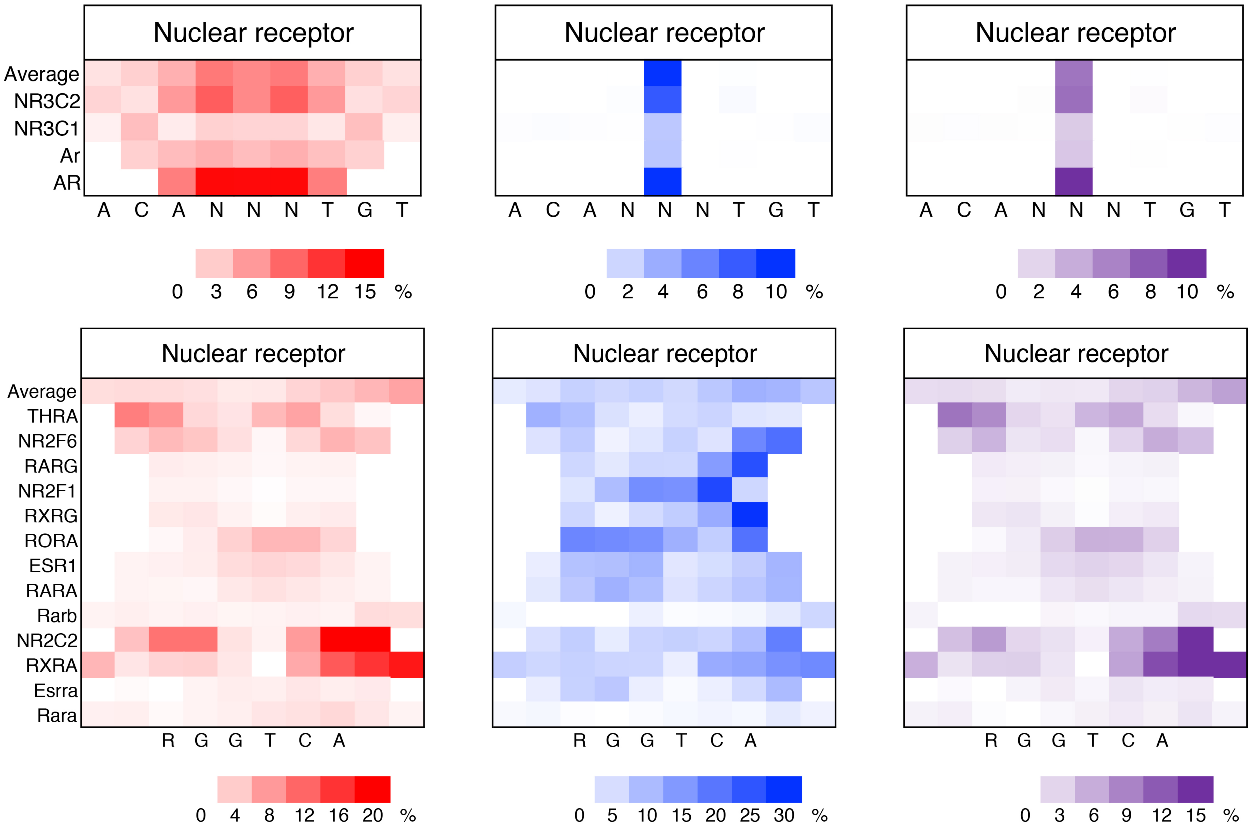
Nuclear receptor
Average
NR3C2
NR3C1
Ar
AR
A C A N N N T G T
0 3 6 9 12 15 %
0 2 4 6 8 10 %
Nuclear receptor
Average
THRA
NR2F6
RARG
NR2F1
RXRG
RORA
ESR1
RARA
Rarb
NR2C2
RXRA
Esrra
Rara
R G G T C A
0 4 8 12 16 20 %
0 5 10 15 20 25 30 %
0 3 6 9 12 15 %

I

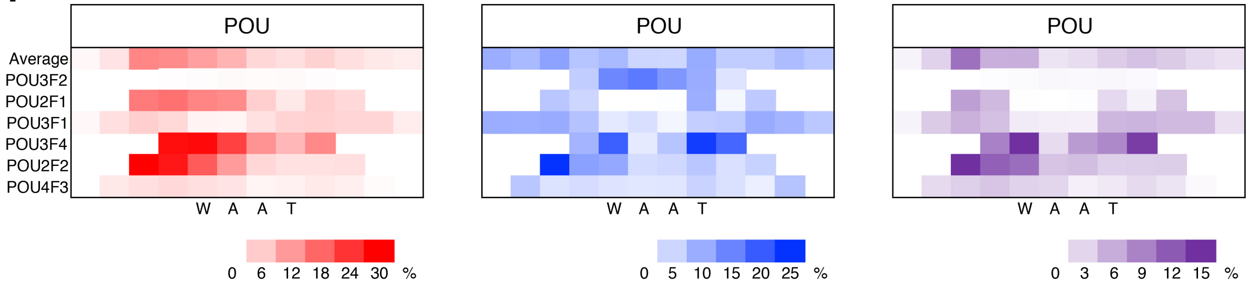
POU
Average
POU3F2
POU2F1
POU3F1
POU3F4
POU2F2
POU4F3
W A A T
0 6 12 18 24 30 %
0 5 10 15 20 25 %
0 3 6 9 12 15 %

J

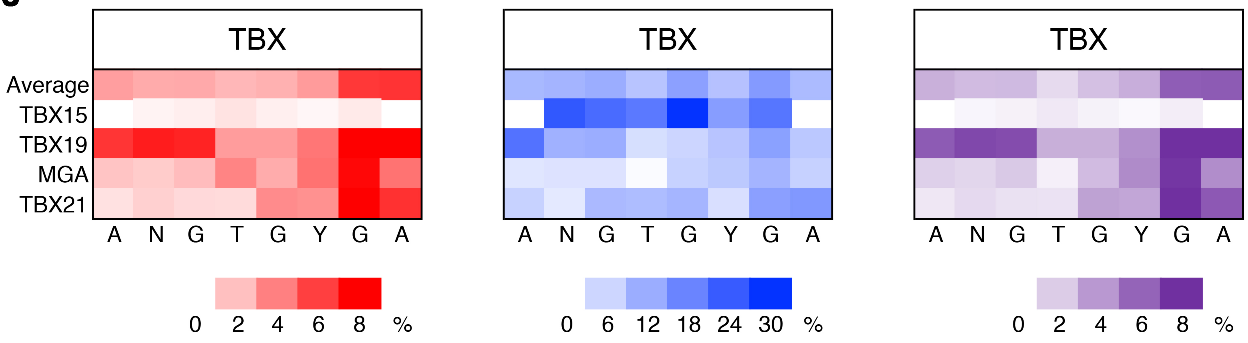

Appendix Figure S4. Positional DNA shape importance revealed by feature selection for different TF families.

Heat maps derived from feature selection, where DNA shape features were added position by position to a baseline 1mer model (red heat maps), DNA shape features were removed position by position from a baseline shape-only model (blue heat maps), and the red and blue heat maps were combined by taking the minimum of these two heat maps (resulting in the combined purple heat maps), for the following TF families.

A. bHLH (basic helix-loop-helix).
B. bZIP (basic-leucine zipper).
C. CUT.
D. ETS.
E. Forkhead.
F. Homeodomain.
G. MEIS.
H. Nuclear receptor.
I. POU.
J. TBX.

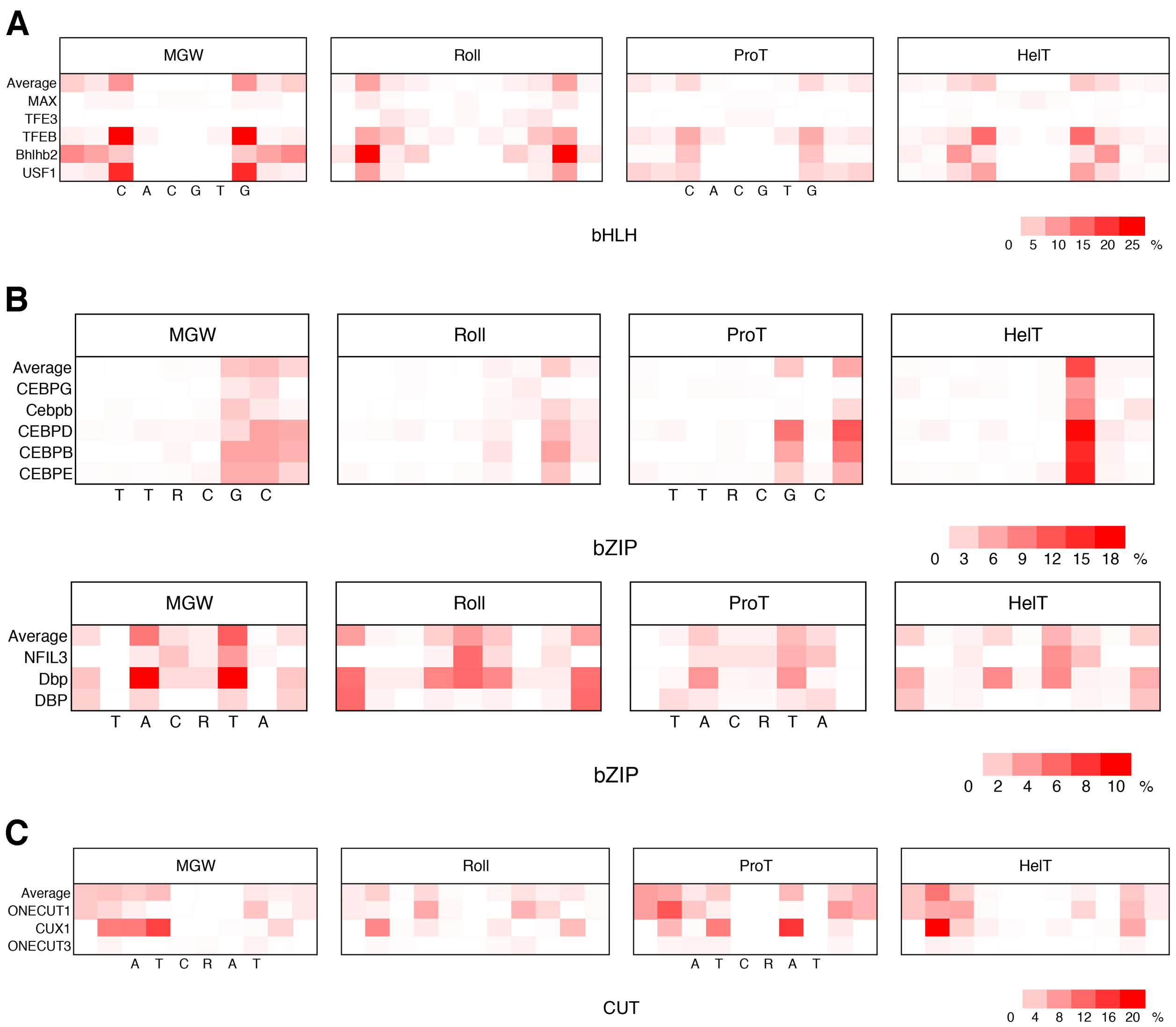
A
MGW
Roll
ProT
HelT
Average
MAX
TFE3
TFEB
Bhlhb2
USF1
C A C G T G
bHLH
0 5 10 15 20 25 %
B
MGW
Roll
ProT
HelT
Average
CEBPG
Cebpb
CEBPD
CEBPB
CEBPE
T T R C G C
bZIP
0 3 6 9 12 15 18 %
Average
NFIL3
Dbp
DBP
T A C R T A
bZIP
0 2 4 6 8 10 %
C
MGW
Roll
ProT
HelT
Average
ONECUT1
CUX1
ONECUT3
A T C R A T
CUT
0 4 8 12 16 20 %

D

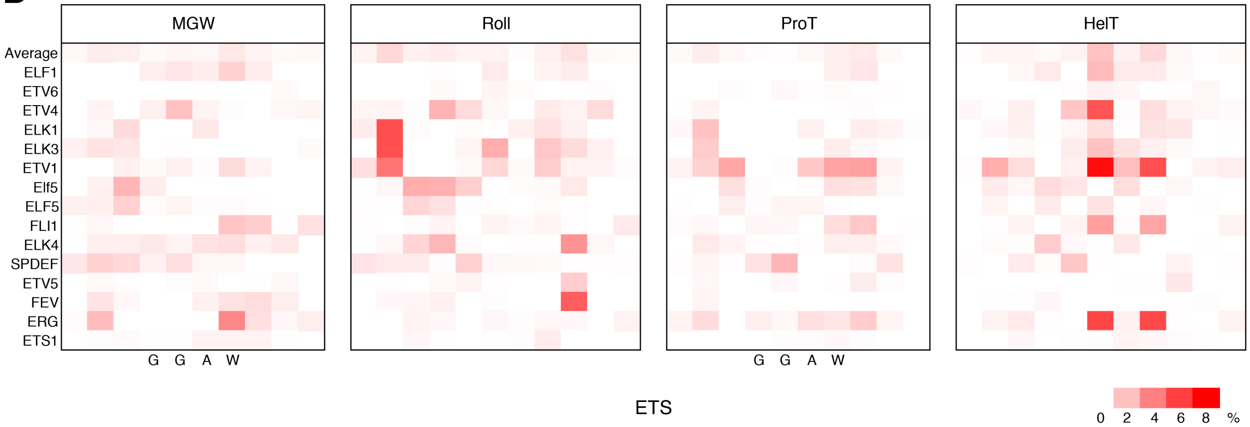

MGW
Roll
ProT
HelT
Average
ELF1
ETV6
ETV4
ELK1
ELK3
ETV1
Elf5
ELF5
FLI1
ELK4
SPDEF
ETV5
FEV
ERG
ETS1
G G A W
G G A W
ETS
0 2 4 6 8 %

E

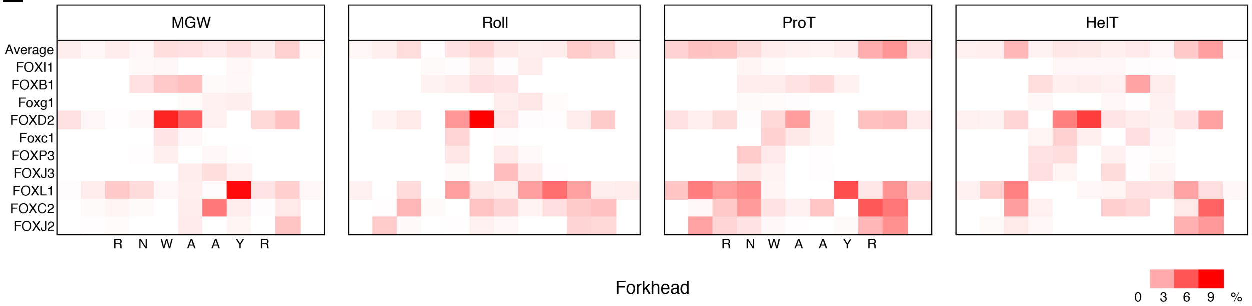

MGW
Roll
ProT
HelT
Average
FOXI1
FOXB1
Foxg1
FOXD2
Foxc1
FOXP3
FOXJ3
FOXL1
FOXC2
FOXJ2
R N W A A Y R
R N W A A Y R
Forkhead
0 3 6 9 %

F

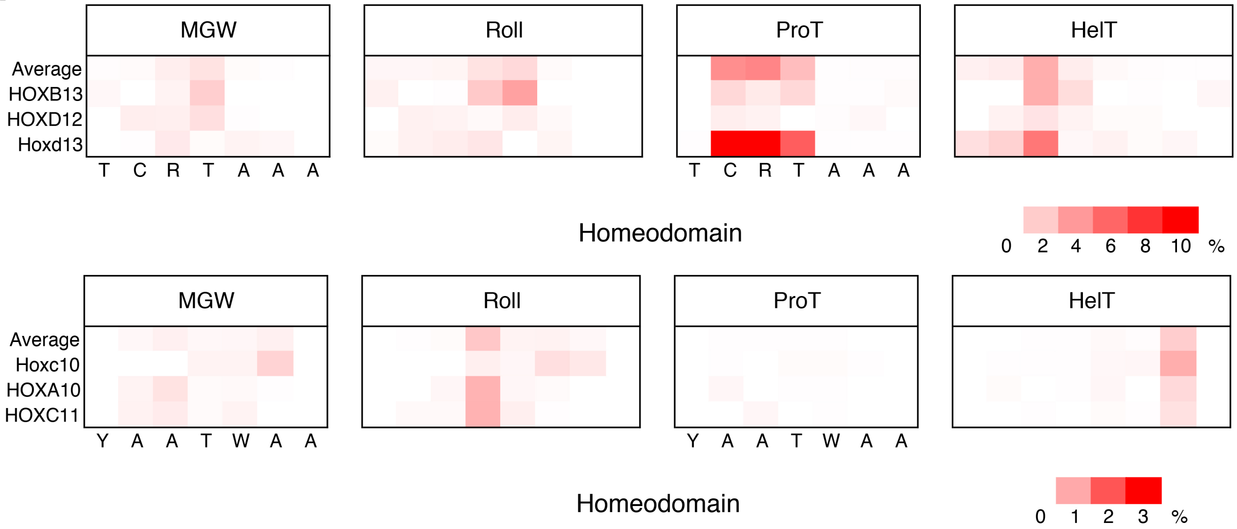

MGW
Roll
ProT
HelT
Average
HOXB13
HOXD12
Hoxd13
T C R T A A A
T C R T A A A
Homeodomain
0 2 4 6 8 10 %
MGW
Roll
ProT
HelT
Average
Hoxc10
HOXA10
HOXC11
Y A A T W A A
Y A A T W A A
Homeodomain
0 1 2 3 %

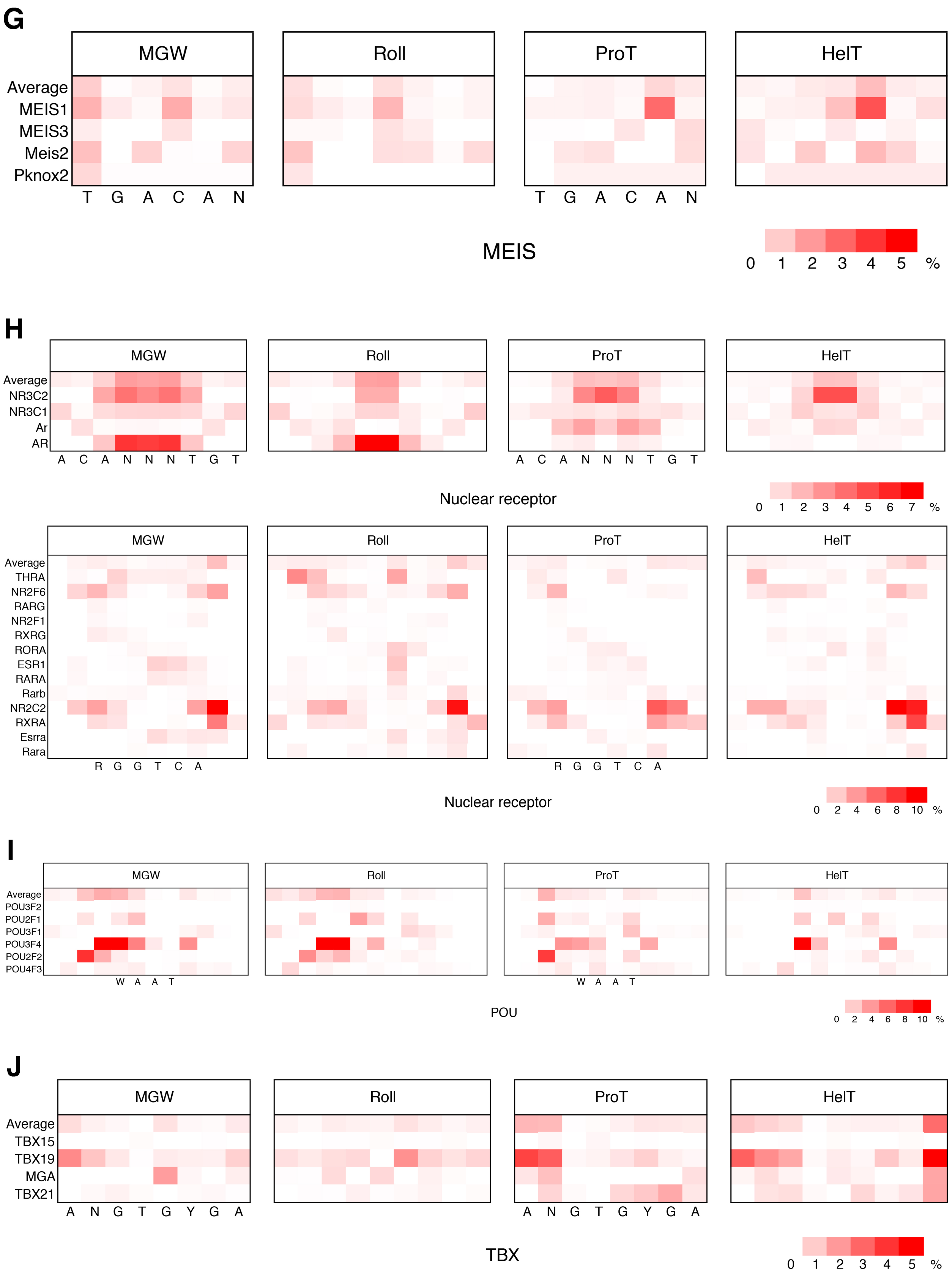
G
MGW
Roll
ProT
HelT
Average
MEIS1
MEIS3
Meis2
Pknox2
T G A C A N
T G A C A N
MEIS
0 1 2 3 4 5 %
H
MGW
Roll
ProT
HelT
Average
NR3C2
NR3C1
Ar
AR
A C A N N N T G T
A C A N N N T G T
Nuclear receptor
0 1 2 3 4 5 6 7 %
MGW
Roll
ProT
HelT
Average
THRA
NR2F6
RARG
NR2F1
RXRG
RORA
ESR1
RARA
Rarb
NR2C2
RXRA
Esrra
Rara
R G G T C A
R G G T C A
Nuclear receptor
0 2 4 6 8 10 %
I
MGW
Roll
ProT
HelT
Average
POU3F2
POU2F1
POU3F1
POU3F4
POU2F2
POU4F3
W A A T
W A A T
POU
0 2 4 6 8 10 %
J
MGW
Roll
ProT
HelT
Average
TBX15
TBX19
MGA
TBX21
A N G T G Y G A
A N G T G Y G A
TBX
0 1 2 3 4 5 %

Appendix Figure S5. Feature-specific positional DNA shape importance revealed by feature selection for different TF families.

Heat maps derived from feature selection, where each category of DNA shape features, i.e. MGW, Roll, ProT and HelT, was added position by position to a baseline 1mer model, for the following TF families.

A. bHLH (basic helix-loop-helix).
B. bZIP (basic-leucine zipper).
C. CUT.
D. ETS.
E. Forkhead.
F. Homeodomain.
G. MEIS.
H. Nuclear receptor.
I. POU.
J. TBX.

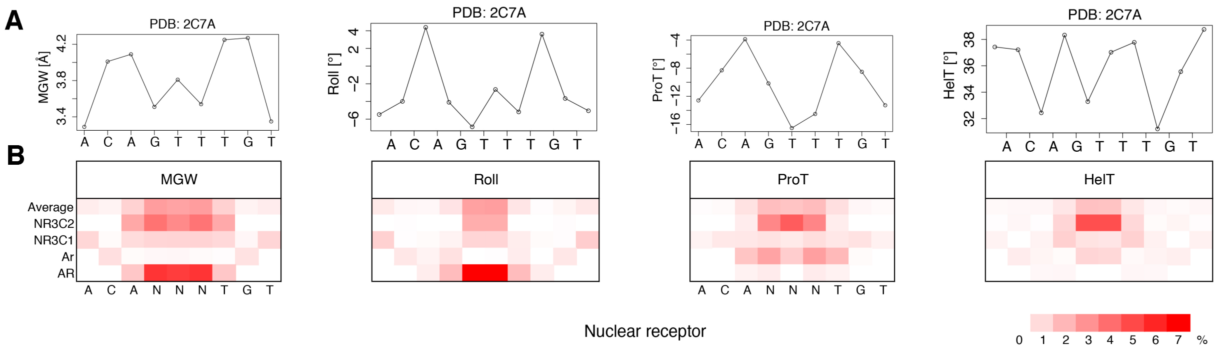

Appendix Figure S6. Structure analysis of a nuclear receptor protein in complex with DNA revealed distinct characteristics of DNA shape features in the central region which is also highlighted in the heat map analysis.

A. CURVES (Lavery & Sklenar, 1989) derived plots for MGW, Roll, ProT, and HelT of the bound DNA in the progesterone receptor-DNA complex (PDB ID 2C7A).

B. Heat maps from Appendix Figure S5H aligned to the plots in A.

References:

Jolma A, Yan J, Whitington T, Toivonen J, Nitta KR, Rastas P, Morgunova E, Enge M, Taipale M, Wei G, Palin K, Vaquerizas JM, Vincentelli R, Luscombe NM, Hughes TR, Lemaire P, Ukkonen E, Kivioja T, Taipale J (2013). DNA-binding specificities of human transcription factors. Cell 152: 327-339

Lavery R, Sklenar H (1989). Defining the structure of irregular nucleic acids: conventions and principles. Journal of Biomolecular Structure and Dynamics 6: 655-667

Weirauch MT, Hughes T (2011). A catalogue of eukaryotic transcription factor types, their evolutionary origin, and species distribution. In A Handbook of Transcription Factors, pp 25-73. Springer Netherlands
