# Supplementary figures and images for: Transcription factor family‐specific DNA shape readout revealed by quantitative specificity models (part 2 of 4)
Source: Mol Syst Biol. 2017 Feb 6;13(2):910. doi: 10.15252/msb.20167238 (PMC5327724; doi:10.15252/msb.20167238)

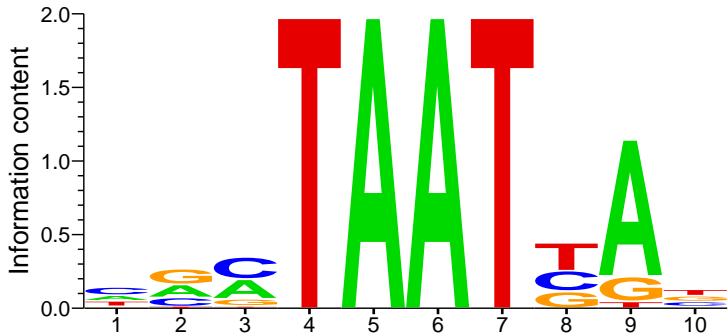

Supplement: Supplementary file 6 — Dataset EV1 [file MSB-13-910-s006.zip › Yang_Orenstein_DatasetEV1/homeodomain_EVX1_TGGGAC20NAGT_TAAT_10_3.pwm.pdf]

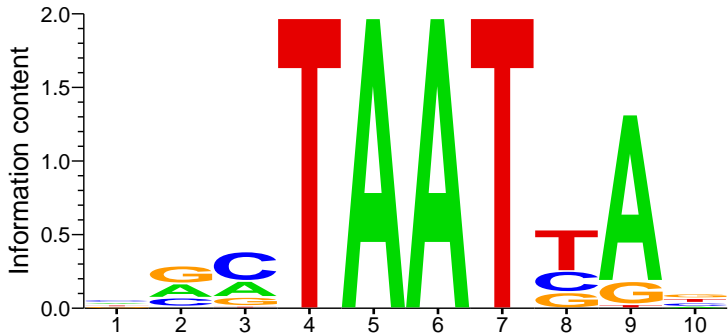

Supplement: Supplementary file 6 — Dataset EV1 [file MSB-13-910-s006.zip › Yang_Orenstein_DatasetEV1/homeodomain_EVX2_TCAAGG20NGAA_TAAT_10_4.pwm.pdf]

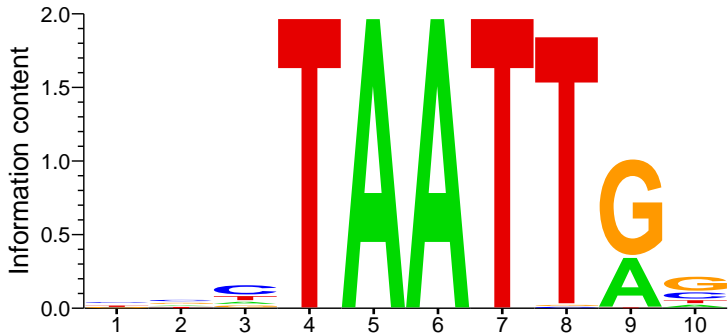

Supplement: Supplementary file 6 — Dataset EV1 [file MSB-13-910-s006.zip › Yang_Orenstein_DatasetEV1/homeodomain_GBX1_TAGTCC20NCG_TAAT_10_3.pwm.pdf]

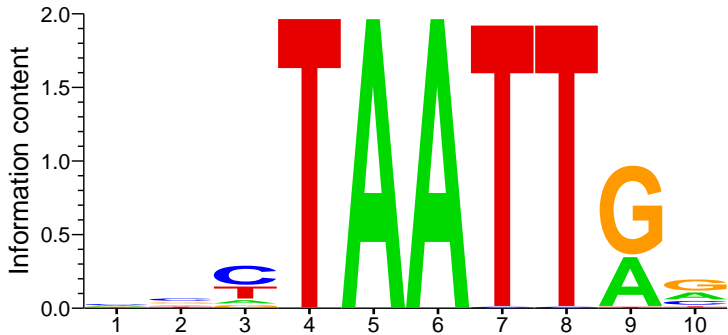

Supplement: Supplementary file 6 — Dataset EV1 [file MSB-13-910-s006.zip › Yang_Orenstein_DatasetEV1/homeodomain_Gbx1_TCGGTT20NCG_TAAT_10_4.pwm.pdf]

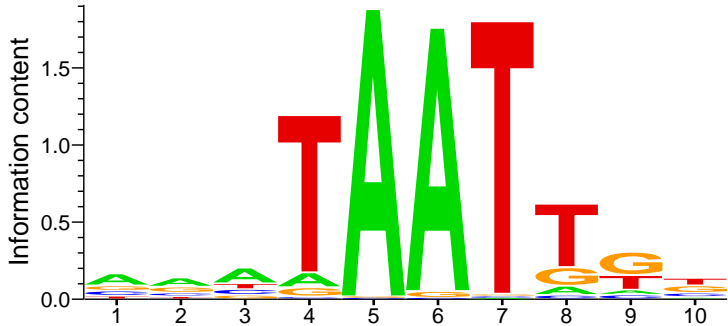

Supplement: Supplementary file 6 — Dataset EV1 [file MSB-13-910-s006.zip › Yang_Orenstein_DatasetEV1/homeodomain_Gbx2_TACACC20NCAC_TAAT_10_3.pwm.pdf]

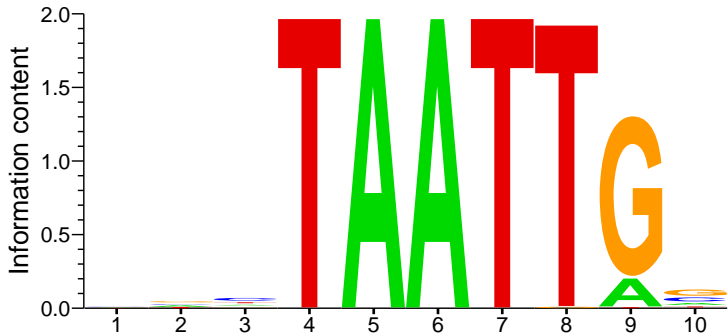

Supplement: Supplementary file 6 — Dataset EV1 [file MSB-13-910-s006.zip › Yang_Orenstein_DatasetEV1/homeodomain_GBX2_TCCCGA20NGCC_TAAT_10_4.pwm.pdf]

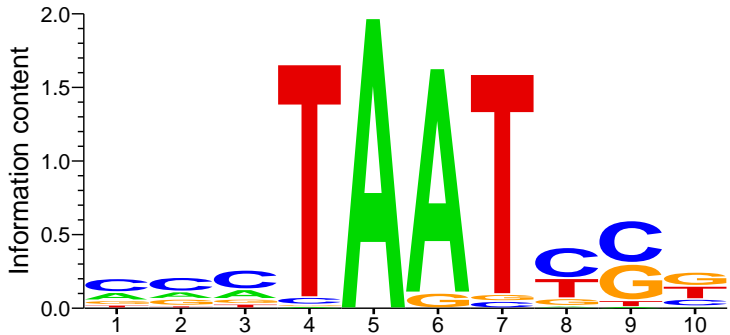

Supplement: Supplementary file 6 — Dataset EV1 [file MSB-13-910-s006.zip › Yang_Orenstein_DatasetEV1/homeodomain_GSC_TGGGAC20NGA_TAAT_10_3.pwm.pdf]

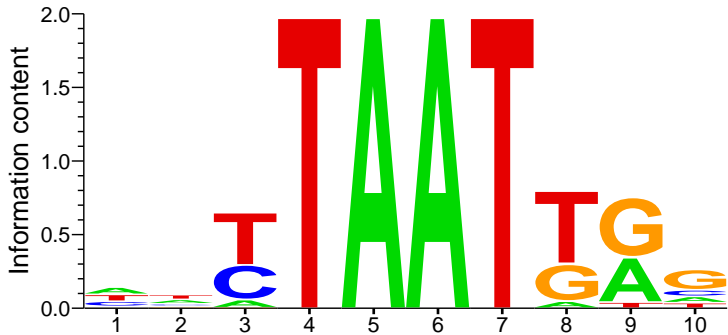

Supplement: Supplementary file 6 — Dataset EV1 [file MSB-13-910-s006.zip › Yang_Orenstein_DatasetEV1/homeodomain_GSX2_TCCAAC20NCG_TAAT_10_4.pwm.pdf]

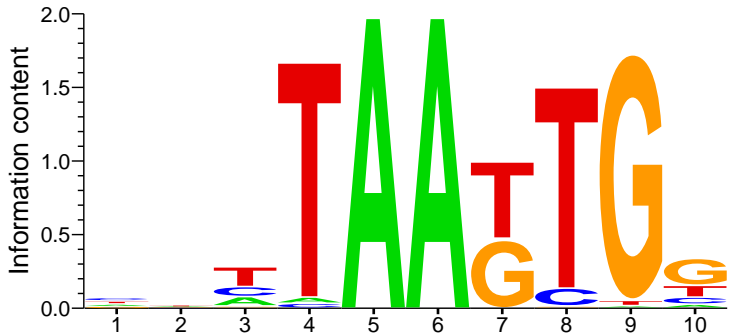

Supplement: Supplementary file 6 — Dataset EV1 [file MSB-13-910-s006.zip › Yang_Orenstein_DatasetEV1/homeodomain_HMX2_TAGTGG20NCG_TAAK_10_3.pwm.pdf]

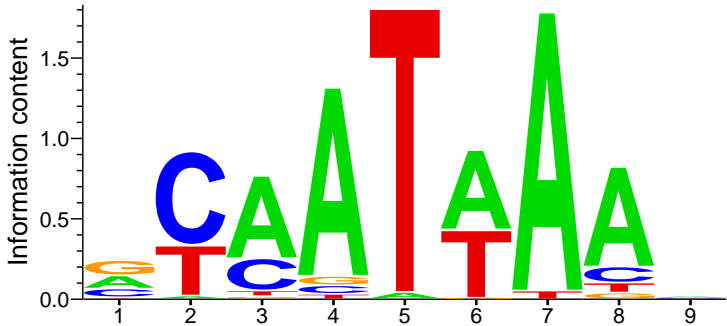

Supplement: Supplementary file 6 — Dataset EV1 [file MSB-13-910-s006.zip › Yang_Orenstein_DatasetEV1/homeodomain_HOXA10_TAGGAT30NAGT_YAATWAA_9_3.pwm.pdf]

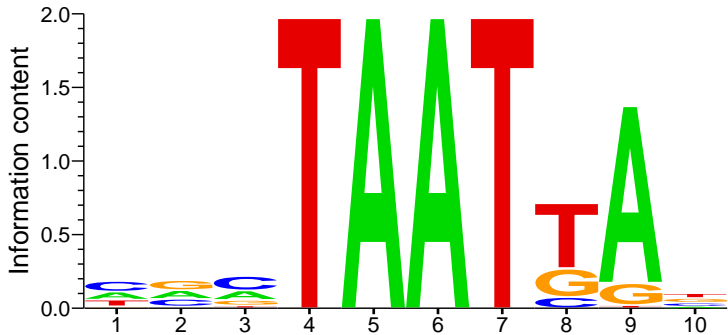

Supplement: Supplementary file 6 — Dataset EV1 [file MSB-13-910-s006.zip › Yang_Orenstein_DatasetEV1/homeodomain_Hoxa2_TACCGT20NCG_TAAT_10_4.pwm.pdf]

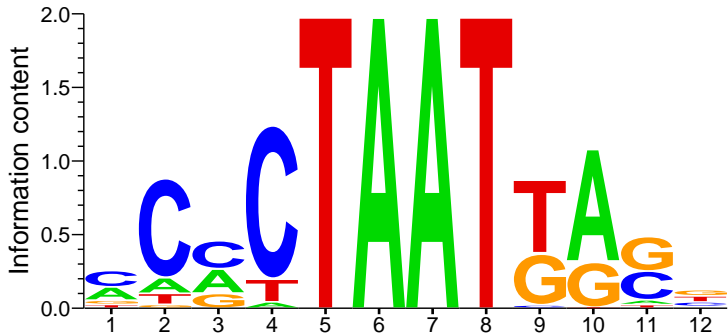

Supplement: Supplementary file 6 — Dataset EV1 [file MSB-13-910-s006.zip › Yang_Orenstein_DatasetEV1/homeodomain_HOXA2_TTATTA20NTA_TAAT_12_4.pwm.pdf]

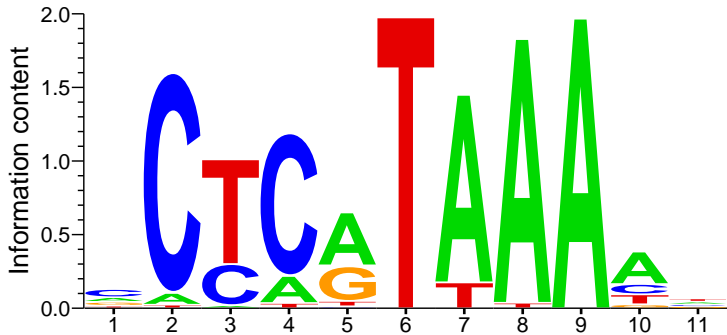

Supplement: Supplementary file 6 — Dataset EV1 [file MSB-13-910-s006.zip › Yang_Orenstein_DatasetEV1/homeodomain_HOXB13_TGCCTG20NGA_TCGTAAA_11_4.pwm.pdf]

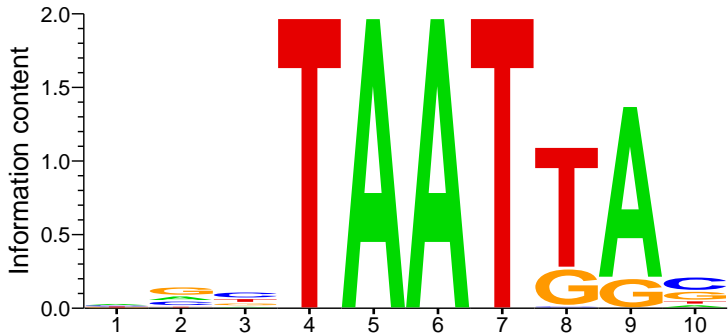

Supplement: Supplementary file 6 — Dataset EV1 [file MSB-13-910-s006.zip › Yang_Orenstein_DatasetEV1/homeodomain_HOXB2_TAACAA20NACA_TAAT_10_4.pwm.pdf]

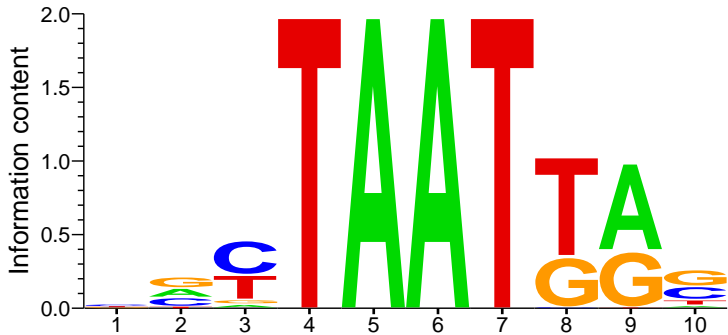

Supplement: Supplementary file 6 — Dataset EV1 [file MSB-13-910-s006.zip › Yang_Orenstein_DatasetEV1/homeodomain_HOXB3_TACATT20NTAC_TAAT_10_4.pwm.pdf]

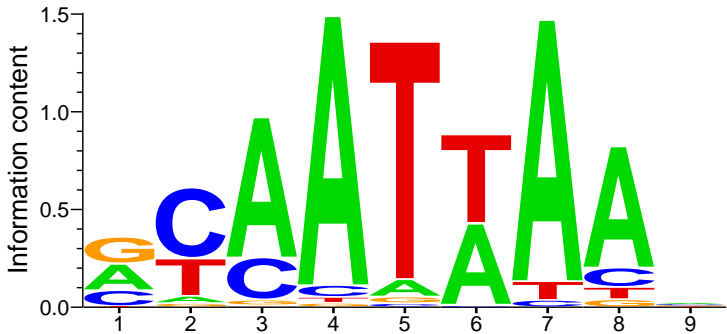

Supplement: Supplementary file 6 — Dataset EV1 [file MSB-13-910-s006.zip › Yang_Orenstein_DatasetEV1/homeodomain_Hoxc10_TATAGA20NGAG_YAATWAA_9_4.pwm.pdf]

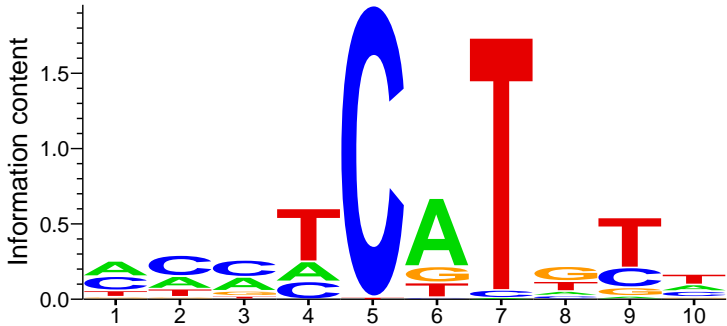

Supplement: Supplementary file 6 — Dataset EV1 [file MSB-13-910-s006.zip › Yang_Orenstein_DatasetEV1/homeodomain_Irx3_TACTGC20NCG_TCAT_10_3.pwm.pdf]

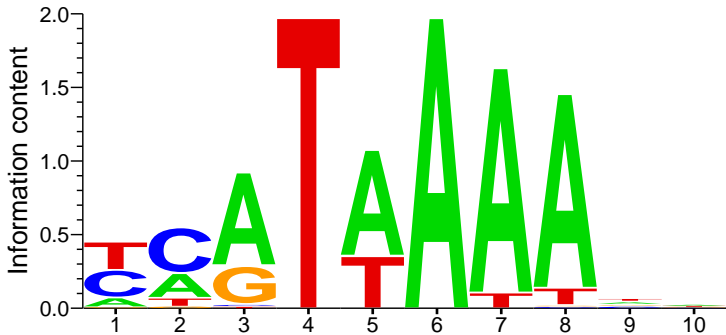

Supplement: Supplementary file 6 — Dataset EV1 [file MSB-13-910-s006.zip › Yang_Orenstein_DatasetEV1/homeodomain_HOXC10_TGGACA20NGA_GTWAAA_10_3.pwm.pdf]

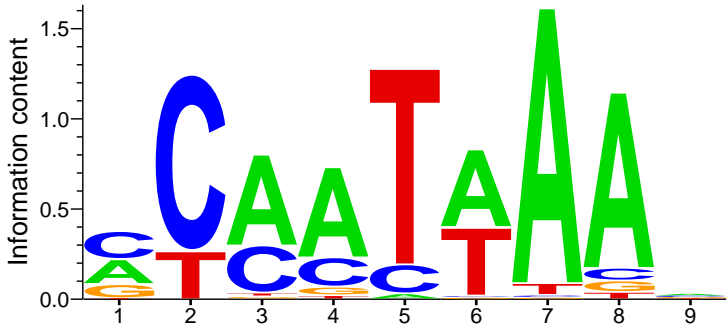

Supplement: Supplementary file 6 — Dataset EV1 [file MSB-13-910-s006.zip › Yang_Orenstein_DatasetEV1/homeodomain_HOXC11_TGCGAA20NGA_YAATWAA_9_3.pwm.pdf]

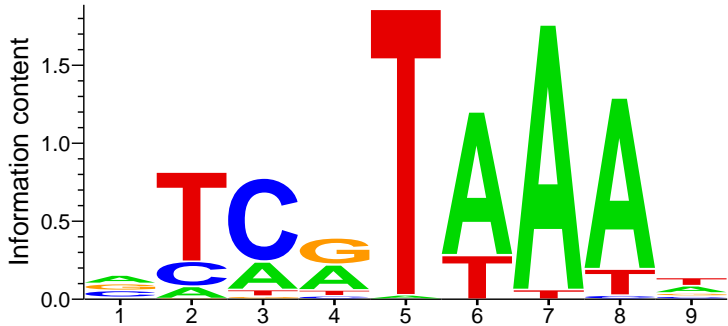

Supplement: Supplementary file 6 — Dataset EV1 [file MSB-13-910-s006.zip › Yang_Orenstein_DatasetEV1/homeodomain_HOXD12_TCAGTC20NCG_TCGTAAA_9_3.pwm.pdf]

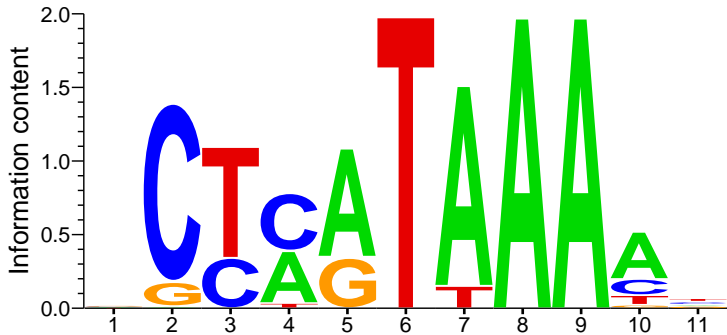

Supplement: Supplementary file 6 — Dataset EV1 [file MSB-13-910-s006.zip › Yang_Orenstein_DatasetEV1/homeodomain_Hoxd13_TGATAA20NATA_TCRTAAA_11_4.pwm.pdf]

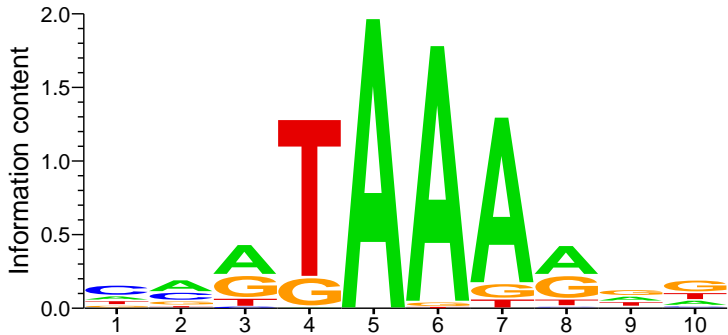

Supplement: Supplementary file 6 — Dataset EV1 [file MSB-13-910-s006.zip › Yang_Orenstein_DatasetEV1/homeodomain_Hoxd9_TCCTAA20NCG_GTAAAN_10_3.pwm.pdf]

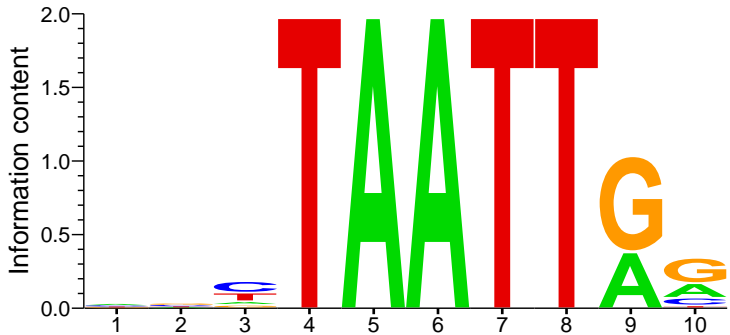

Supplement: Supplementary file 6 — Dataset EV1 [file MSB-13-910-s006.zip › Yang_Orenstein_DatasetEV1/homeodomain_ISX_TATTTA20NCG_TAAT_10_4.pwm.pdf]

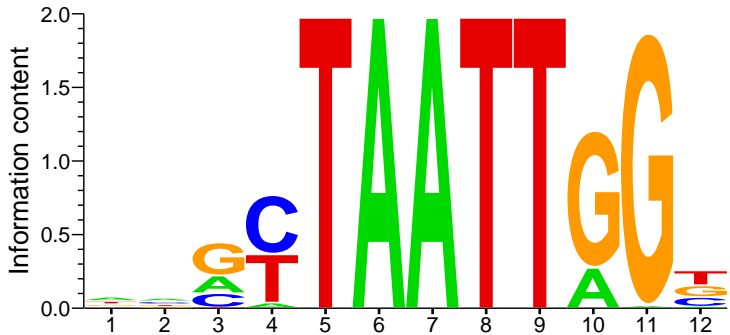

Supplement: Supplementary file 6 — Dataset EV1 [file MSB-13-910-s006.zip › Yang_Orenstein_DatasetEV1/homeodomain_LBX2_TAATTC20NTTA_TAAT_12_4.pwm.pdf]

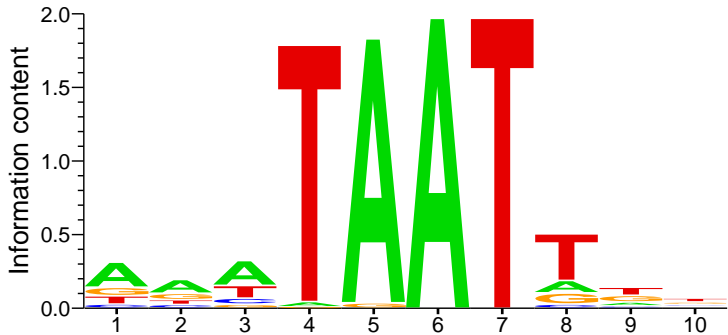

Supplement: Supplementary file 6 — Dataset EV1 [file MSB-13-910-s006.zip › Yang_Orenstein_DatasetEV1/homeodomain_LHX2_TCCAGT30NGAC_TAAT_10_3.pwm.pdf]

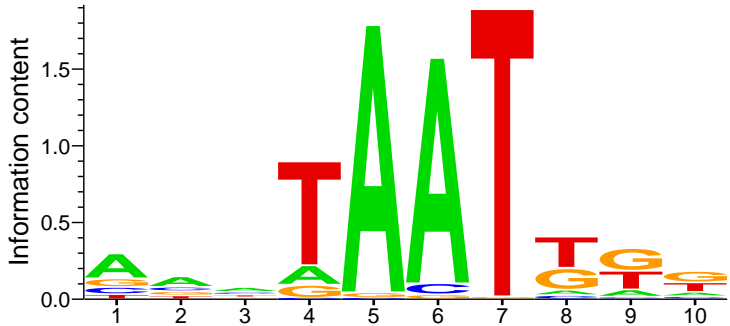

Supplement: Supplementary file 6 — Dataset EV1 [file MSB-13-910-s006.zip › Yang_Orenstein_DatasetEV1/homeodomain_Lhx4_TAAATG20NTAA_TAAT_10_3.pwm.pdf]

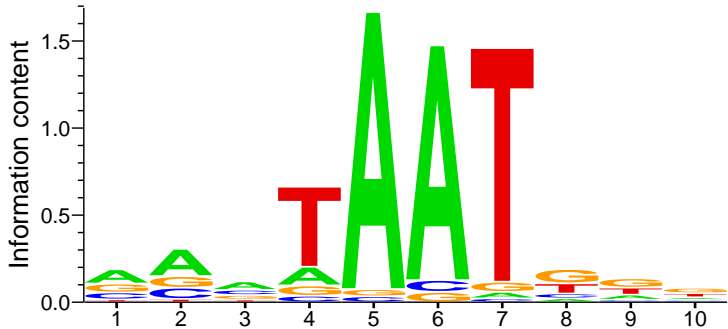

Supplement: Supplementary file 6 — Dataset EV1 [file MSB-13-910-s006.zip › Yang_Orenstein_DatasetEV1/homeodomain_LHX6_TCGGGG20NGGT_TAAT_10_3.pwm.pdf]

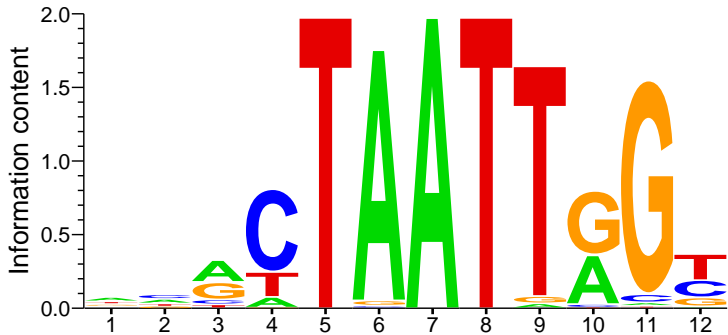

Supplement: Supplementary file 6 — Dataset EV1 [file MSB-13-910-s006.zip › Yang_Orenstein_DatasetEV1/homeodomain_LHX9_TATTGT20NGTG_TAAT_12_3.pwm.pdf]

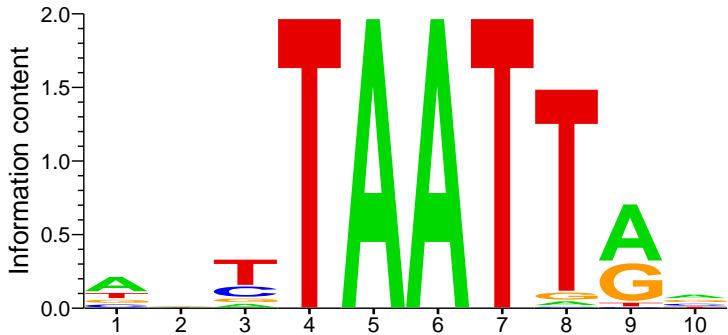

Supplement: Supplementary file 6 — Dataset EV1 [file MSB-13-910-s006.zip › Yang_Orenstein_DatasetEV1/homeodomain_LMX1B_TGAGGC20NGGA_TAAT_10_4.pwm.pdf]

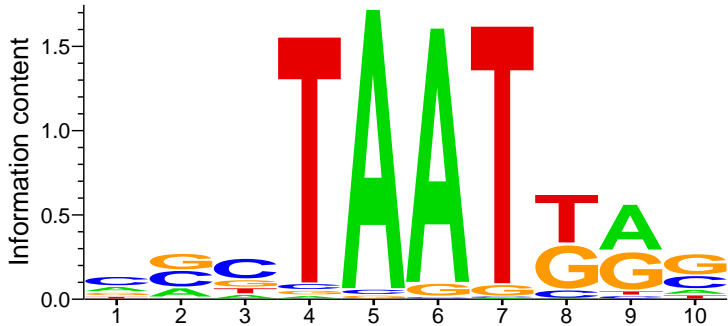

Supplement: Supplementary file 6 — Dataset EV1 [file MSB-13-910-s006.zip › Yang_Orenstein_DatasetEV1/homeodomain_MEOX1_TACCTA20NCG_TAAT_10_3.pwm.pdf]

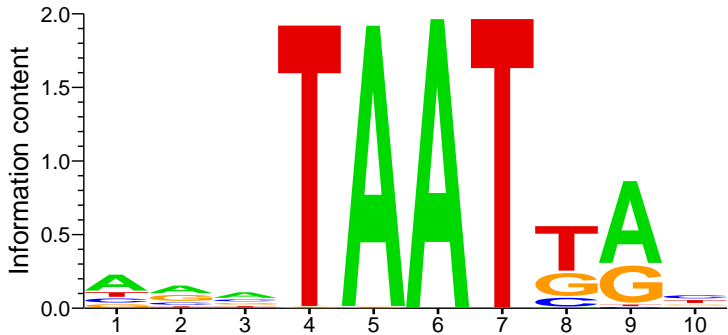

Supplement: Supplementary file 6 — Dataset EV1 [file MSB-13-910-s006.zip › Yang_Orenstein_DatasetEV1/homeodomain_Meox2_TACGTC20NTGC_TAAT_10_3.pwm.pdf]

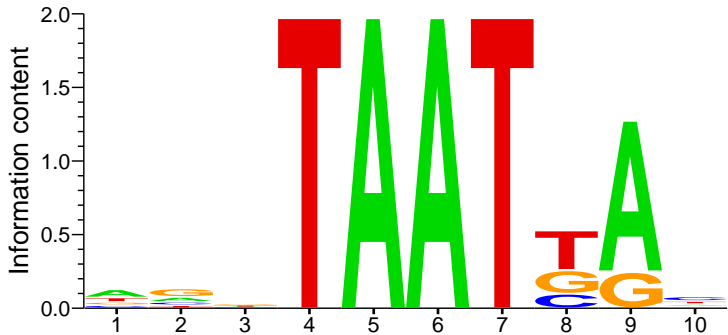

Supplement: Supplementary file 6 — Dataset EV1 [file MSB-13-910-s006.zip › Yang_Orenstein_DatasetEV1/homeodomain_MEOX2_TAGTTT30NTTT_TAAT_10_4.pwm.pdf]

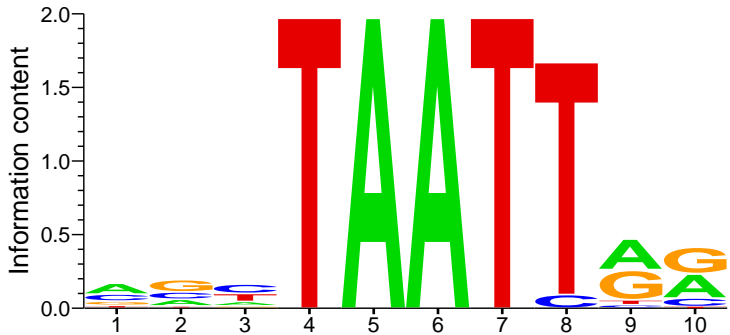

Supplement: Supplementary file 6 — Dataset EV1 [file MSB-13-910-s006.zip › Yang_Orenstein_DatasetEV1/homeodomain_MIXL1_TGATTT20NTTA_TAAT_10_4.pwm.pdf]

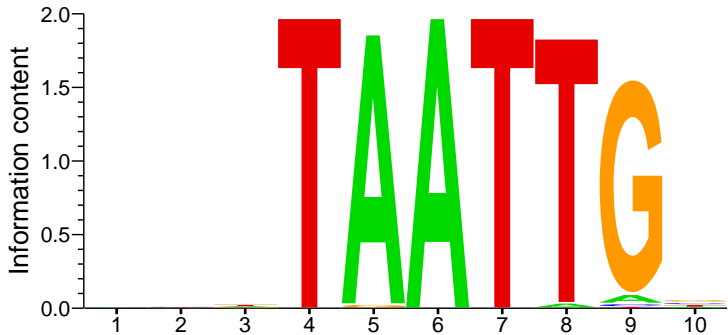

Supplement: Supplementary file 6 — Dataset EV1 [file MSB-13-910-s006.zip › Yang_Orenstein_DatasetEV1/homeodomain_MSX1_TGCGAA30NAGC_TAAT_10_5.pwm.pdf]

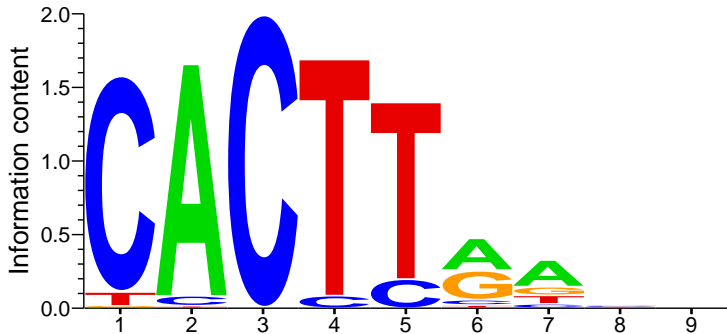

Supplement: Supplementary file 6 — Dataset EV1 [file MSB-13-910-s006.zip › Yang_Orenstein_DatasetEV1/homeodomain_NKX2-3_TGGAAT20NGA_ACTTRAN_9_4.pwm.pdf]

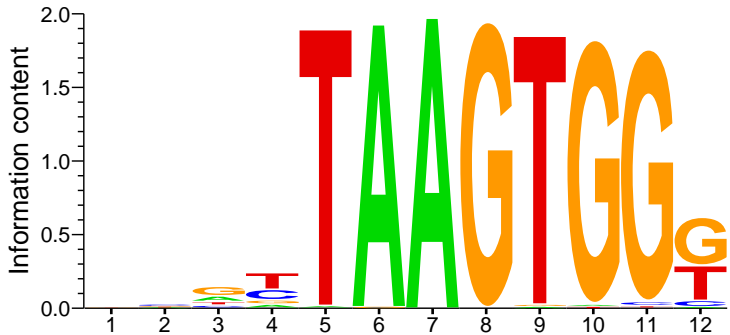

Supplement: Supplementary file 6 — Dataset EV1 [file MSB-13-910-s006.zip › Yang_Orenstein_DatasetEV1/homeodomain_NKX3-1_TCCCTC20NCG_TAAG_12_3.pwm.pdf]

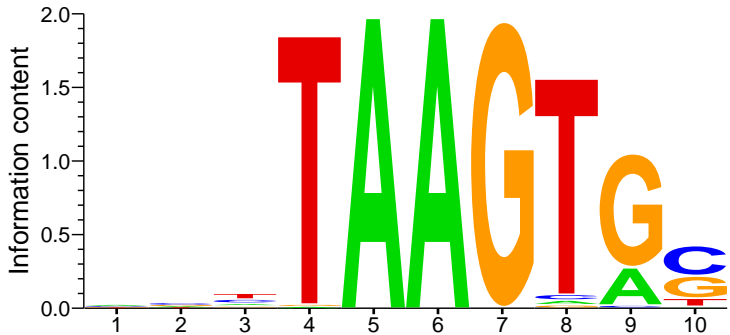

Supplement: Supplementary file 6 — Dataset EV1 [file MSB-13-910-s006.zip › Yang_Orenstein_DatasetEV1/homeodomain_Nkx3-1_TCTTAG20NATG_TAAG_10_4.pwm.pdf]

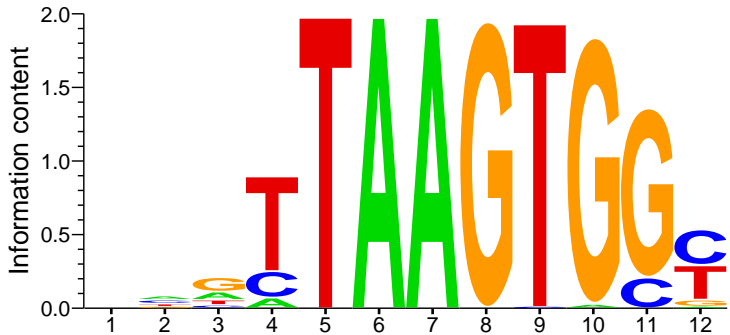

Supplement: Supplementary file 6 — Dataset EV1 [file MSB-13-910-s006.zip › Yang_Orenstein_DatasetEV1/homeodomain_NKX3-2_TCCAGT20NGAC_TAAG_12_4.pwm.pdf]

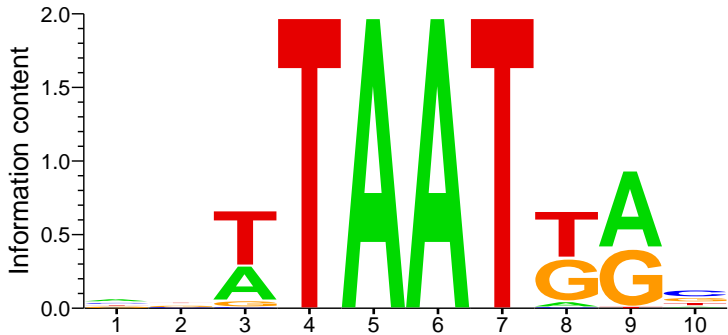

Supplement: Supplementary file 6 — Dataset EV1 [file MSB-13-910-s006.zip › Yang_Orenstein_DatasetEV1/homeodomain_NKX6-1_TGCGGG30NGGC_TAAT_10_4.pwm.pdf]

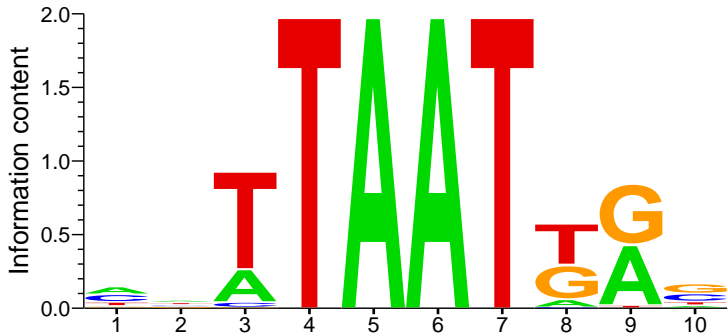

Supplement: Supplementary file 6 — Dataset EV1 [file MSB-13-910-s006.zip › Yang_Orenstein_DatasetEV1/homeodomain_NKX6-2_TTACAT20NTA_TAAT_10_4.pwm.pdf]

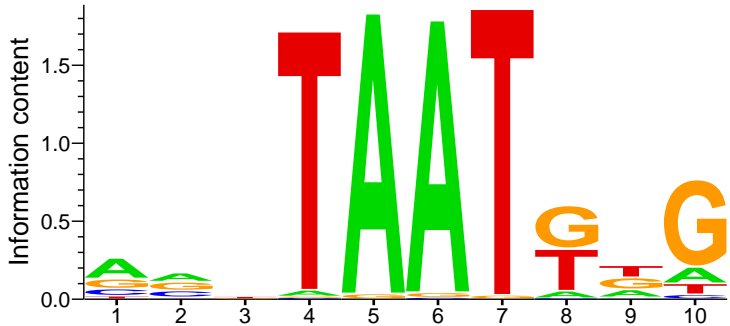

Supplement: Supplementary file 6 — Dataset EV1 [file MSB-13-910-s006.zip › Yang_Orenstein_DatasetEV1/homeodomain_NOTO_TGCGTT30NTGC_TAAT_10_3.pwm.pdf]

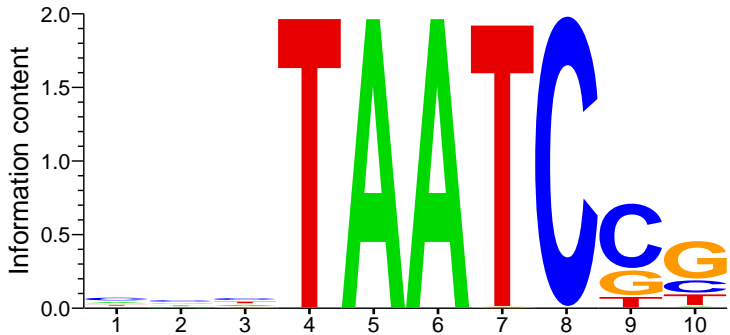

Supplement: Supplementary file 6 — Dataset EV1 [file MSB-13-910-s006.zip › Yang_Orenstein_DatasetEV1/homeodomain_OTX1_TGATAA20NGA_TAAT_10_4.pwm.pdf]

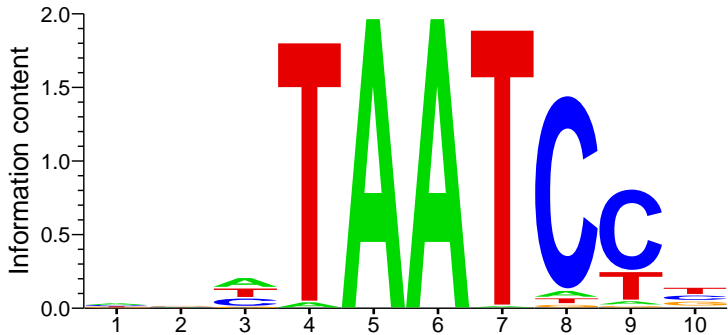

Supplement: Supplementary file 6 — Dataset EV1 [file MSB-13-910-s006.zip › Yang_Orenstein_DatasetEV1/homeodomain_OTX2_TATGGG20NGGG_TAAT_10_3.pwm.pdf]

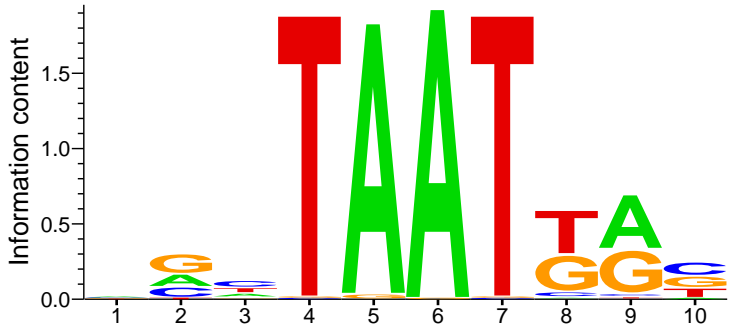

Supplement: Supplementary file 6 — Dataset EV1 [file MSB-13-910-s006.zip › Yang_Orenstein_DatasetEV1/homeodomain_PDX1_TGGAAT30NAAT_TAAT_10_3.pwm.pdf]

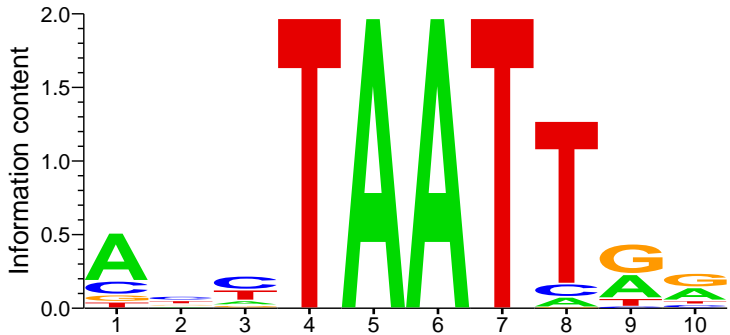

Supplement: Supplementary file 6 — Dataset EV1 [file MSB-13-910-s006.zip › Yang_Orenstein_DatasetEV1/homeodomain_PHOX2A_TGACTC20NGA_TAAT_10_3.pwm.pdf]

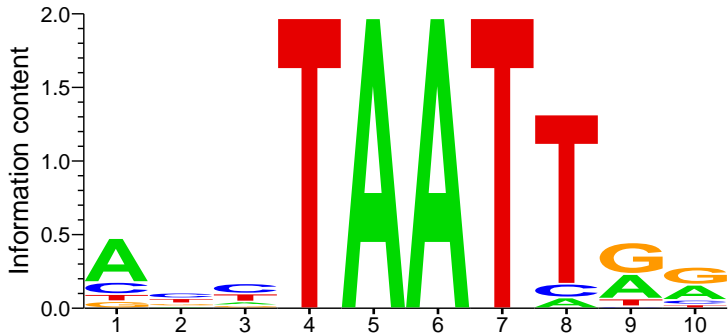

Supplement: Supplementary file 6 — Dataset EV1 [file MSB-13-910-s006.zip › Yang_Orenstein_DatasetEV1/homeodomain_PHOX2B_TGGTCT20NGA_TAAT_10_3.pwm.pdf]

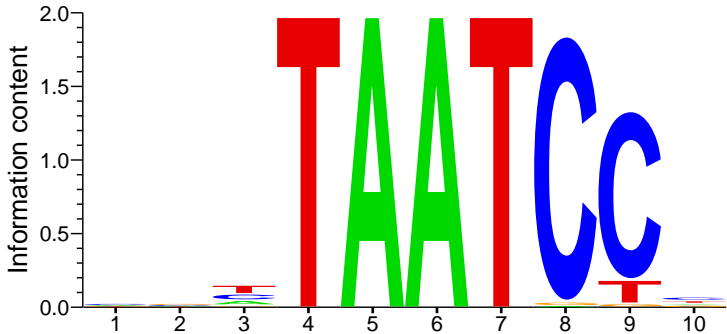

Supplement: Supplementary file 6 — Dataset EV1 [file MSB-13-910-s006.zip › Yang_Orenstein_DatasetEV1/homeodomain_PITX1_TGAGCA20NCGA_TAAT_10_4.pwm.pdf]

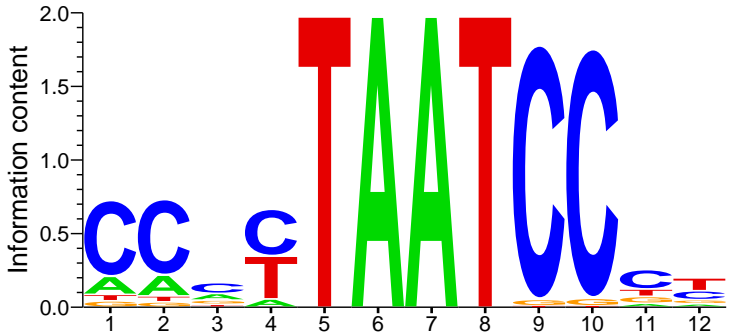

Supplement: Supplementary file 6 — Dataset EV1 [file MSB-13-910-s006.zip › Yang_Orenstein_DatasetEV1/homeodomain_PITX3_TGCATC20NGA_TAAT_12_3.pwm.pdf]

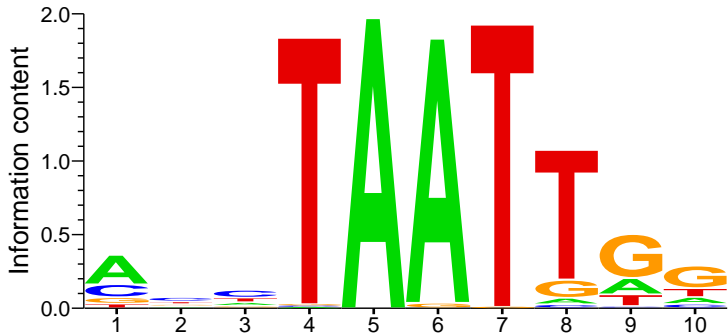

Supplement: Supplementary file 6 — Dataset EV1 [file MSB-13-910-s006.zip › Yang_Orenstein_DatasetEV1/homeodomain_PROP1_TGTATT20NGA_TAAT_10_3.pwm.pdf]

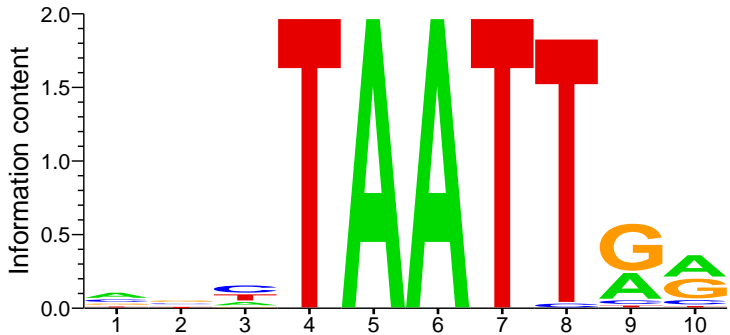

Supplement: Supplementary file 6 — Dataset EV1 [file MSB-13-910-s006.zip › Yang_Orenstein_DatasetEV1/homeodomain_PRRX1_TAACGG20NGCA_TAAT_10_3.pwm.pdf]

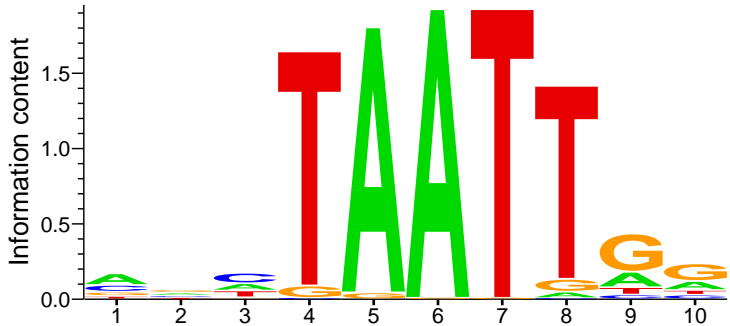

Supplement: Supplementary file 6 — Dataset EV1 [file MSB-13-910-s006.zip › Yang_Orenstein_DatasetEV1/homeodomain_Prrx2_TATGAA20NAGG_TAAT_10_3.pwm.pdf]

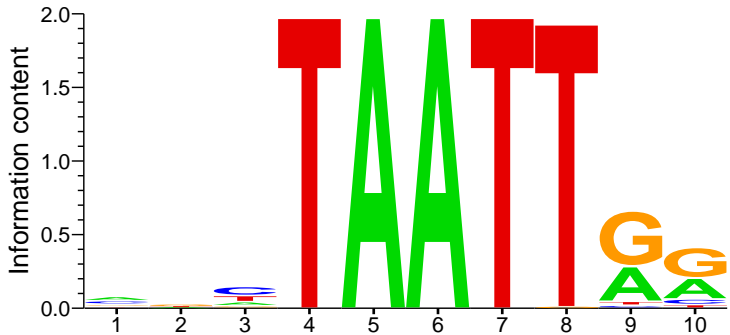

Supplement: Supplementary file 6 — Dataset EV1 [file MSB-13-910-s006.zip › Yang_Orenstein_DatasetEV1/homeodomain_PRRX2_TGGGGT20NGA_TAAT_10_4.pwm.pdf]

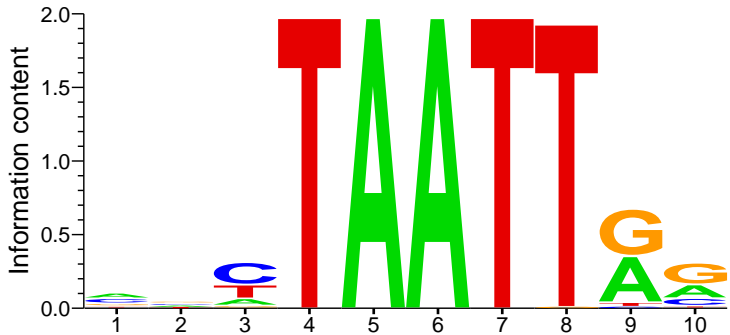

Supplement: Supplementary file 6 — Dataset EV1 [file MSB-13-910-s006.zip › Yang_Orenstein_DatasetEV1/homeodomain_RAXL1_TACTCA20NTA_TAAT_10_4.pwm.pdf]

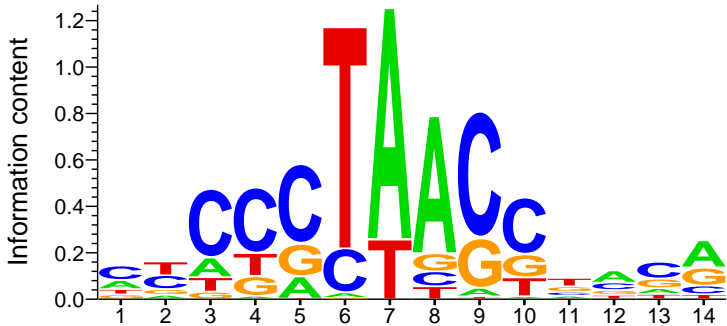

Supplement: Supplementary file 6 — Dataset EV1 [file MSB-13-910-s006.zip › Yang_Orenstein_DatasetEV1/homeodomain_Rhox11_TGGCCC20NCG_TWAN_14_4.pwm.pdf]

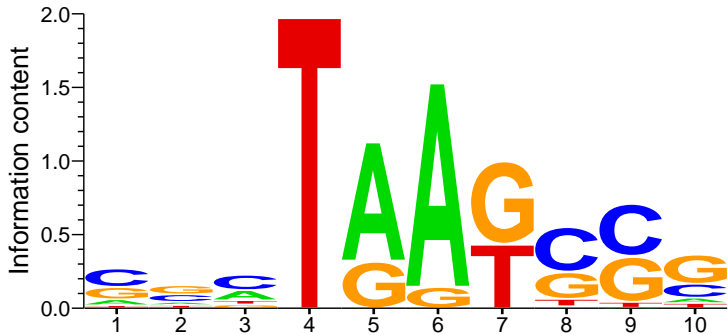

Supplement: Supplementary file 6 — Dataset EV1 [file MSB-13-910-s006.zip › Yang_Orenstein_DatasetEV1/homeodomain_RHOXF1_TTAGAA20NTA_TRAK_10_3.pwm.pdf]

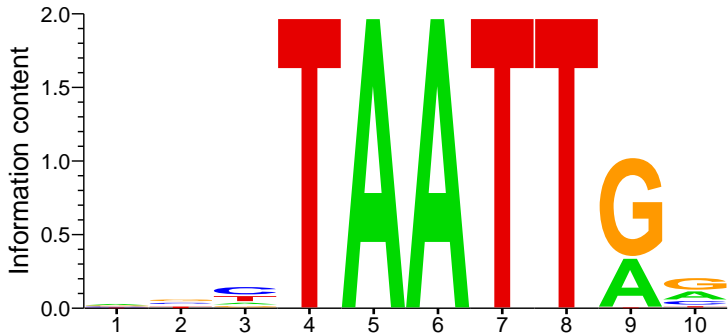

Supplement: Supplementary file 6 — Dataset EV1 [file MSB-13-910-s006.zip › Yang_Orenstein_DatasetEV1/homeodomain_SHOX2_TACGTC20NTGC_TAAT_10_3.pwm.pdf]

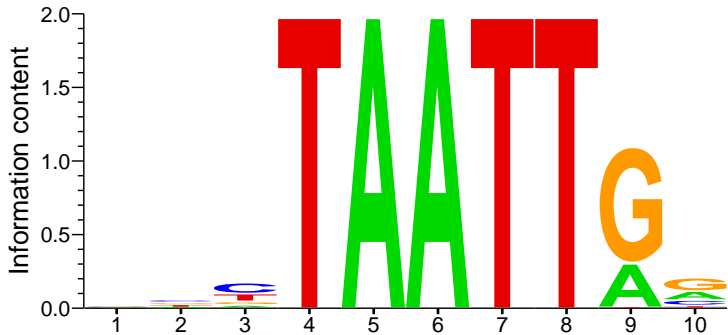

Supplement: Supplementary file 6 — Dataset EV1 [file MSB-13-910-s006.zip › Yang_Orenstein_DatasetEV1/homeodomain_Shox2_TCCCAG20NACC_TAAT_10_3.pwm.pdf]

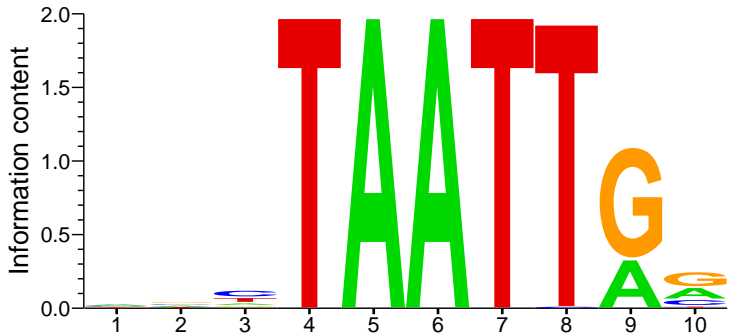

Supplement: Supplementary file 6 — Dataset EV1 [file MSB-13-910-s006.zip › Yang_Orenstein_DatasetEV1/homeodomain_SHOX_TAAGGT20NGGA_TAAT_10_3.pwm.pdf]

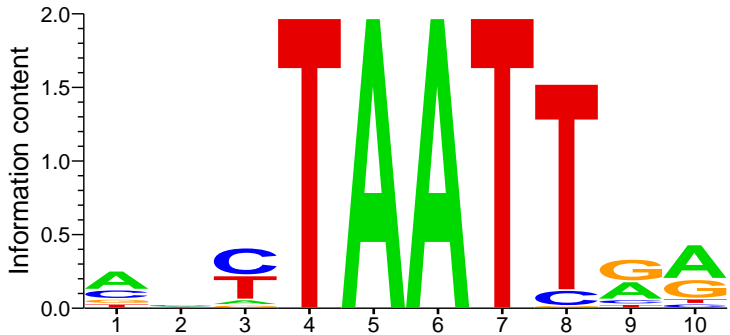

Supplement: Supplementary file 6 — Dataset EV1 [file MSB-13-910-s006.zip › Yang_Orenstein_DatasetEV1/homeodomain_UNCX_TGCAAG20NGA_TAAT_10_3.pwm.pdf]

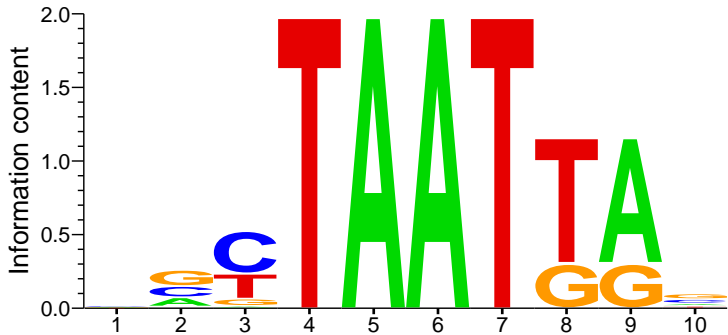

Supplement: Supplementary file 6 — Dataset EV1 [file MSB-13-910-s006.zip › Yang_Orenstein_DatasetEV1/homeodomain_VAX1_TGCCAT20NACC_TAAT_10_4.pwm.pdf]

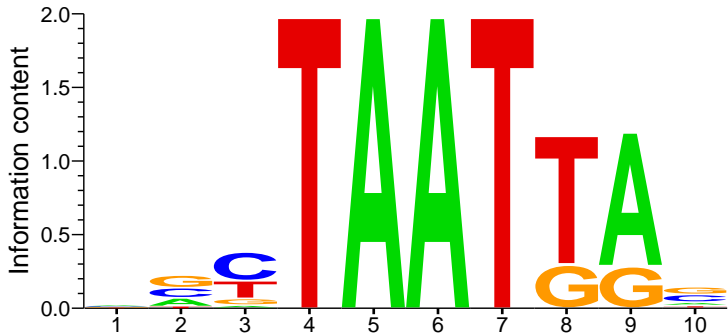

Supplement: Supplementary file 6 — Dataset EV1 [file MSB-13-910-s006.zip › Yang_Orenstein_DatasetEV1/homeodomain_VAX2_TGGTAG20NATT_TAAT_10_4.pwm.pdf]

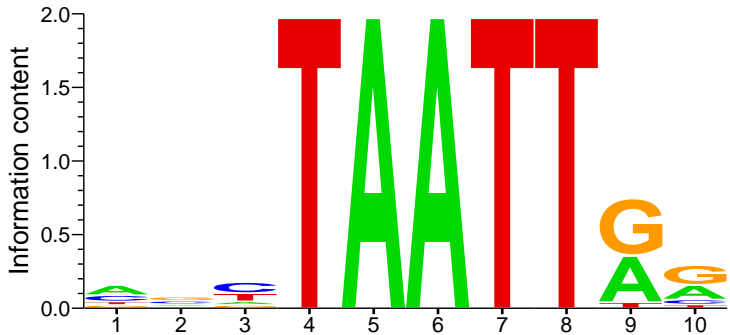

Supplement: Supplementary file 6 — Dataset EV1 [file MSB-13-910-s006.zip › Yang_Orenstein_DatasetEV1/homeodomain_Vsx1_TGCAGA20NCG_TAAT_10_4.pwm.pdf]

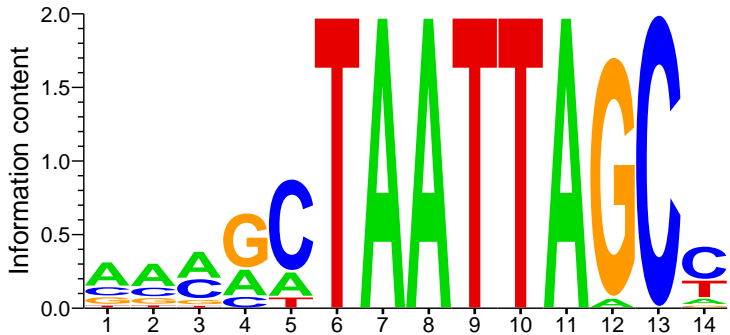

Supplement: Supplementary file 6 — Dataset EV1 [file MSB-13-910-s006.zip › Yang_Orenstein_DatasetEV1/homeodomain_VSX1_TGGTTC20NTTT_TAAT_14_3.pwm.pdf]

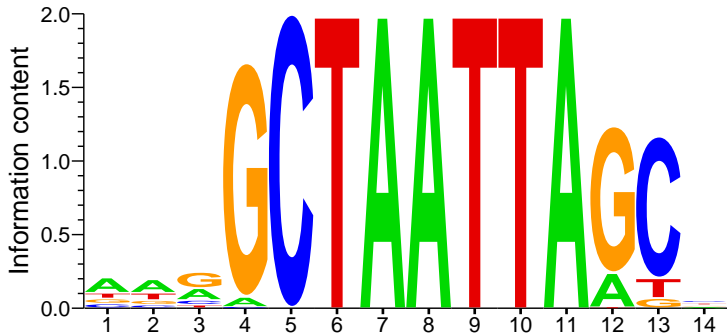

Supplement: Supplementary file 6 — Dataset EV1 [file MSB-13-910-s006.zip › Yang_Orenstein_DatasetEV1/homeodomain_VSX2_TCTCCC20NCCG_TAAT_14_4.pwm.pdf]

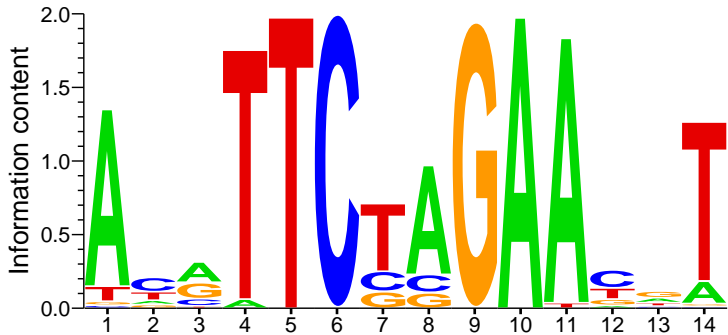

Supplement: Supplementary file 6 — Dataset EV1 [file MSB-13-910-s006.zip › Yang_Orenstein_DatasetEV1/HSF_HSF1_TATAGA20NGAG_TTCTAGAA_14_3.pwm.pdf]

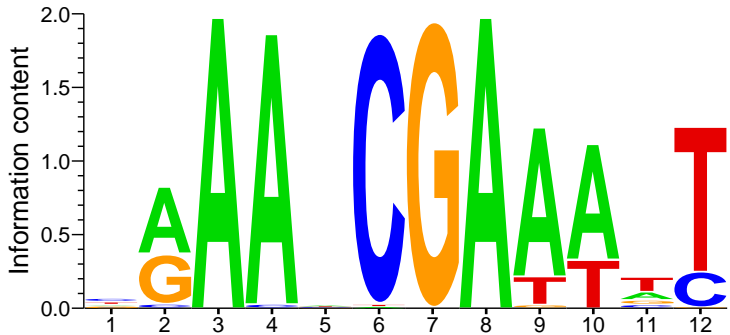

Supplement: Supplementary file 6 — Dataset EV1 [file MSB-13-910-s006.zip › Yang_Orenstein_DatasetEV1/IRF_IRF7_TATCGC20NGCG_RAANCGAAAW_12_3.pwm.pdf]

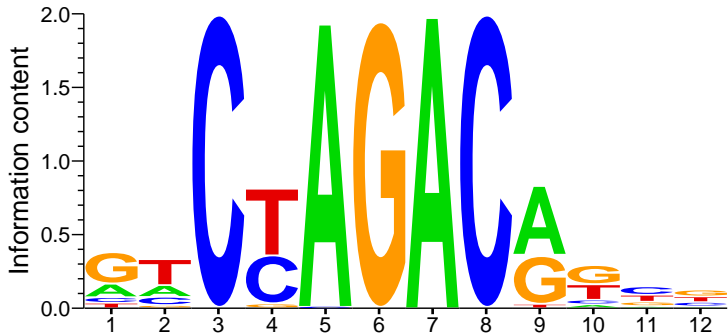

Supplement: Supplementary file 6 — Dataset EV1 [file MSB-13-910-s006.zip › Yang_Orenstein_DatasetEV1/MAD_SMAD3_TGGGTA20NGA_TAGACA_12_3.pwm.pdf]

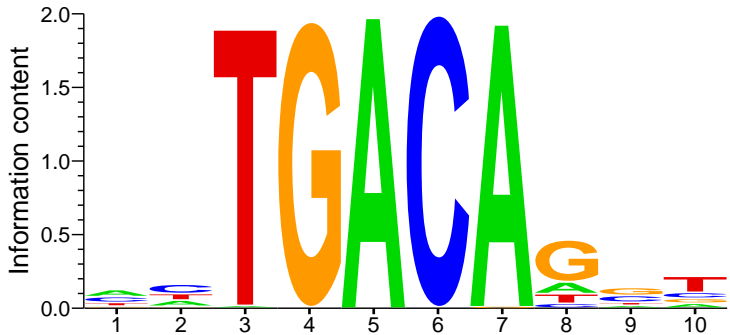

Supplement: Supplementary file 6 — Dataset EV1 [file MSB-13-910-s006.zip › Yang_Orenstein_DatasetEV1/MEIS_MEIS1_TGACCT20NGA_TGACAN_10_5.pwm.pdf]

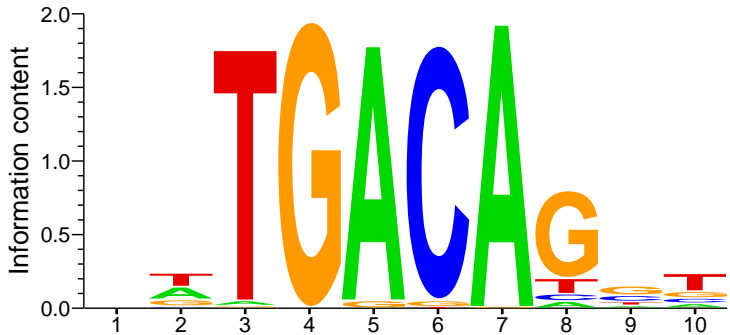

Supplement: Supplementary file 6 — Dataset EV1 [file MSB-13-910-s006.zip › Yang_Orenstein_DatasetEV1/MEIS_Meis2_TCAAAA20NTA_TGACAG_10_5.pwm.pdf]

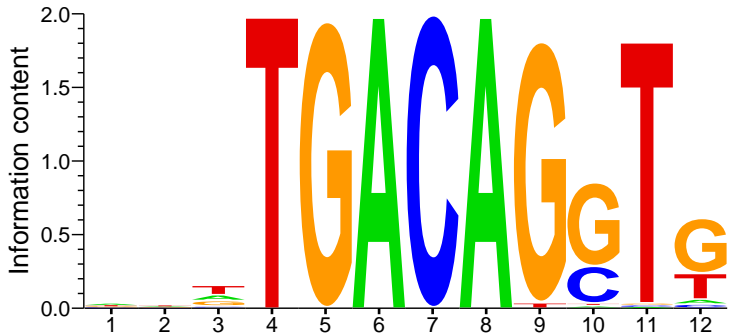

Supplement: Supplementary file 6 — Dataset EV1 [file MSB-13-910-s006.zip › Yang_Orenstein_DatasetEV1/MEIS_MEIS3_TGGGTA20NGA_TGACAG_12_3.pwm.pdf]

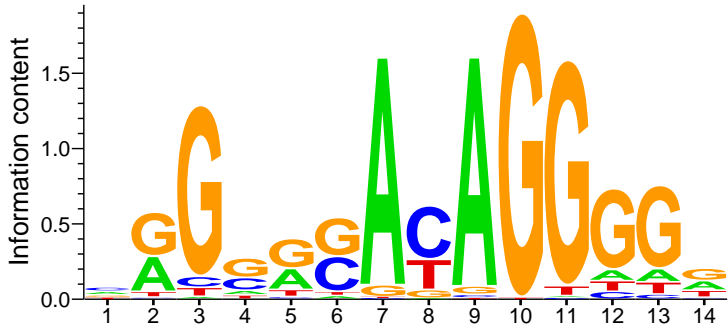

Supplement: Supplementary file 6 — Dataset EV1 [file MSB-13-910-s006.zip › Yang_Orenstein_DatasetEV1/MEIS_Pknox2_TGCTCG20NCG_TGACAG_14_3.pwm.pdf]

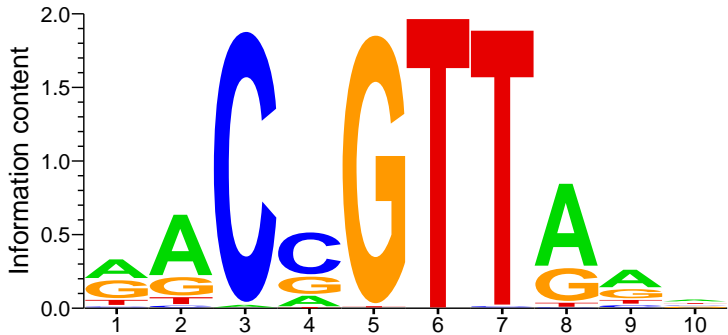

Supplement: Supplementary file 6 — Dataset EV1 [file MSB-13-910-s006.zip › Yang_Orenstein_DatasetEV1/MYB_MYBL1_TTGCGT20NTA_CNGTTA_10_4.pwm.pdf]

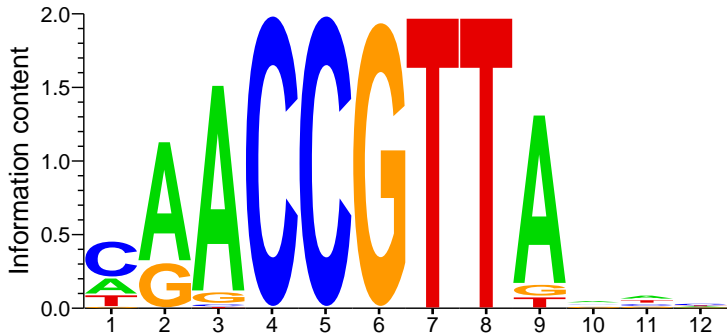

Supplement: Supplementary file 6 — Dataset EV1 [file MSB-13-910-s006.zip › Yang_Orenstein_DatasetEV1/MYB_MYBL2_TGAGTG20NGA_CCGTTN_12_3.pwm.pdf]

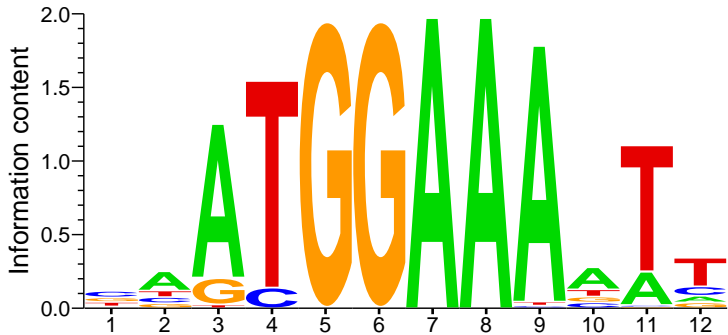

Supplement: Supplementary file 6 — Dataset EV1 [file MSB-13-910-s006.zip › Yang_Orenstein_DatasetEV1/NFAT_NFATC1_TTCGTA20NTGC_TGGAAA_12_4.pwm.pdf]

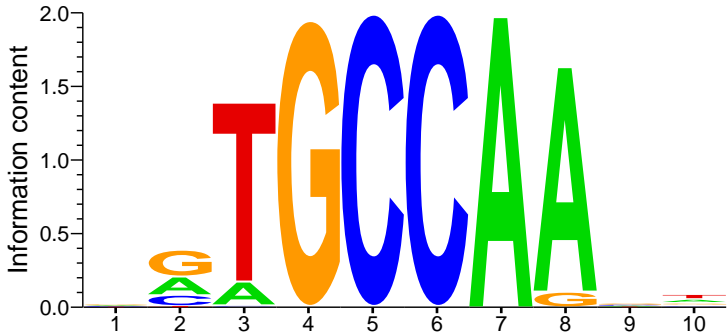

Supplement: Supplementary file 6 — Dataset EV1 [file MSB-13-910-s006.zip › Yang_Orenstein_DatasetEV1/NFI_NFIA_TACGTC20NTGC_TGCCAA_10_4.pwm.pdf]

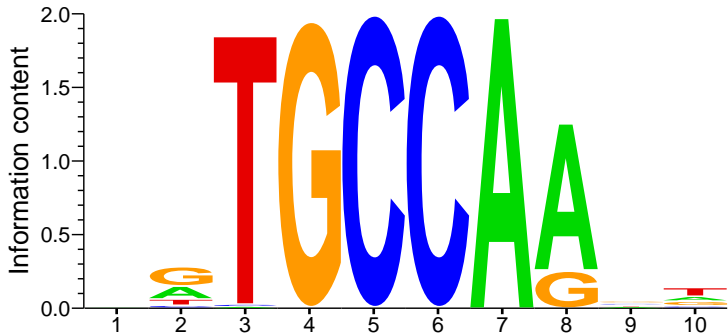

Supplement: Supplementary file 6 — Dataset EV1 [file MSB-13-910-s006.zip › Yang_Orenstein_DatasetEV1/NFI_NFIX_TGAGAT20NAGA_TGCCAA_10_4.pwm.pdf]

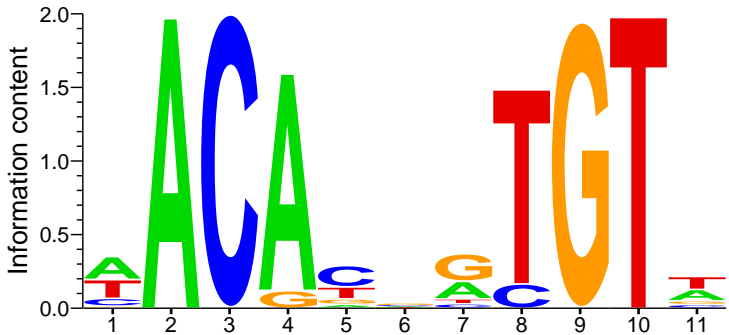

Supplement: Supplementary file 6 — Dataset EV1 [file MSB-13-910-s006.zip › Yang_Orenstein_DatasetEV1/nuclearreceptor_Ar_TCTAAT20NCG_ACANNNTGT_11_5.pwm.pdf]

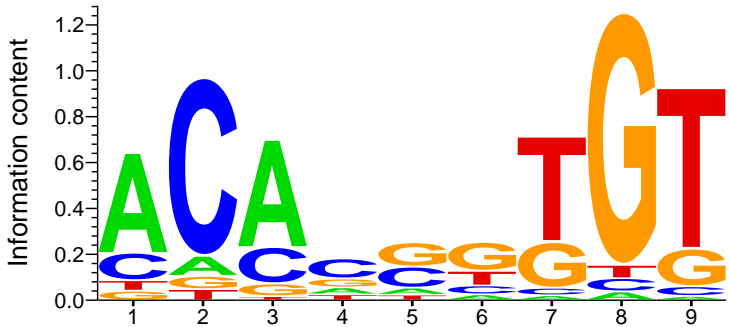

Supplement: Supplementary file 6 — Dataset EV1 [file MSB-13-910-s006.zip › Yang_Orenstein_DatasetEV1/nuclearreceptor_AR_TGCTCG20NGA_ACANNNTGT_9_3.pwm.pdf]

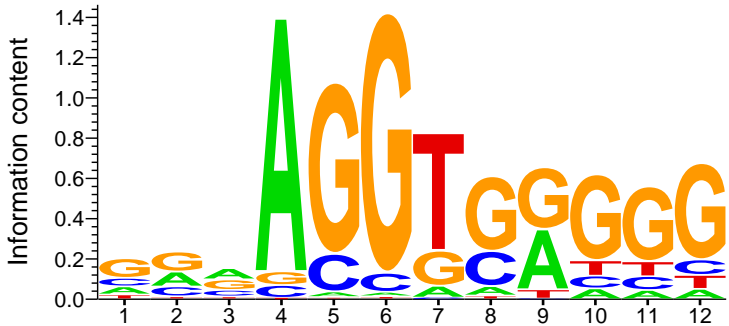

Supplement: Supplementary file 6 — Dataset EV1 [file MSB-13-910-s006.zip › Yang_Orenstein_DatasetEV1/nuclearreceptor_ESR1_TAGAGT20NCG_AGGTCA_12_3.pwm.pdf]

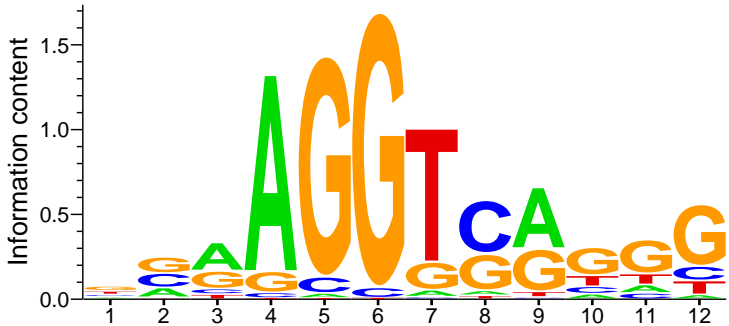

Supplement: Supplementary file 6 — Dataset EV1 [file MSB-13-910-s006.zip › Yang_Orenstein_DatasetEV1/nuclearreceptor_Esrra_TAGCTC20NCG_AGGTCA_12_3.pwm.pdf]

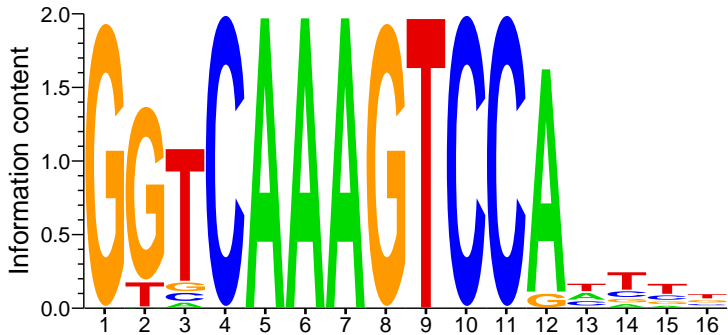

Supplement: Supplementary file 6 — Dataset EV1 [file MSB-13-910-s006.zip › Yang_Orenstein_DatasetEV1/nuclearreceptor_HNF4A_TCCGTG40NTGC_AAGTCC_16_3.pwm.pdf]

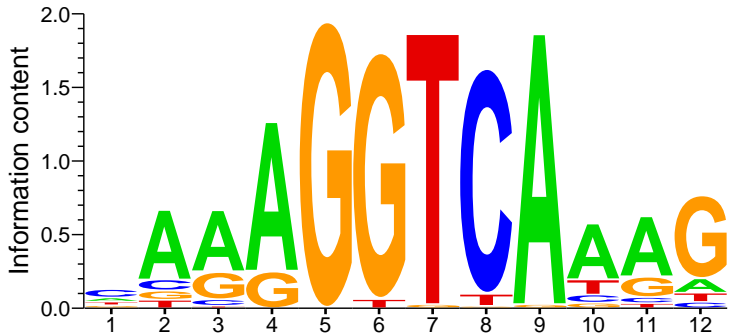

Supplement: Supplementary file 6 — Dataset EV1 [file MSB-13-910-s006.zip › Yang_Orenstein_DatasetEV1/nuclearreceptor_NR2C2_TCGACT20NCAT_AGGTCA_12_4.pwm.pdf]

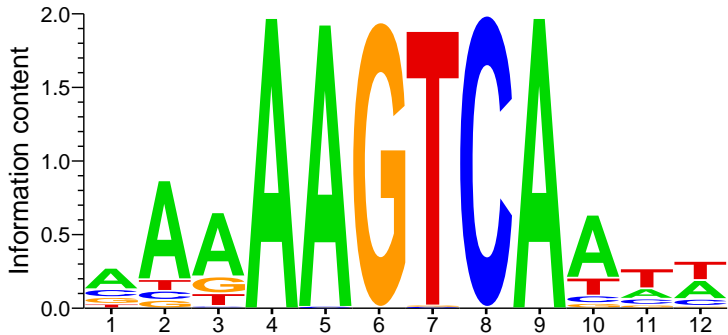

Supplement: Supplementary file 6 — Dataset EV1 [file MSB-13-910-s006.zip › Yang_Orenstein_DatasetEV1/nuclearreceptor_Nr2e1_TCGCGC20NGCT_AAGTCA_12_3.pwm.pdf]

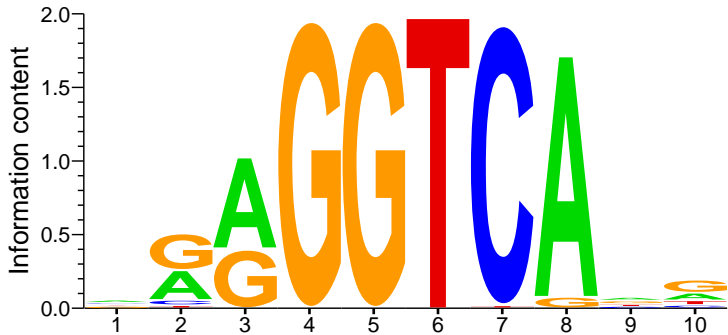

Supplement: Supplementary file 6 — Dataset EV1 [file MSB-13-910-s006.zip › Yang_Orenstein_DatasetEV1/nuclearreceptor_NR2F1_TCTGAC20NGA_RGGTCA_10_3.pwm.pdf]

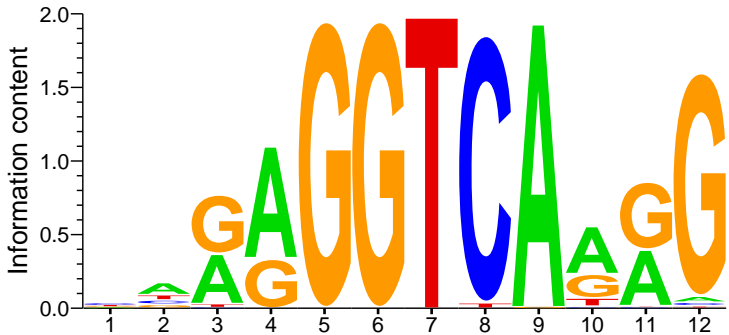

Supplement: Supplementary file 6 — Dataset EV1 [file MSB-13-910-s006.zip › Yang_Orenstein_DatasetEV1/nuclearreceptor_NR2F6_TCTTGA20NGA_AGGTCA_12_3.pwm.pdf]

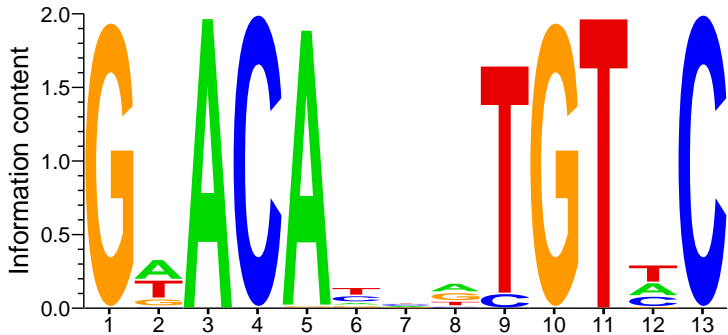

Supplement: Supplementary file 6 — Dataset EV1 [file MSB-13-910-s006.zip › Yang_Orenstein_DatasetEV1/nuclearreceptor_NR3C1_TGACGA20NGCA_ACANNNTGT_13_3.pwm.pdf]

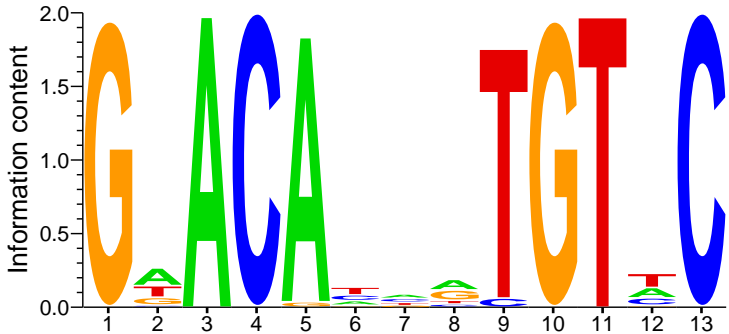

Supplement: Supplementary file 6 — Dataset EV1 [file MSB-13-910-s006.zip › Yang_Orenstein_DatasetEV1/nuclearreceptor_NR3C2_TCAGAG20NAGA_ACANNNTGT_13_4.pwm.pdf]

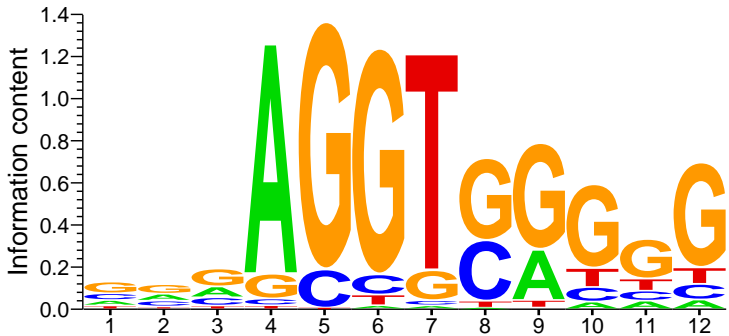

Supplement: Supplementary file 6 — Dataset EV1 [file MSB-13-910-s006.zip › Yang_Orenstein_DatasetEV1/nuclearreceptor_RARA_TATAGA20NCG_AGGTCA_12_3.pwm.pdf]

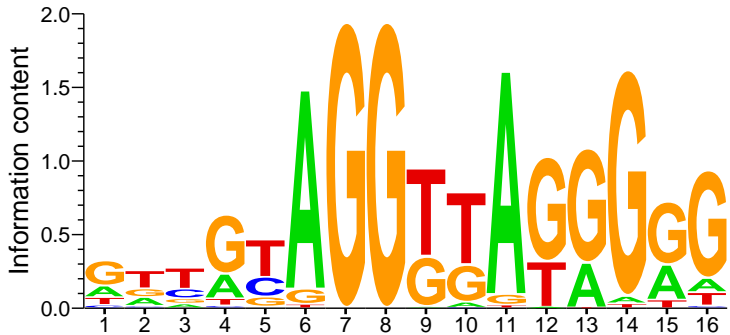

Supplement: Supplementary file 6 — Dataset EV1 [file MSB-13-910-s006.zip › Yang_Orenstein_DatasetEV1/nuclearreceptor_Rara_TCGGTT20NCG_AGGTCA_16_4.pwm.pdf]

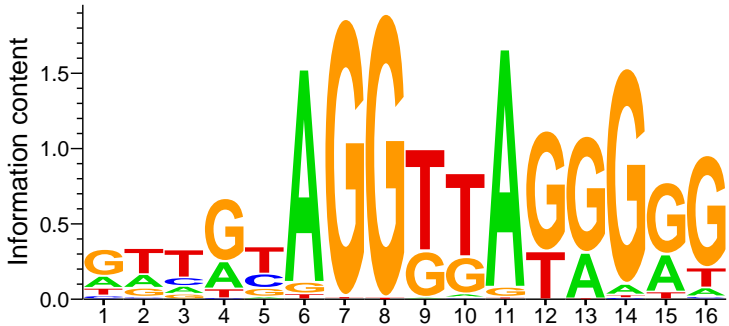

Supplement: Supplementary file 6 — Dataset EV1 [file MSB-13-910-s006.zip › Yang_Orenstein_DatasetEV1/nuclearreceptor_Rarb_TCGTGT20NCG_AGGTCA_16_4.pwm.pdf]

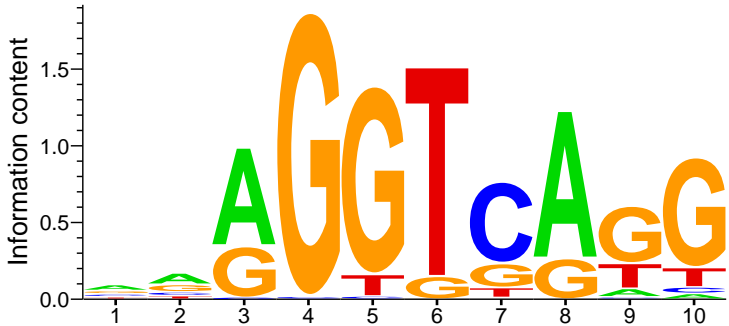

Supplement: Supplementary file 6 — Dataset EV1 [file MSB-13-910-s006.zip › Yang_Orenstein_DatasetEV1/nuclearreceptor_RARG_TCGGGG20NGA_RGGTCA_10_3.pwm.pdf]

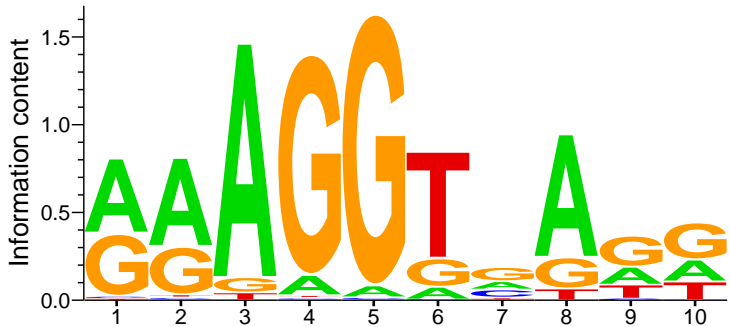

Supplement: Supplementary file 6 — Dataset EV1 [file MSB-13-910-s006.zip › Yang_Orenstein_DatasetEV1/nuclearreceptor_RORA_TCTGTA30NTGG_AGGTCA_10_3.pwm.pdf]

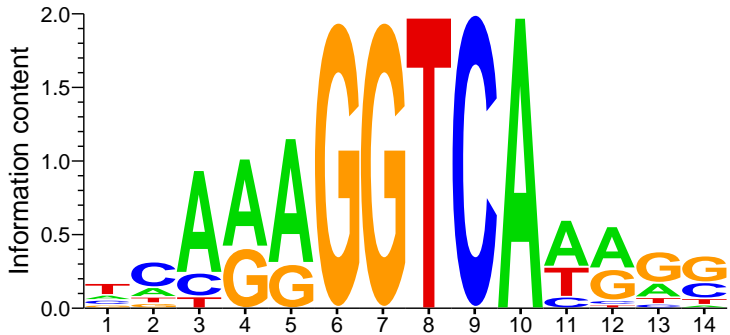

Supplement: Supplementary file 6 — Dataset EV1 [file MSB-13-910-s006.zip › Yang_Orenstein_DatasetEV1/nuclearreceptor_RXRA_TCTACA40NCAG_RGGTCA_14_3.pwm.pdf]

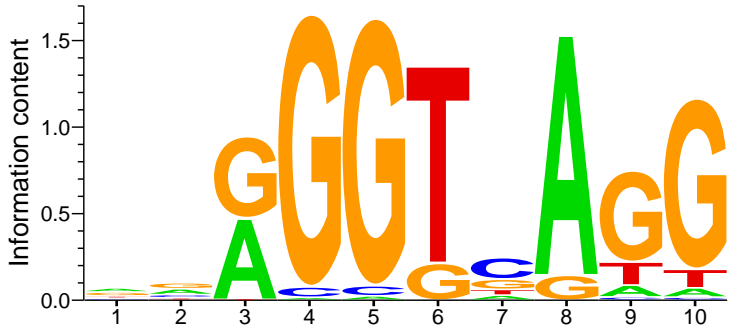

Supplement: Supplementary file 6 — Dataset EV1 [file MSB-13-910-s006.zip › Yang_Orenstein_DatasetEV1/nuclearreceptor_RXRG_TTCTCT20NTA_RGGTCA_10_3.pwm.pdf]

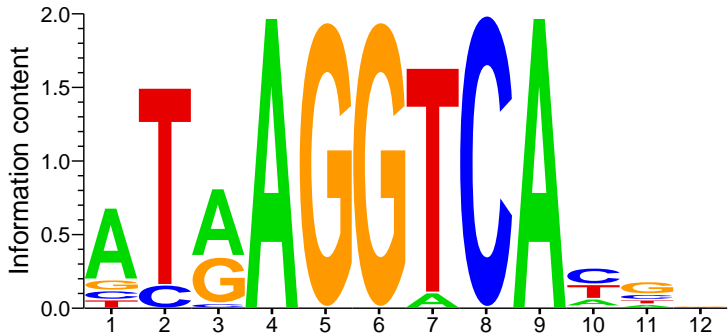

Supplement: Supplementary file 6 — Dataset EV1 [file MSB-13-910-s006.zip › Yang_Orenstein_DatasetEV1/nuclearreceptor_THRA_TCGGCC40NCGT_AGGTCA_12_3.pwm.pdf]

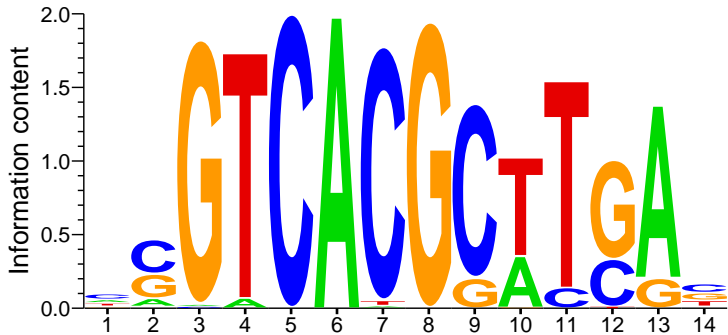

Supplement: Supplementary file 6 — Dataset EV1 [file MSB-13-910-s006.zip › Yang_Orenstein_DatasetEV1/PAX_PAX2_TCCACG20NCAC_CACGCW_14_4.pwm.pdf]

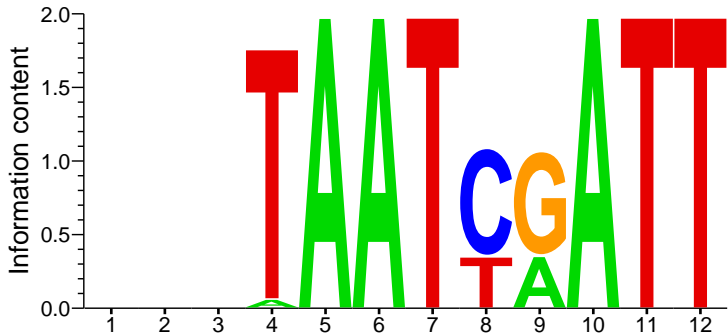

Supplement: Supplementary file 6 — Dataset EV1 [file MSB-13-910-s006.zip › Yang_Orenstein_DatasetEV1/PAX_PAX7_TCCAAC20NAAC_TAATYR_12_4.pwm.pdf]

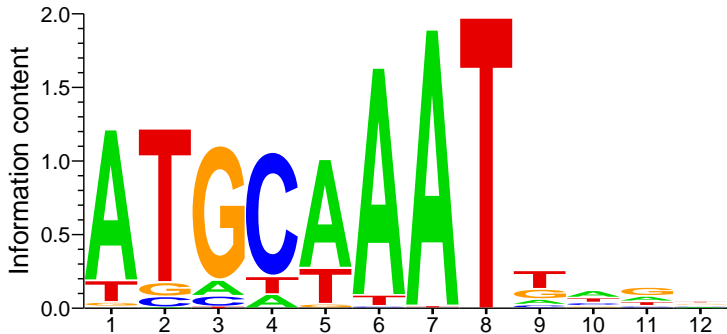

Supplement: Supplementary file 6 — Dataset EV1 [file MSB-13-910-s006.zip › Yang_Orenstein_DatasetEV1/POU_POU2F1_TCTTTC20NGA_WAAT_12_4.pwm.pdf]

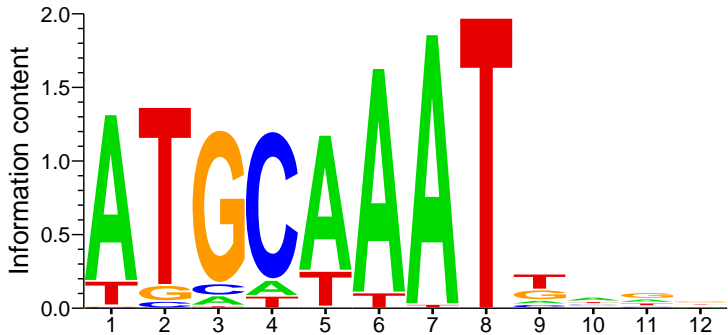

Supplement: Supplementary file 6 — Dataset EV1 [file MSB-13-910-s006.zip › Yang_Orenstein_DatasetEV1/POU_POU2F2_TGACAG20NGA_WAAT_12_4.pwm.pdf]

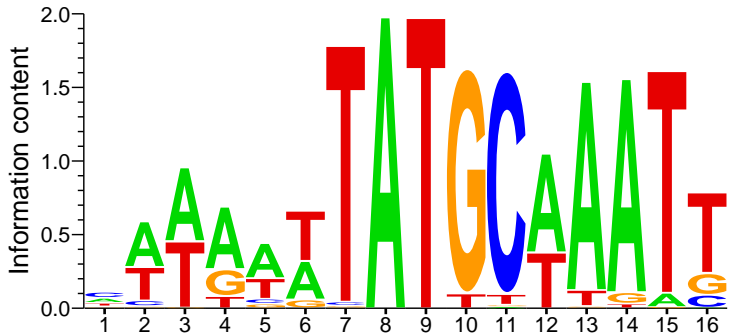

Supplement: Supplementary file 6 — Dataset EV1 [file MSB-13-910-s006.zip › Yang_Orenstein_DatasetEV1/POU_POU2F3_TAGACG20NCAT_TATK_16_3.pwm.pdf]
