# Supplementary figures and images for: Transcription factor family‐specific DNA shape readout revealed by quantitative specificity models (part 1 of 4)
Source: Mol Syst Biol. 2017 Feb 6;13(2):910. doi: 10.15252/msb.20167238 (PMC5327724; doi:10.15252/msb.20167238)

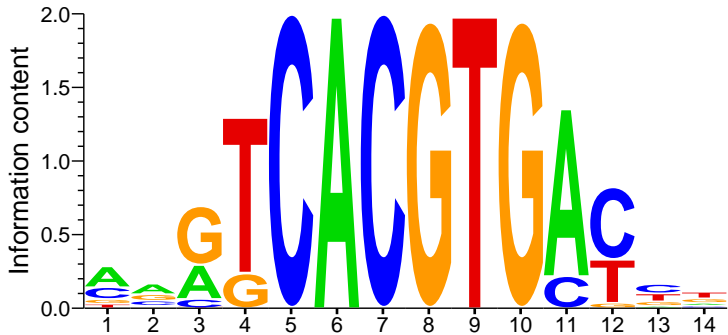

Supplement: Supplementary file 6 — Dataset EV1 [file MSB-13-910-s006.zip › Yang_Orenstein_DatasetEV1/bHLH_Bhlhb2_TCAAGG20NGAA_CACGTG_14_4.pwm.pdf]

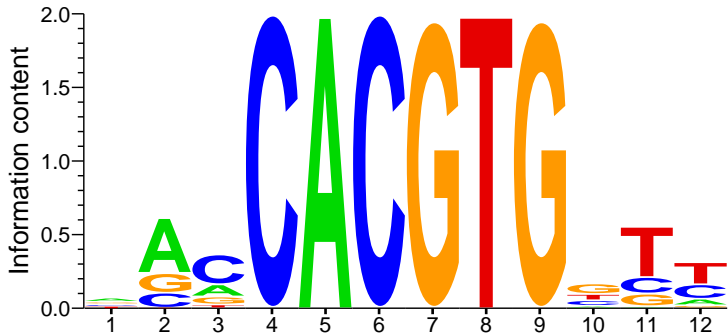

Supplement: Supplementary file 6 — Dataset EV1 [file MSB-13-910-s006.zip › Yang_Orenstein_DatasetEV1/bHLH_MAX_TGACCT20NGA_CACGTG_12_3.pwm.pdf]

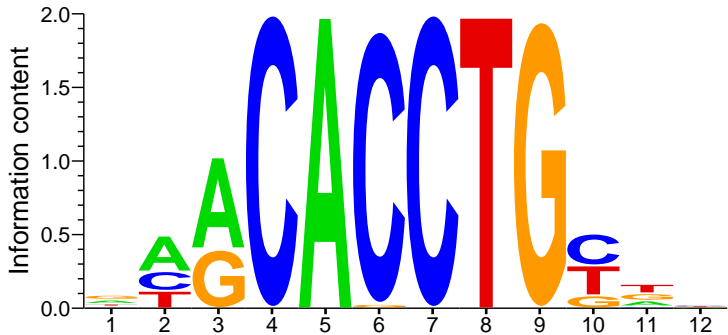

Supplement: Supplementary file 6 — Dataset EV1 [file MSB-13-910-s006.zip › Yang_Orenstein_DatasetEV1/bHLH_TCF3_TACCCG20NCCC_CACCTG_12_3.pwm.pdf]

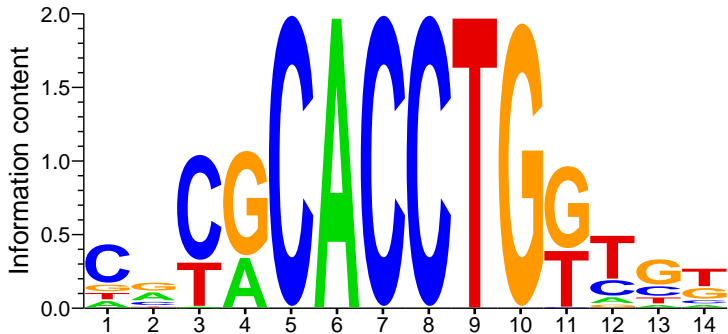

Supplement: Supplementary file 6 — Dataset EV1 [file MSB-13-910-s006.zip › Yang_Orenstein_DatasetEV1/bHLH_TCF4_TGCGAA20NGA_CACCTG_14_3.pwm.pdf]

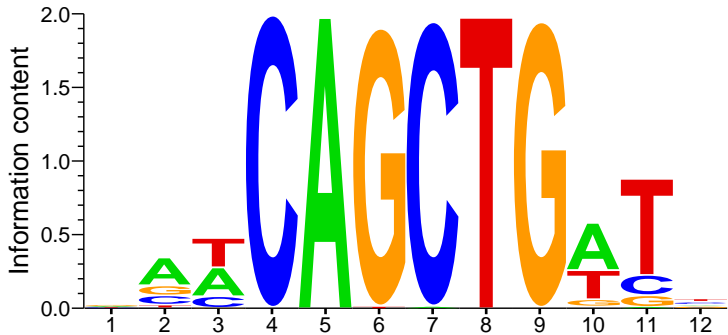

Supplement: Supplementary file 6 — Dataset EV1 [file MSB-13-910-s006.zip › Yang_Orenstein_DatasetEV1/bHLH_TFAP4_TCACGT20NGCA_CAGCTG_12_4.pwm.pdf]

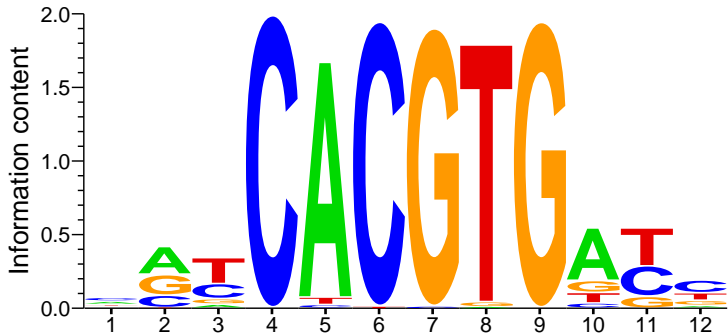

Supplement: Supplementary file 6 — Dataset EV1 [file MSB-13-910-s006.zip › Yang_Orenstein_DatasetEV1/bHLH_TFE3_TACCGT20NTA_CACGTG_12_3.pwm.pdf]

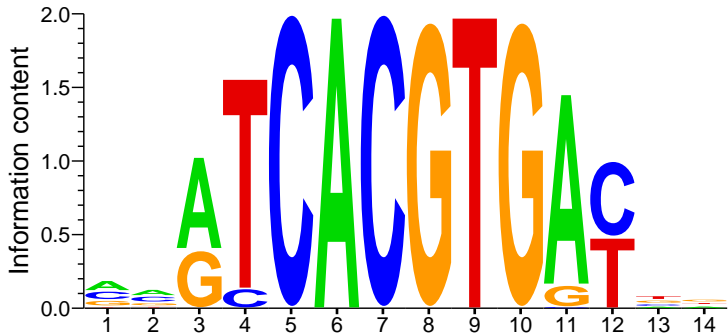

Supplement: Supplementary file 6 — Dataset EV1 [file MSB-13-910-s006.zip › Yang_Orenstein_DatasetEV1/bHLH_TFEB_TAGTTT20NCG_CACGTG_14_4.pwm.pdf]

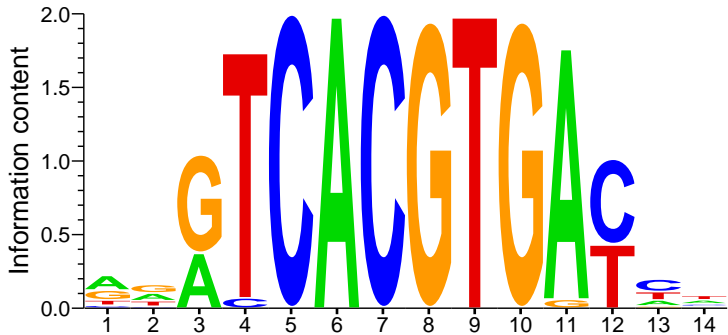

Supplement: Supplementary file 6 — Dataset EV1 [file MSB-13-910-s006.zip › Yang_Orenstein_DatasetEV1/bHLH_USF1_TGACGA20NGCA_CACGTG_14_4.pwm.pdf]

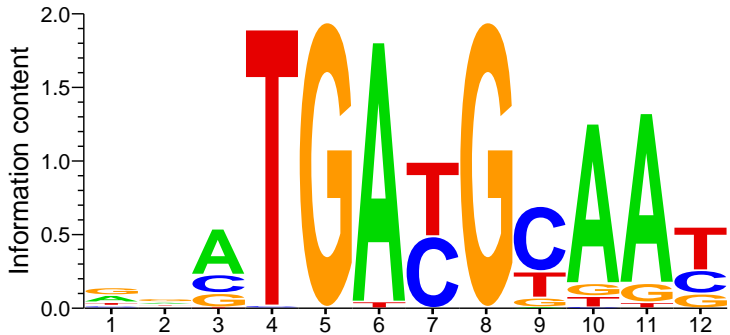

Supplement: Supplementary file 6 — Dataset EV1 [file MSB-13-910-s006.zip › Yang_Orenstein_DatasetEV1/bZIP_ATF4_TGGAAT20NGA_TGAYGC_12_3.pwm.pdf]

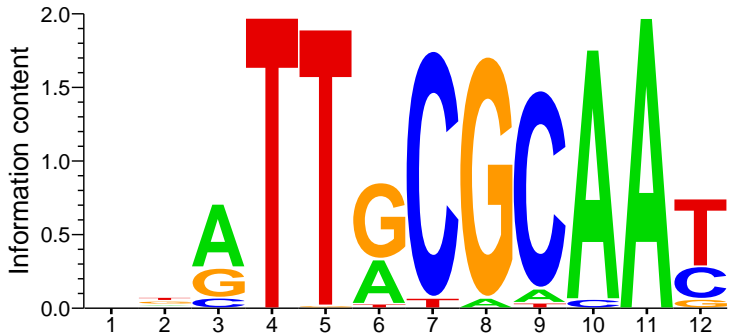

Supplement: Supplementary file 6 — Dataset EV1 [file MSB-13-910-s006.zip › Yang_Orenstein_DatasetEV1/bZIP_Cebpb_TCCTAA20NATC_TTRCGC_12_3.pwm.pdf]

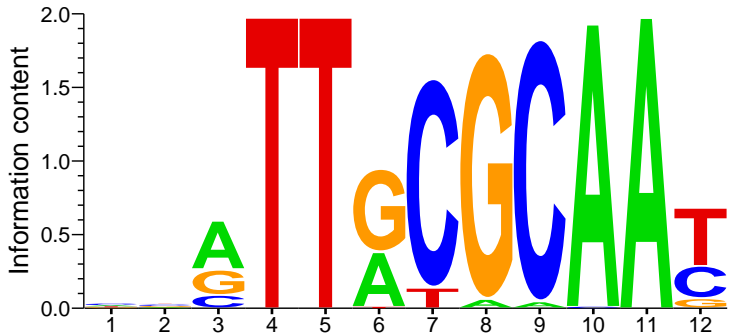

Supplement: Supplementary file 6 — Dataset EV1 [file MSB-13-910-s006.zip › Yang_Orenstein_DatasetEV1/bZIP_CEBPB_TGGACA20NGA_TTRCGC_12_4.pwm.pdf]

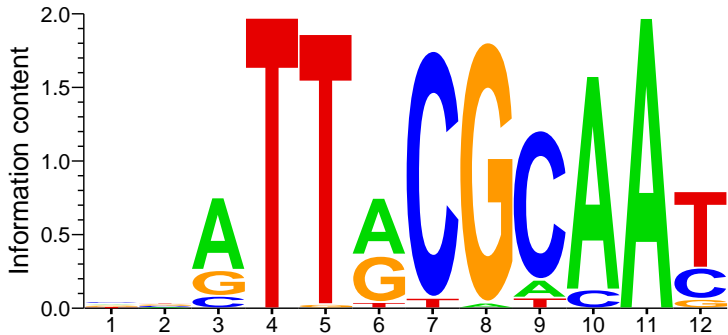

Supplement: Supplementary file 6 — Dataset EV1 [file MSB-13-910-s006.zip › Yang_Orenstein_DatasetEV1/bZIP_CEBPD_TAATGA20NCG_TTRCGC_12_3.pwm.pdf]

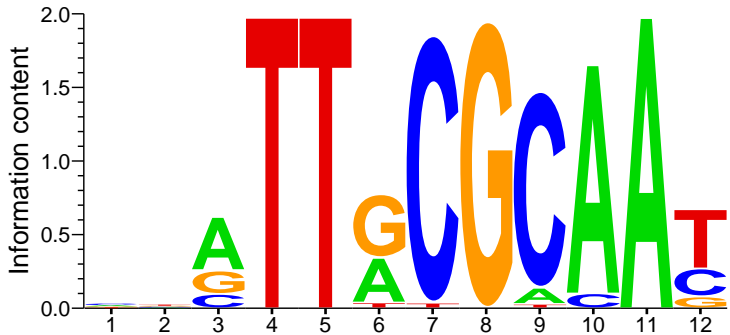

Supplement: Supplementary file 6 — Dataset EV1 [file MSB-13-910-s006.zip › Yang_Orenstein_DatasetEV1/bZIP_CEBPE_TGAGGC20NGA_TTRCGC_12_4.pwm.pdf]

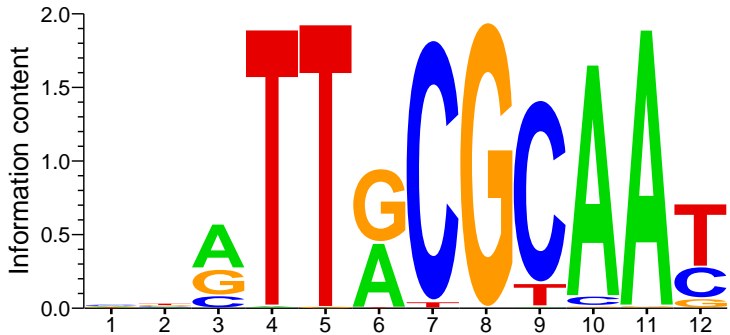

Supplement: Supplementary file 6 — Dataset EV1 [file MSB-13-910-s006.zip › Yang_Orenstein_DatasetEV1/bZIP_CEBPG_TAGACG20NTA_TTRCGC_12_3.pwm.pdf]

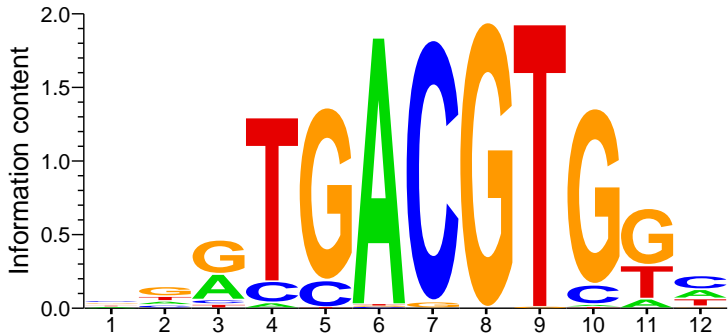

Supplement: Supplementary file 6 — Dataset EV1 [file MSB-13-910-s006.zip › Yang_Orenstein_DatasetEV1/bZIP_CREB3_TAGAAC20NCG_TGACGT_12_4.pwm.pdf]

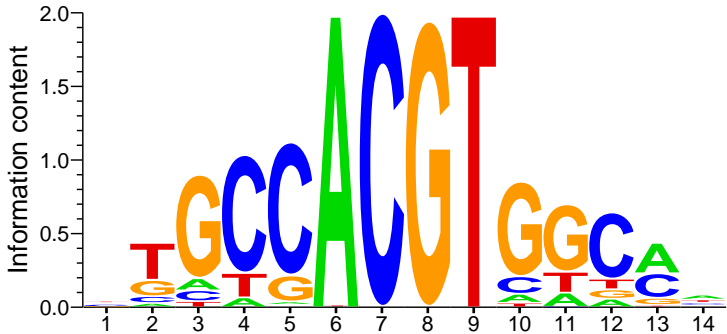

Supplement: Supplementary file 6 — Dataset EV1 [file MSB-13-910-s006.zip › Yang_Orenstein_DatasetEV1/bZIP_CREB3L1_TGGGTA30NTGT_CACGTG_14_5.pwm.pdf]

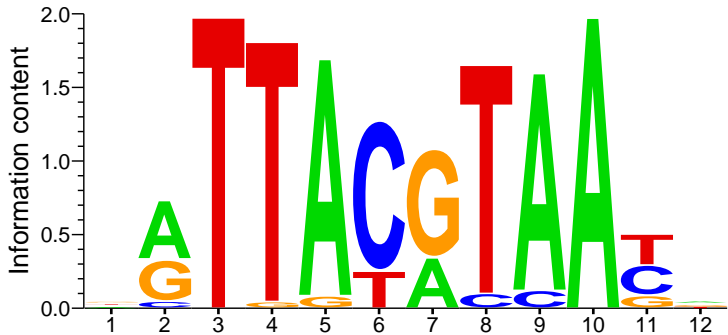

Supplement: Supplementary file 6 — Dataset EV1 [file MSB-13-910-s006.zip › Yang_Orenstein_DatasetEV1/bZIP_Dbp_TAAATG20NCG_TACGTA_12_3.pwm.pdf]

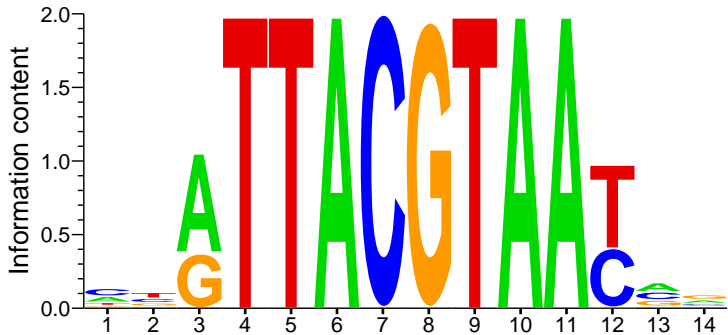

Supplement: Supplementary file 6 — Dataset EV1 [file MSB-13-910-s006.zip › Yang_Orenstein_DatasetEV1/bZIP_DBP_TAACTT20NCG_TACGTA_14_3.pwm.pdf]

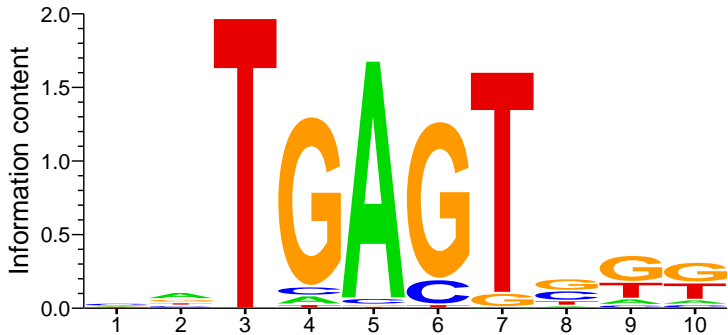

Supplement: Supplementary file 6 — Dataset EV1 [file MSB-13-910-s006.zip › Yang_Orenstein_DatasetEV1/bZIP_JDP2_TGTTCA20NGA_TGASTC_10_3.pwm.pdf]

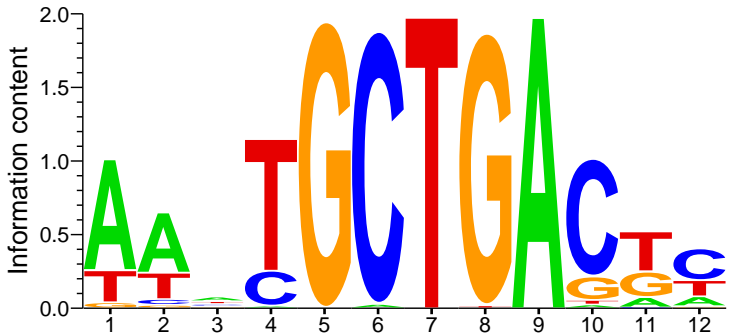

Supplement: Supplementary file 6 — Dataset EV1 [file MSB-13-910-s006.zip › Yang_Orenstein_DatasetEV1/bZIP_Mafb_TCGTTA20NTTT_YGCTGA_12_4.pwm.pdf]

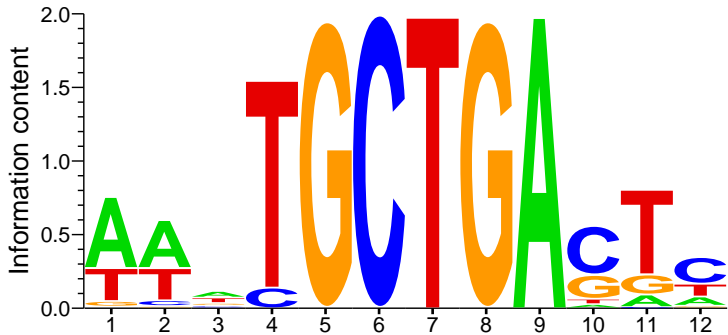

Supplement: Supplementary file 6 — Dataset EV1 [file MSB-13-910-s006.zip › Yang_Orenstein_DatasetEV1/bZIP_MAFK_TGCCTG30NTCC_TGCTGA_12_4.pwm.pdf]

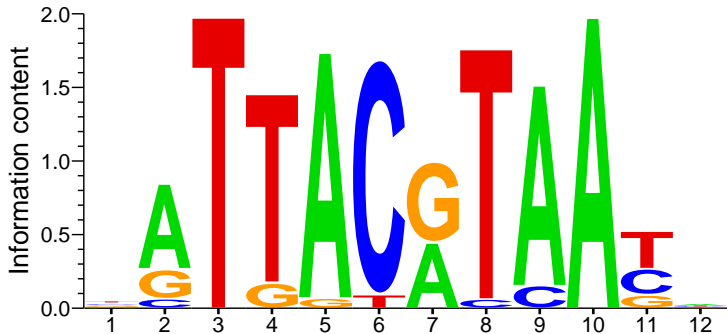

Supplement: Supplementary file 6 — Dataset EV1 [file MSB-13-910-s006.zip › Yang_Orenstein_DatasetEV1/bZIP_NFIL3_TAGACG20NCG_TACRTA_12_3.pwm.pdf]

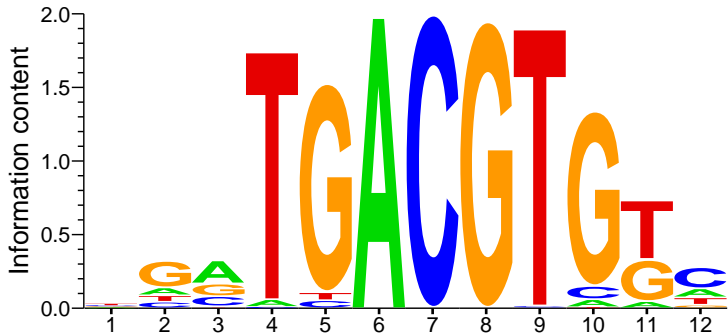

Supplement: Supplementary file 6 — Dataset EV1 [file MSB-13-910-s006.zip › Yang_Orenstein_DatasetEV1/bZIP_XBP1_TGTTCA20NGA_TGACGT_12_4.pwm.pdf]

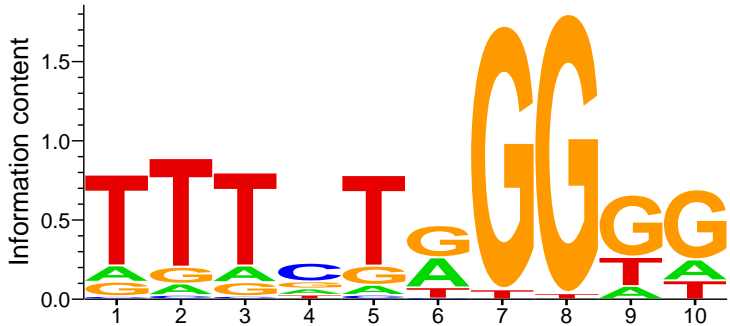

Supplement: Supplementary file 6 — Dataset EV1 [file MSB-13-910-s006.zip › Yang_Orenstein_DatasetEV1/C2H2_BCL6B_TGCGGG20NGA_TTTCTAGGAA_10_3.pwm.pdf]

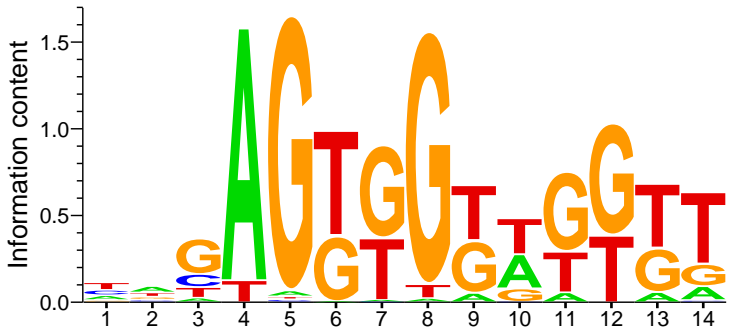

Supplement: Supplementary file 6 — Dataset EV1 [file MSB-13-910-s006.zip › Yang_Orenstein_DatasetEV1/C2H2_Egr1_TGCGCC20NCG_GAGKGGGCGK_14_3.pwm.pdf]

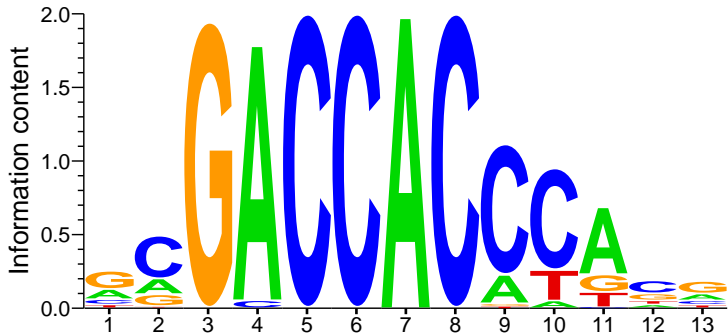

Supplement: Supplementary file 6 — Dataset EV1 [file MSB-13-910-s006.zip › Yang_Orenstein_DatasetEV1/C2H2_GLI2_TAAGTA40NTGA_GACCACNCT_13_3.pwm.pdf]

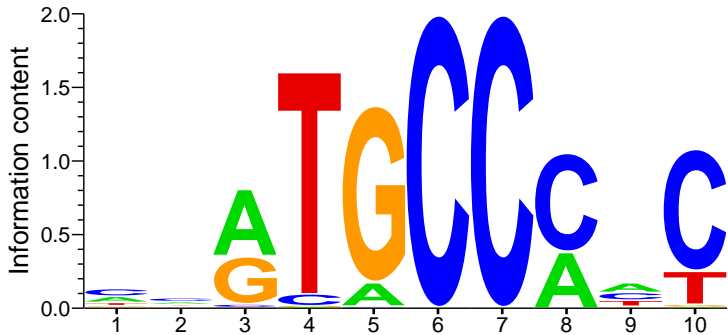

Supplement: Supplementary file 6 — Dataset EV1 [file MSB-13-910-s006.zip › Yang_Orenstein_DatasetEV1/C2H2_Hic1_TCATCA20NCTA_RTGCCM_10_3.pwm.pdf]

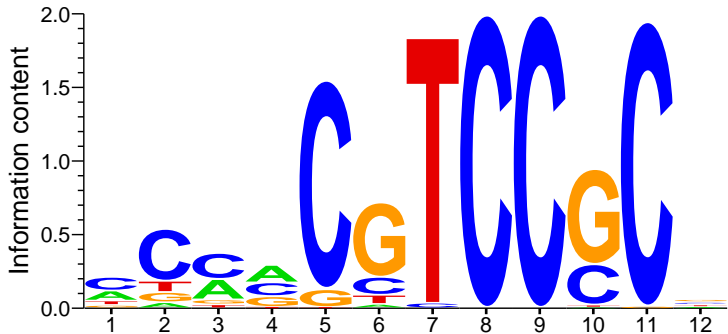

Supplement: Supplementary file 6 — Dataset EV1 [file MSB-13-910-s006.zip › Yang_Orenstein_DatasetEV1/C2H2_HINFP1_TCAAGG20NGAA_NNNSGTCCGC_12_3.pwm.pdf]

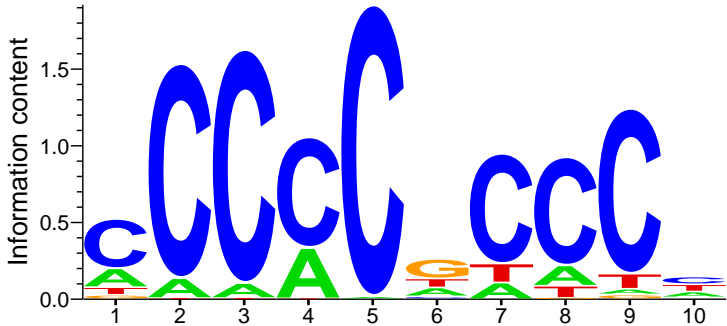

Supplement: Supplementary file 6 — Dataset EV1 [file MSB-13-910-s006.zip › Yang_Orenstein_DatasetEV1/C2H2_KLF16_TTGATT20NTA_CCMCGCCC_10_4.pwm.pdf]

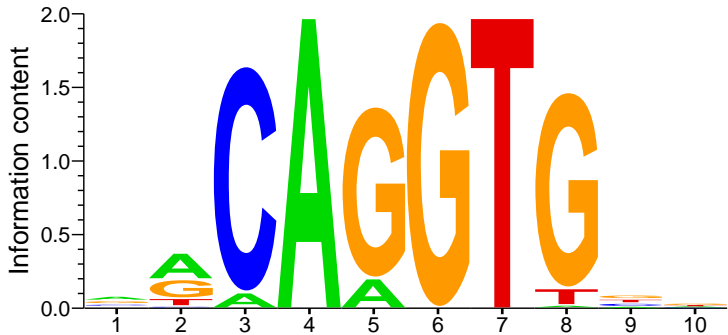

Supplement: Supplementary file 6 — Dataset EV1 [file MSB-13-910-s006.zip › Yang_Orenstein_DatasetEV1/C2H2_SNAI2_TCGTTA20NGA_CAGGTG_10_5.pwm.pdf]

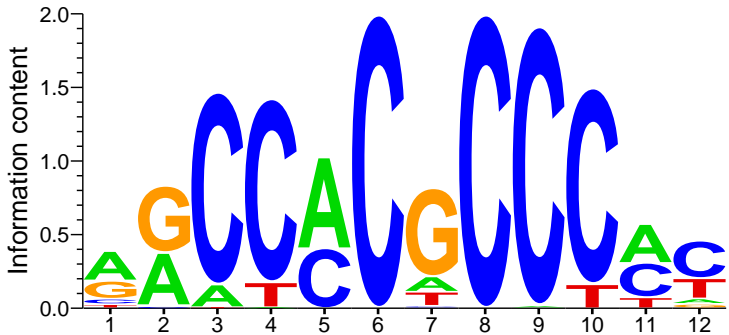

Supplement: Supplementary file 6 — Dataset EV1 [file MSB-13-910-s006.zip › Yang_Orenstein_DatasetEV1/C2H2_SP1_TCTATG20NGA_GCCMCGCCCM_12_3.pwm.pdf]

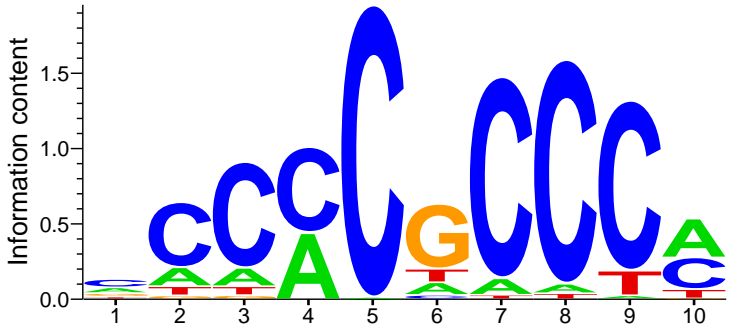

Supplement: Supplementary file 6 — Dataset EV1 [file MSB-13-910-s006.zip › Yang_Orenstein_DatasetEV1/C2H2_SP3_TCGGCC20NGA_GCCMCGCCCM_10_3.pwm.pdf]

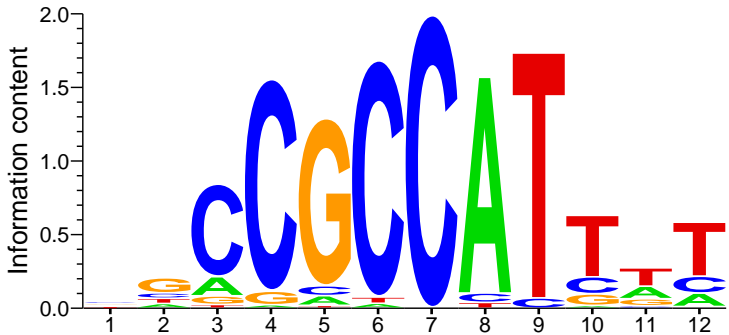

Supplement: Supplementary file 6 — Dataset EV1 [file MSB-13-910-s006.zip › Yang_Orenstein_DatasetEV1/C2H2_YY1_TCCGGC20NCG_CCGCCATT_12_4.pwm.pdf]

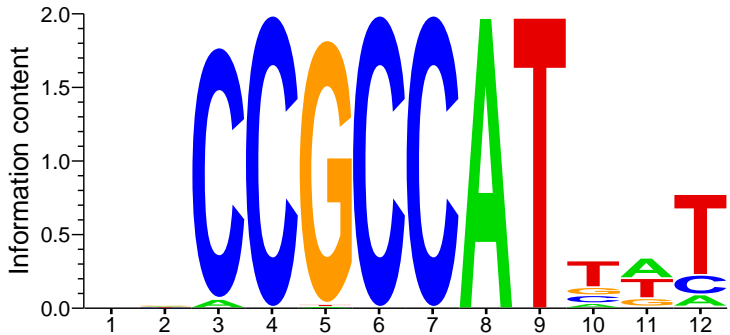

Supplement: Supplementary file 6 — Dataset EV1 [file MSB-13-910-s006.zip › Yang_Orenstein_DatasetEV1/C2H2_YY2_TGAGCA20NGA_CCGCCATN_12_4.pwm.pdf]

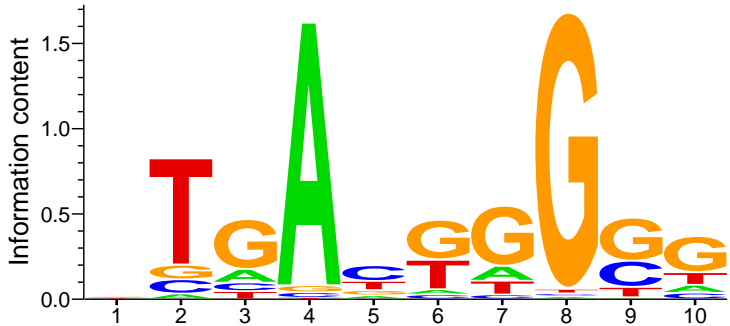

Supplement: Supplementary file 6 — Dataset EV1 [file MSB-13-910-s006.zip › Yang_Orenstein_DatasetEV1/C2H2_ZBTB49_TCTAAT20NGA_TGACNNGC_10_3.pwm.pdf]

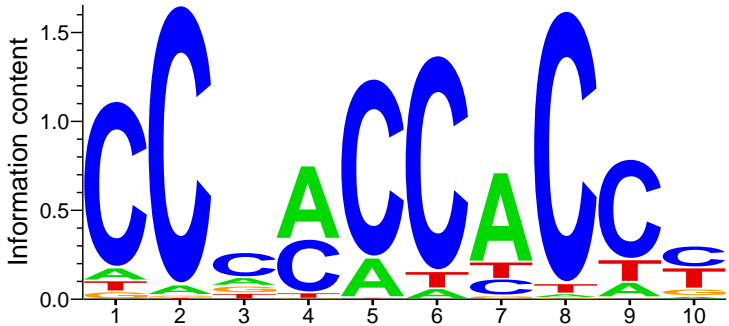

Supplement: Supplementary file 6 — Dataset EV1 [file MSB-13-910-s006.zip › Yang_Orenstein_DatasetEV1/C2H2_ZBTB7A_TGAATA20NGA_CGACCACC_10_3.pwm.pdf]

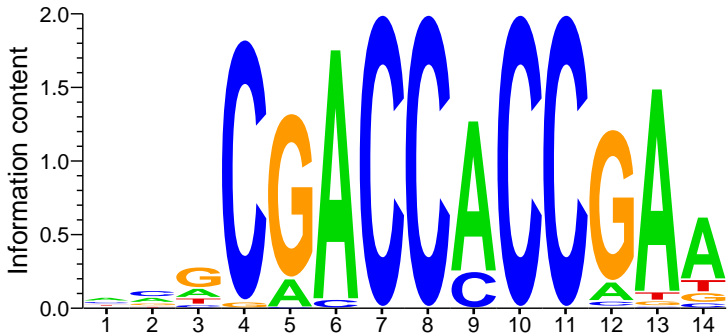

Supplement: Supplementary file 6 — Dataset EV1 [file MSB-13-910-s006.zip › Yang_Orenstein_DatasetEV1/C2H2_ZBTB7B_TAGCCT20NCG_CGACCACC_14_3.pwm.pdf]

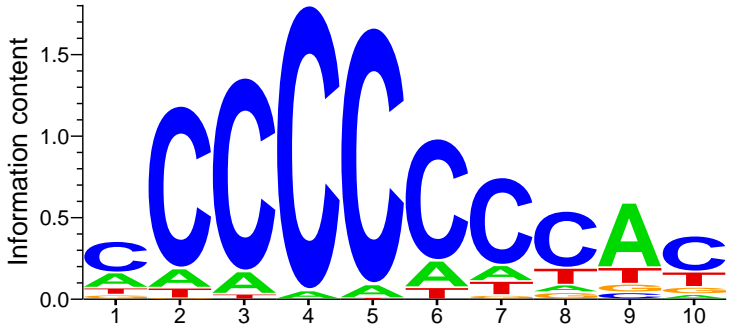

Supplement: Supplementary file 6 — Dataset EV1 [file MSB-13-910-s006.zip › Yang_Orenstein_DatasetEV1/C2H2_Zfp740_TCAACC20NCG_NCCCCCCCAC_10_4.pwm.pdf]

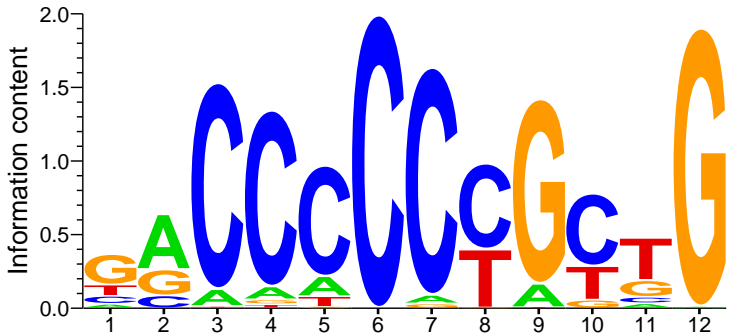

Supplement: Supplementary file 6 — Dataset EV1 [file MSB-13-910-s006.zip › Yang_Orenstein_DatasetEV1/C2H2_ZIC1_TCGACT20NCAT_GACCMCCYRMTG_12_3.pwm.pdf]

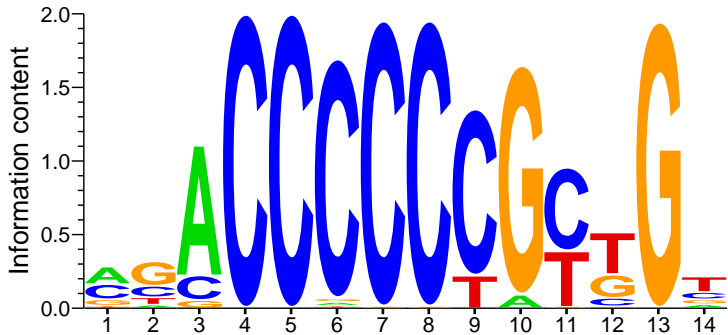

Supplement: Supplementary file 6 — Dataset EV1 [file MSB-13-910-s006.zip › Yang_Orenstein_DatasetEV1/C2H2_ZIC4_TGAGGC20NGA_GACCCCCYGYTG_14_3.pwm.pdf]

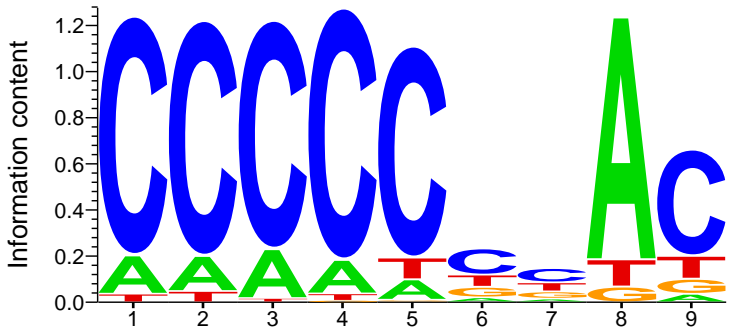

Supplement: Supplementary file 6 — Dataset EV1 [file MSB-13-910-s006.zip › Yang_Orenstein_DatasetEV1/C2H2_ZNF740_TGTGAG20NGA_CCCCCCCAC_9_3.pwm.pdf]

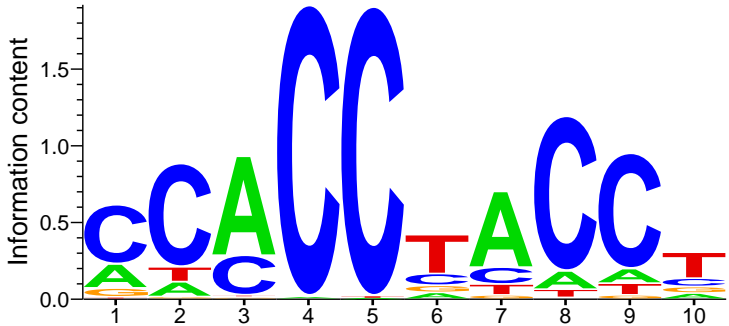

Supplement: Supplementary file 6 — Dataset EV1 [file MSB-13-910-s006.zip › Yang_Orenstein_DatasetEV1/C2H2_ZNF784_TGCGGG20NGA_GTACCTACCT_10_3.pwm.pdf]

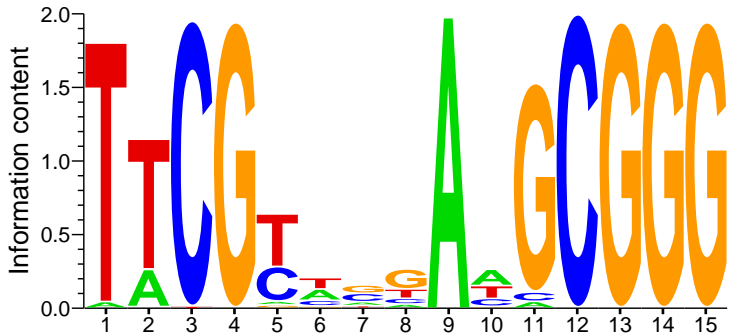

Supplement: Supplementary file 6 — Dataset EV1 [file MSB-13-910-s006.zip › Yang_Orenstein_DatasetEV1/CENPB_CENPB_TGCTGT20NGTC_TTCGYNNNANGCGGG_15_3.pwm.pdf]

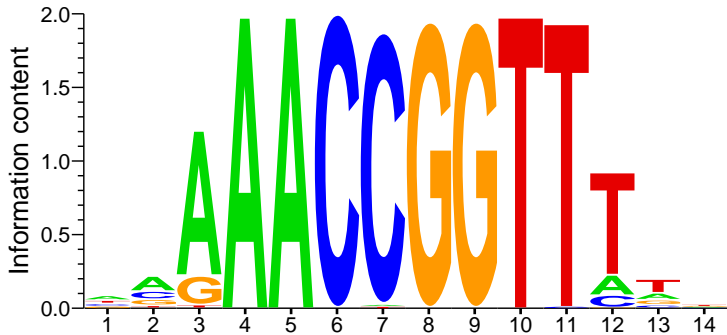

Supplement: Supplementary file 6 — Dataset EV1 [file MSB-13-910-s006.zip › Yang_Orenstein_DatasetEV1/CP2_GRHL1_TGGACA20NCAT_AACCGGTT_14_4.pwm.pdf]

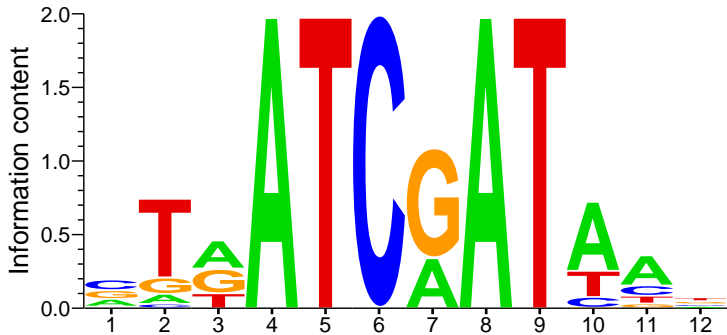

Supplement: Supplementary file 6 — Dataset EV1 [file MSB-13-910-s006.zip › Yang_Orenstein_DatasetEV1/CUT_CUX1_TAGCAG40NACT_ATCRAT_12_5.pwm.pdf]

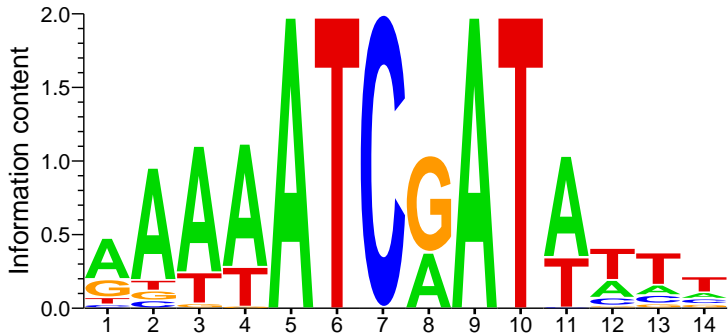

Supplement: Supplementary file 6 — Dataset EV1 [file MSB-13-910-s006.zip › Yang_Orenstein_DatasetEV1/CUT_ONECUT1_TAGCTC20NTCT_ATCRAT_14_3.pwm.pdf]

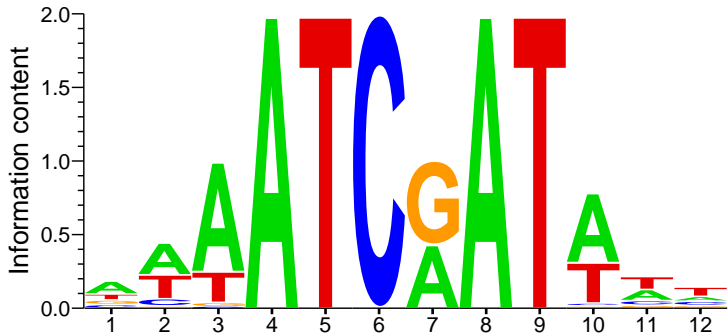

Supplement: Supplementary file 6 — Dataset EV1 [file MSB-13-910-s006.zip › Yang_Orenstein_DatasetEV1/CUT_ONECUT3_TAGAAC20NAAT_ATCRAT_12_3.pwm.pdf]

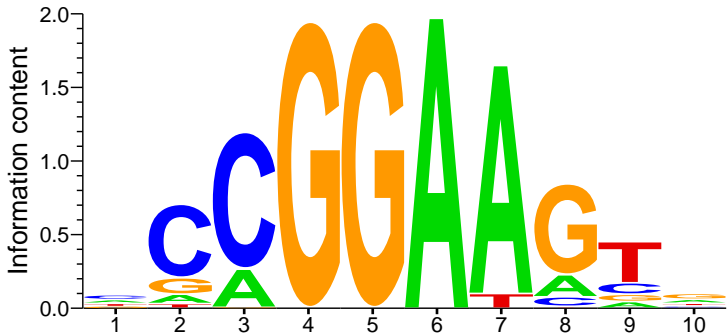

Supplement: Supplementary file 6 — Dataset EV1 [file MSB-13-910-s006.zip › Yang_Orenstein_DatasetEV1/ETS_ELF1_TGGGTA20NGA_GGAA_10_3.pwm.pdf]

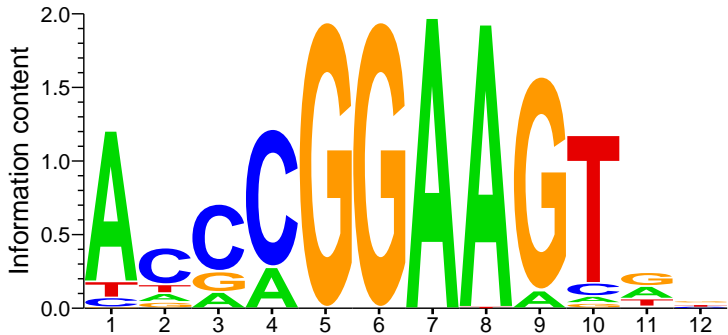

Supplement: Supplementary file 6 — Dataset EV1 [file MSB-13-910-s006.zip › Yang_Orenstein_DatasetEV1/ETS_Elf5_TATTGT20NCG_GGAA_12_4.pwm.pdf]

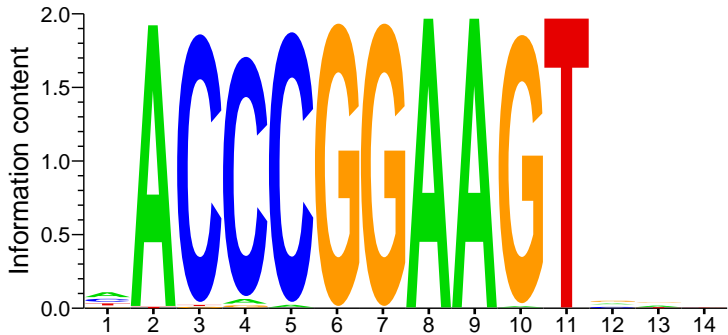

Supplement: Supplementary file 6 — Dataset EV1 [file MSB-13-910-s006.zip › Yang_Orenstein_DatasetEV1/ETS_ELF5_TGCCGC20NCG_GGAA_14_3.pwm.pdf]

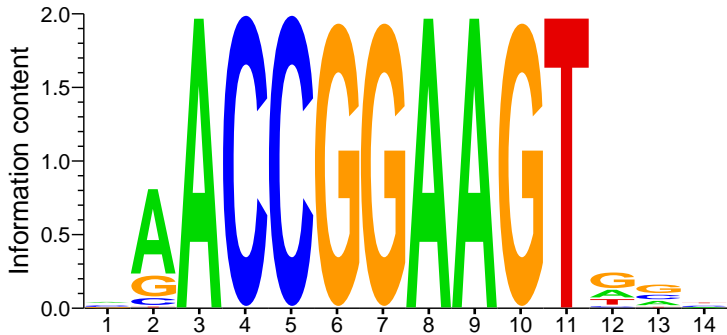

Supplement: Supplementary file 6 — Dataset EV1 [file MSB-13-910-s006.zip › Yang_Orenstein_DatasetEV1/ETS_ELK1_TCGGAA20NAGT_GGAA_14_3.pwm.pdf]

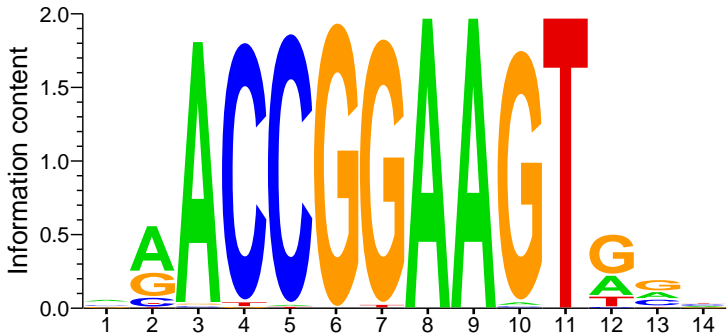

Supplement: Supplementary file 6 — Dataset EV1 [file MSB-13-910-s006.zip › Yang_Orenstein_DatasetEV1/ETS_ELK3_TCGGGG20NGGT_GGAA_14_3.pwm.pdf]

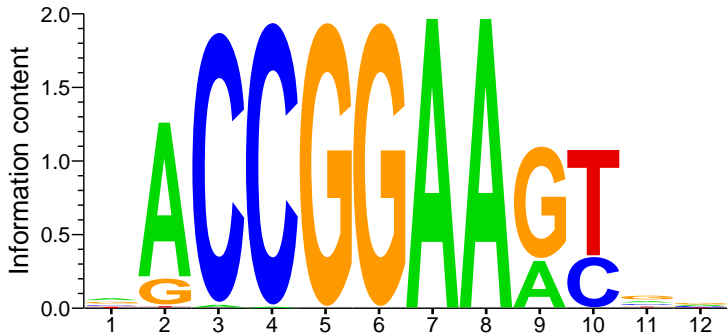

Supplement: Supplementary file 6 — Dataset EV1 [file MSB-13-910-s006.zip › Yang_Orenstein_DatasetEV1/ETS_ELK4_TAGGGC40NGGT_GGAA_12_3.pwm.pdf]

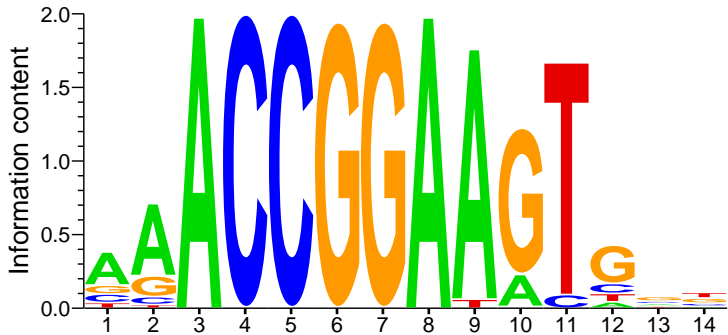

Supplement: Supplementary file 6 — Dataset EV1 [file MSB-13-910-s006.zip › Yang_Orenstein_DatasetEV1/ETS_ERG_TGCAAG20NAAC_GGAW_14_4.pwm.pdf]

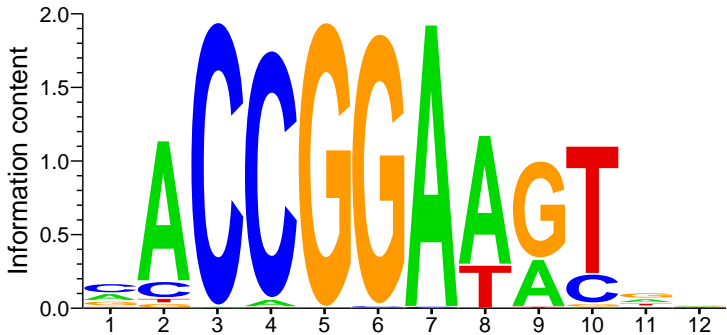

Supplement: Supplementary file 6 — Dataset EV1 [file MSB-13-910-s006.zip › Yang_Orenstein_DatasetEV1/ETS_ETS1_TGTAAA20NGA_GGAW_12_3.pwm.pdf]

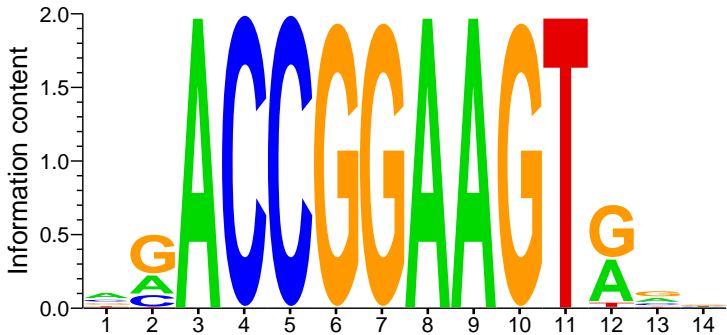

Supplement: Supplementary file 6 — Dataset EV1 [file MSB-13-910-s006.zip › Yang_Orenstein_DatasetEV1/ETS_ETV1_TCGTTA20NTTT_GGAA_14_4.pwm.pdf]

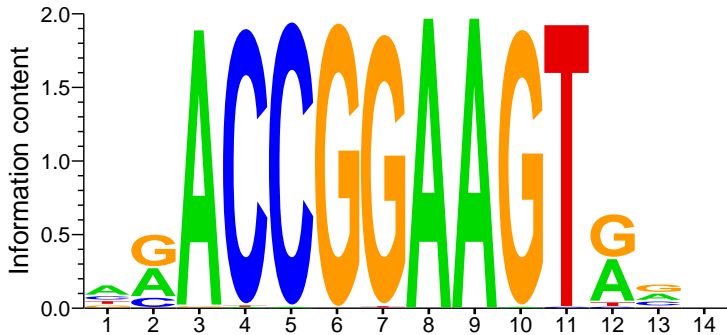

Supplement: Supplementary file 6 — Dataset EV1 [file MSB-13-910-s006.zip › Yang_Orenstein_DatasetEV1/ETS_ETV4_TCTATG20NTAG_GGAA_14_4.pwm.pdf]

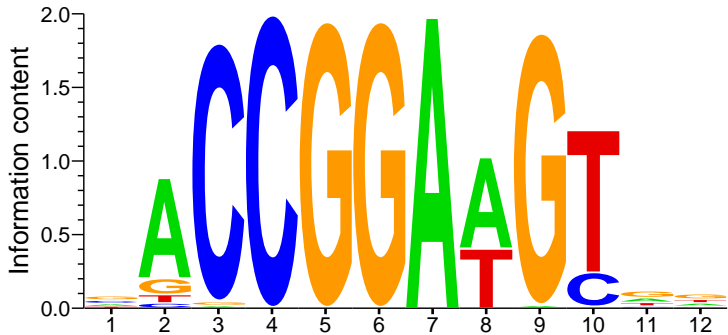

Supplement: Supplementary file 6 — Dataset EV1 [file MSB-13-910-s006.zip › Yang_Orenstein_DatasetEV1/ETS_ETV5_TGCACT20NGA_GGAW_12_3.pwm.pdf]

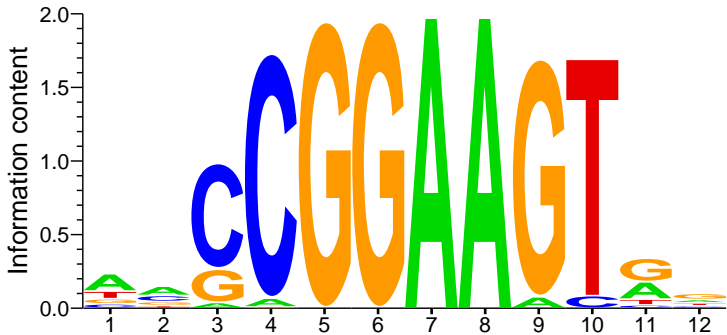

Supplement: Supplementary file 6 — Dataset EV1 [file MSB-13-910-s006.zip › Yang_Orenstein_DatasetEV1/ETS_ETV6_TGAGTG20NGA_GGAA_12_3.pwm.pdf]

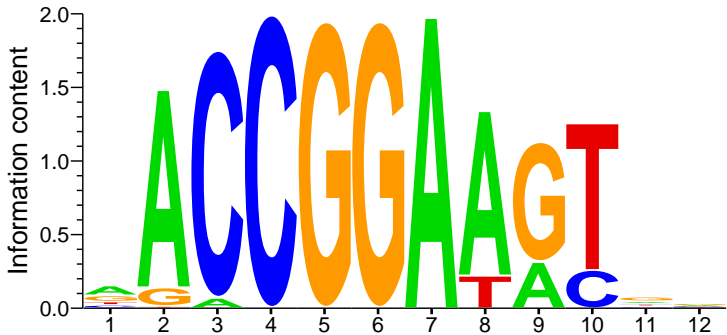

Supplement: Supplementary file 6 — Dataset EV1 [file MSB-13-910-s006.zip › Yang_Orenstein_DatasetEV1/ETS_FEV_TGATCC20NCTA_GGAA_12_3.pwm.pdf]

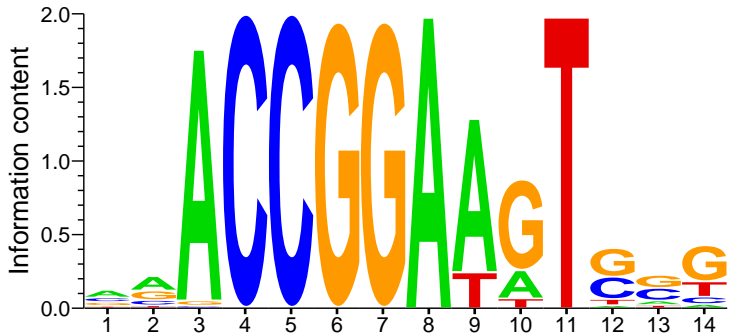

Supplement: Supplementary file 6 — Dataset EV1 [file MSB-13-910-s006.zip › Yang_Orenstein_DatasetEV1/ETS_FLI1_TCTAAT20NCG_GGAW_14_4.pwm.pdf]

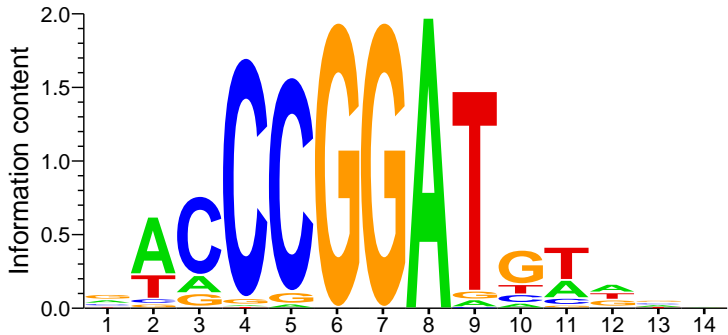

Supplement: Supplementary file 6 — Dataset EV1 [file MSB-13-910-s006.zip › Yang_Orenstein_DatasetEV1/ETS_SPDEF_TCATTG20NCG_GGAT_14_4.pwm.pdf]

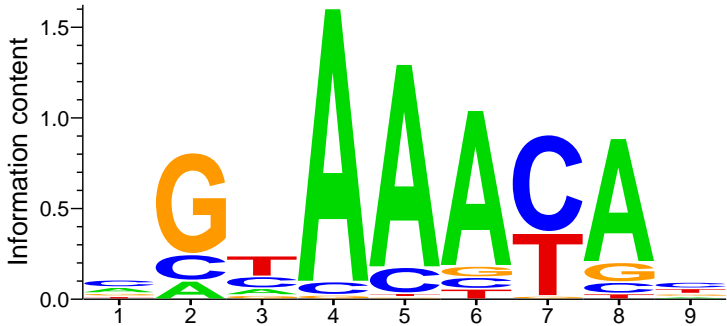

Supplement: Supplementary file 6 — Dataset EV1 [file MSB-13-910-s006.zip › Yang_Orenstein_DatasetEV1/forkhead_FOXB1_TCTGGT20NGGG_GTAAAYA_9_3.pwm.pdf]

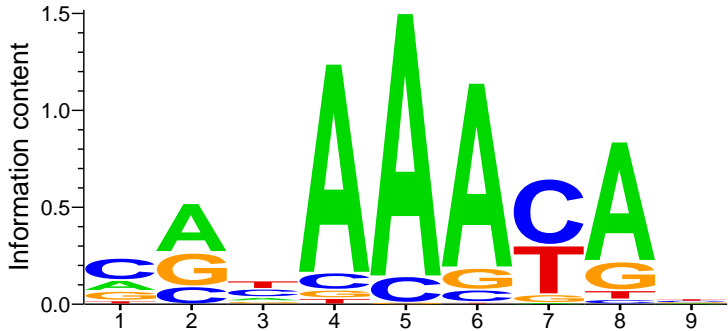

Supplement: Supplementary file 6 — Dataset EV1 [file MSB-13-910-s006.zip › Yang_Orenstein_DatasetEV1/forkhead_Foxc1_CCCCT14N_RTAAAYA_9_3.pwm.pdf]

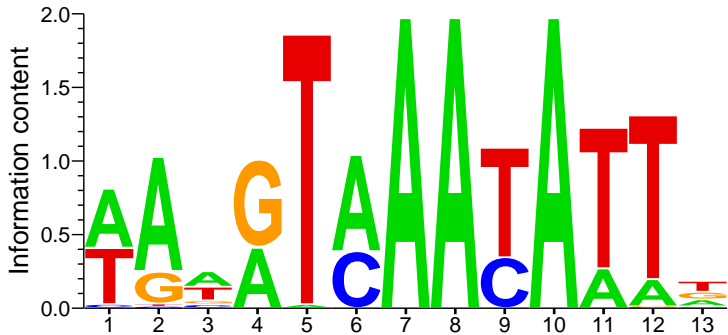

Supplement: Supplementary file 6 — Dataset EV1 [file MSB-13-910-s006.zip › Yang_Orenstein_DatasetEV1/forkhead_FOXC1_TGAGGC30NGGA_RTMAAYA_13_3.pwm.pdf]

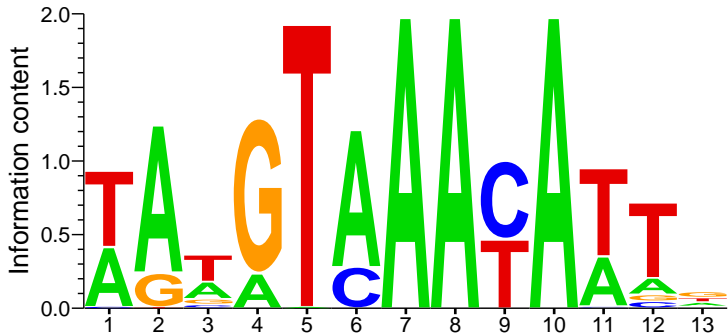

Supplement: Supplementary file 6 — Dataset EV1 [file MSB-13-910-s006.zip › Yang_Orenstein_DatasetEV1/forkhead_FOXC2_TGAGTG30NTGA_RTAAAYA_13_4.pwm.pdf]

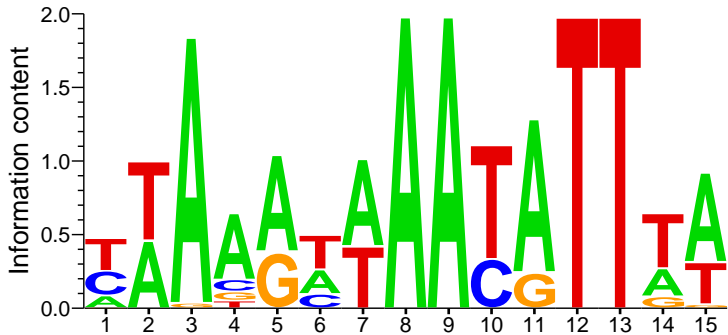

Supplement: Supplementary file 6 — Dataset EV1 [file MSB-13-910-s006.zip › Yang_Orenstein_DatasetEV1/forkhead_FOXD2_TGCTCG20NCTC_RNWAAYR_15_3.pwm.pdf]

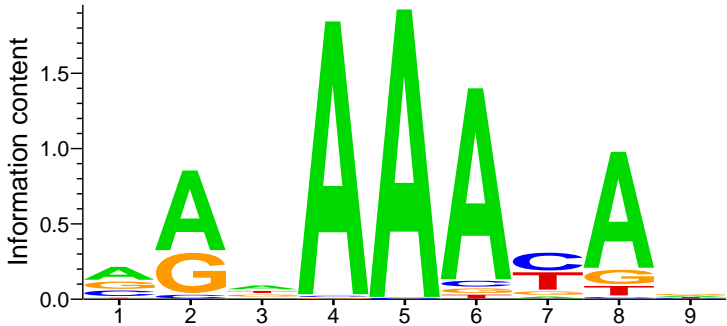

Supplement: Supplementary file 6 — Dataset EV1 [file MSB-13-910-s006.zip › Yang_Orenstein_DatasetEV1/forkhead_Foxg1_TATCTG20NTCG_RTAAAYA_9_3.pwm.pdf]

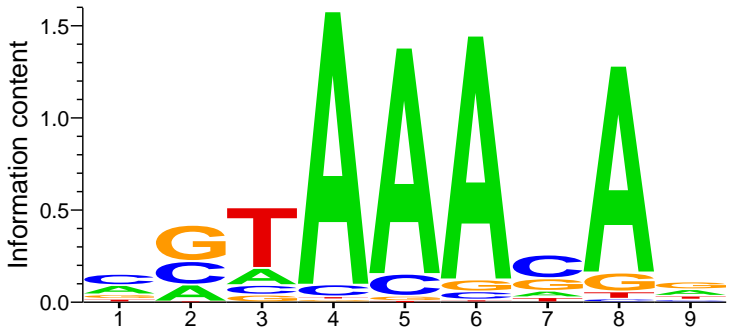

Supplement: Supplementary file 6 — Dataset EV1 [file MSB-13-910-s006.zip › Yang_Orenstein_DatasetEV1/forkhead_FOXI1_TCGGAA20NGA_GTAAACA_9_4.pwm.pdf]

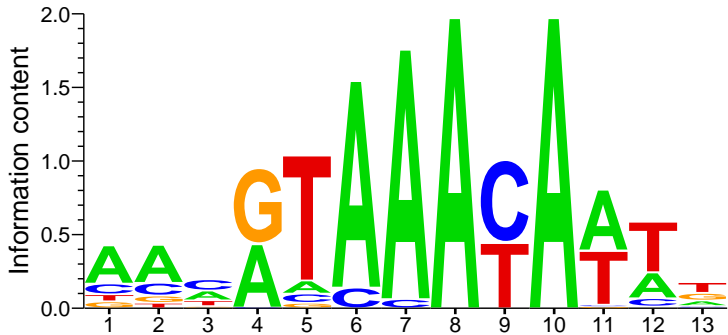

Supplement: Supplementary file 6 — Dataset EV1 [file MSB-13-910-s006.zip › Yang_Orenstein_DatasetEV1/forkhead_FOXJ2_TGACGA20NGA_RTAAACA_13_3.pwm.pdf]

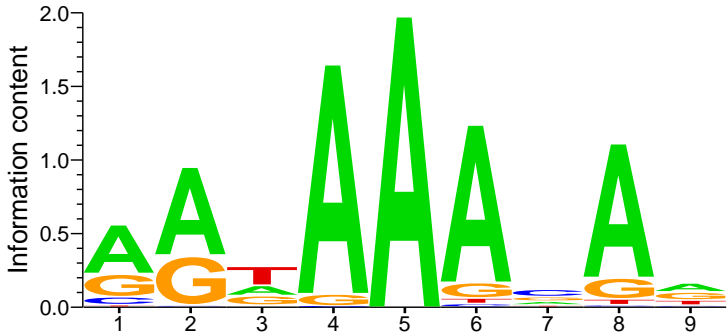

Supplement: Supplementary file 6 — Dataset EV1 [file MSB-13-910-s006.zip › Yang_Orenstein_DatasetEV1/forkhead_FOXJ3_TAGTGG20NGTT_RTAAACA_9_3.pwm.pdf]

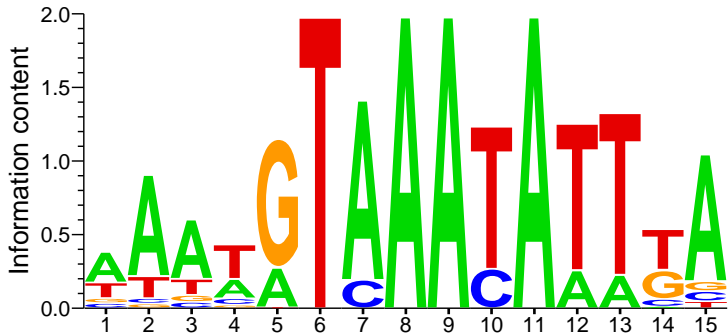

Supplement: Supplementary file 6 — Dataset EV1 [file MSB-13-910-s006.zip › Yang_Orenstein_DatasetEV1/forkhead_FOXL1_TGCTCG20NCTC_RTAAAYA_15_3.pwm.pdf]

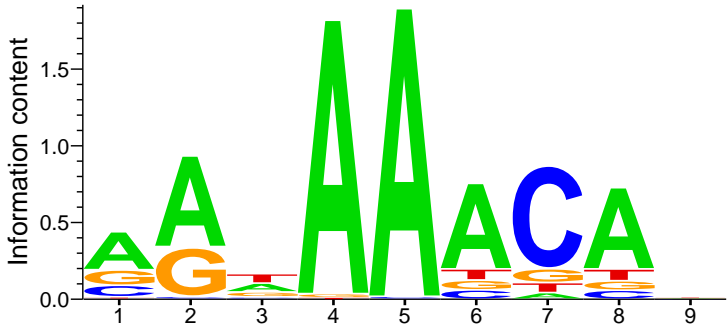

Supplement: Supplementary file 6 — Dataset EV1 [file MSB-13-910-s006.zip › Yang_Orenstein_DatasetEV1/forkhead_FOXP3_TCAATT20NTAA_RTAAACA_9_3.pwm.pdf]

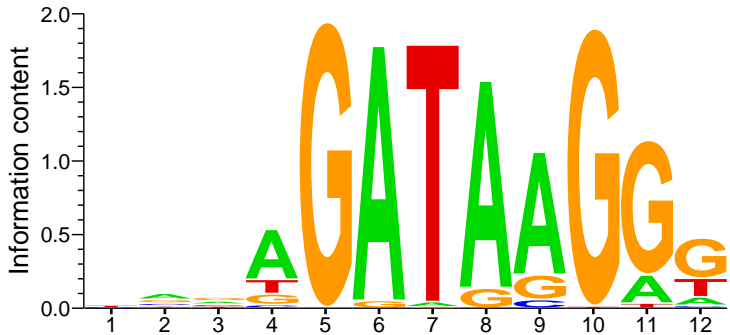

Supplement: Supplementary file 6 — Dataset EV1 [file MSB-13-910-s006.zip › Yang_Orenstein_DatasetEV1/GATA_GATA3_TCATGC20NCG_GATA_12_3.pwm.pdf]

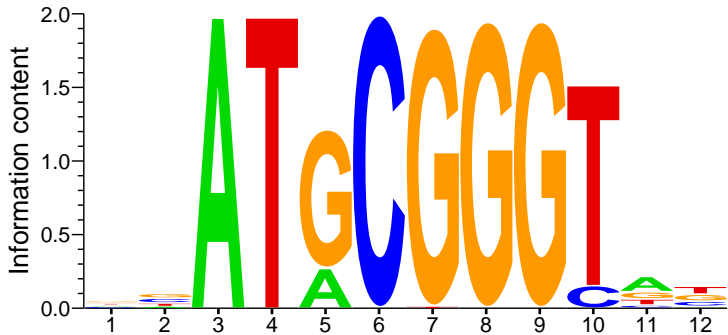

Supplement: Supplementary file 6 — Dataset EV1 [file MSB-13-910-s006.zip › Yang_Orenstein_DatasetEV1/GCM_GCM1_TGAGCA20NCGA_ATGCGGGT_12_3.pwm.pdf]

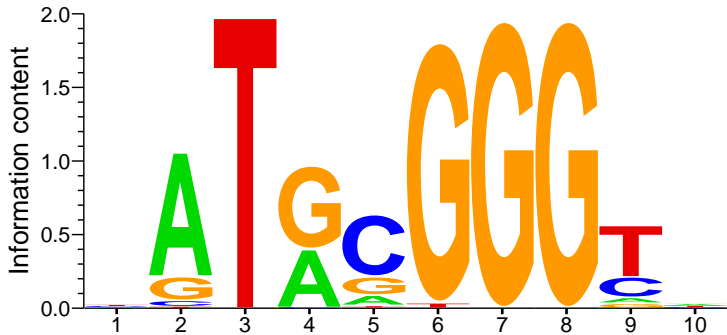

Supplement: Supplementary file 6 — Dataset EV1 [file MSB-13-910-s006.zip › Yang_Orenstein_DatasetEV1/GCM_GCM2_TGACTC20NTCA_ATGCGGGT_10_4.pwm.pdf]

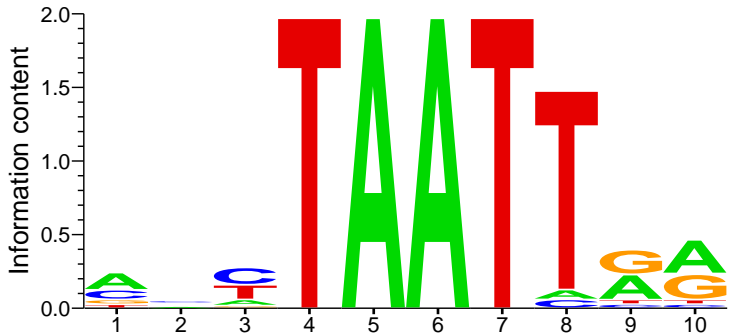

Supplement: Supplementary file 6 — Dataset EV1 [file MSB-13-910-s006.zip › Yang_Orenstein_DatasetEV1/homeodomain_Alx1_TAAAGC20NCG_TAAT_10_4.pwm.pdf]

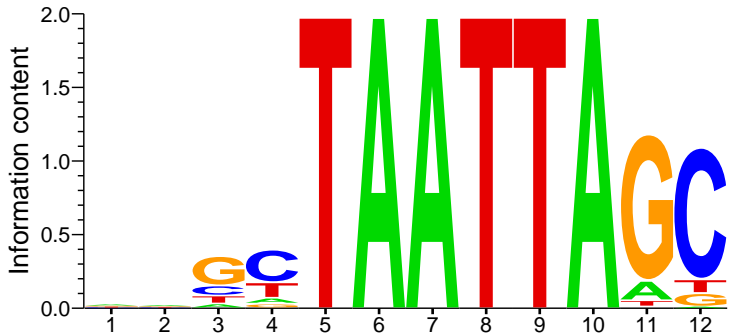

Supplement: Supplementary file 6 — Dataset EV1 [file MSB-13-910-s006.zip › Yang_Orenstein_DatasetEV1/homeodomain_ALX3_TGTAAA20NAAG_TAAT_12_4.pwm.pdf]

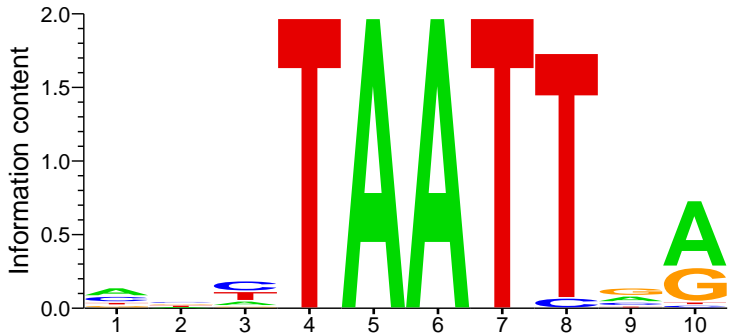

Supplement: Supplementary file 6 — Dataset EV1 [file MSB-13-910-s006.zip › Yang_Orenstein_DatasetEV1/homeodomain_Alx4_TGGTAG20NCG_TAAT_10_4.pwm.pdf]

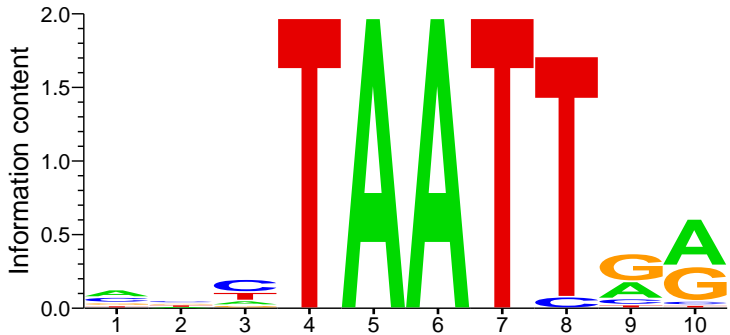

Supplement: Supplementary file 6 — Dataset EV1 [file MSB-13-910-s006.zip › Yang_Orenstein_DatasetEV1/homeodomain_ALX4_TGTGTC20NGA_TAAT_10_4.pwm.pdf]

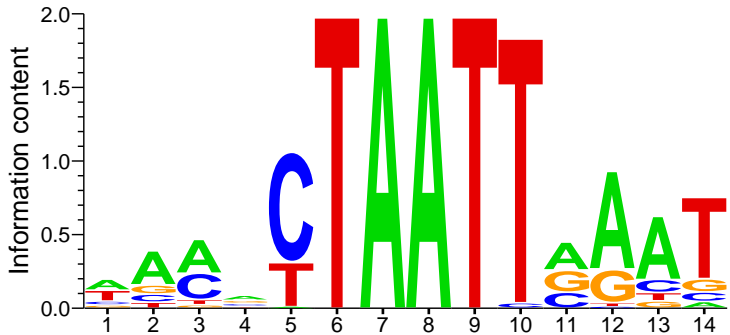

Supplement: Supplementary file 6 — Dataset EV1 [file MSB-13-910-s006.zip › Yang_Orenstein_DatasetEV1/homeodomain_Arx_TCGCAT20NACT_TAAT_14_3.pwm.pdf]

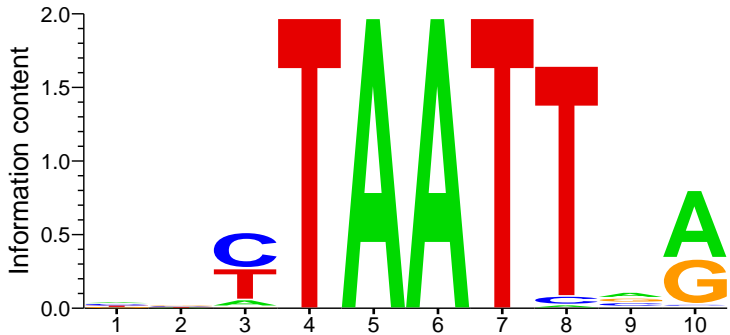

Supplement: Supplementary file 6 — Dataset EV1 [file MSB-13-910-s006.zip › Yang_Orenstein_DatasetEV1/homeodomain_ARX_TGCGTT20NTGC_TAAT_10_3.pwm.pdf]

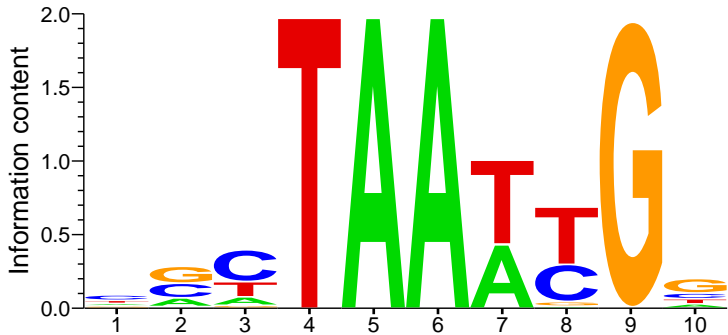

Supplement: Supplementary file 6 — Dataset EV1 [file MSB-13-910-s006.zip › Yang_Orenstein_DatasetEV1/homeodomain_Barhl1_TCAGTC20NCG_TAAW_10_4.pwm.pdf]

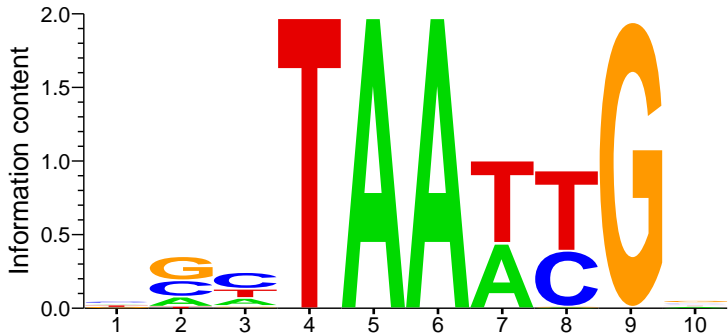

Supplement: Supplementary file 6 — Dataset EV1 [file MSB-13-910-s006.zip › Yang_Orenstein_DatasetEV1/homeodomain_BARHL2_TATTGT20NGTG_TAAW_10_3.pwm.pdf]

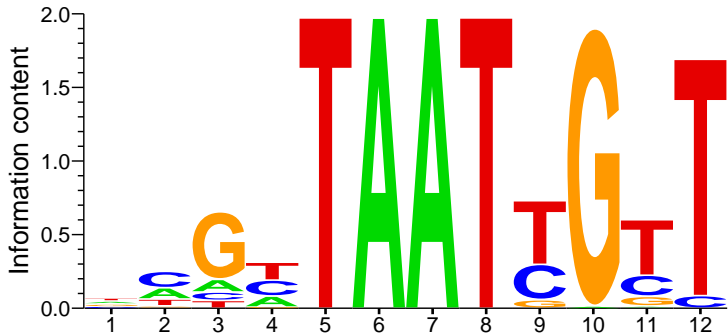

Supplement: Supplementary file 6 — Dataset EV1 [file MSB-13-910-s006.zip › Yang_Orenstein_DatasetEV1/homeodomain_BARX1_TATTCG20NCTG_TAAT_12_3.pwm.pdf]

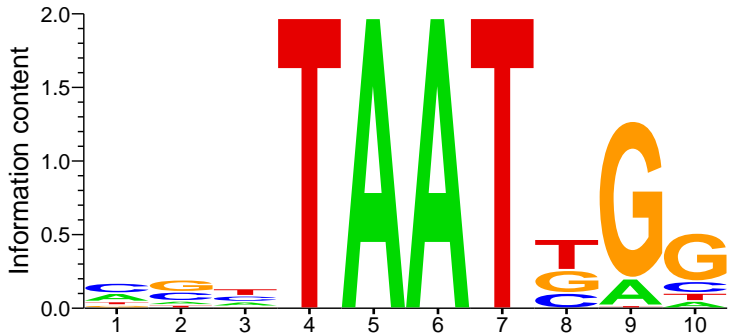

Supplement: Supplementary file 6 — Dataset EV1 [file MSB-13-910-s006.zip › Yang_Orenstein_DatasetEV1/homeodomain_BSX_TATGAA20NCG_TAAT_10_4.pwm.pdf]

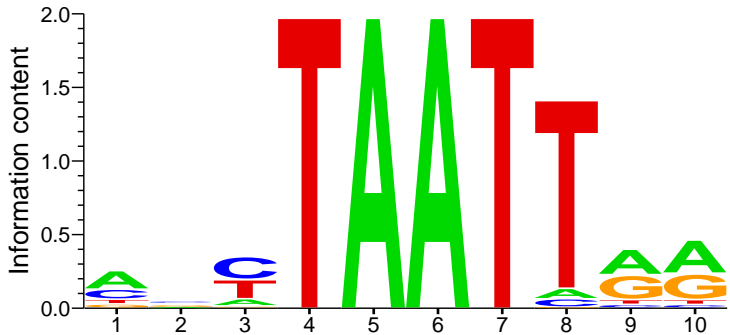

Supplement: Supplementary file 6 — Dataset EV1 [file MSB-13-910-s006.zip › Yang_Orenstein_DatasetEV1/homeodomain_CART1_TGCGCC20NGA_TAAT_10_4.pwm.pdf]

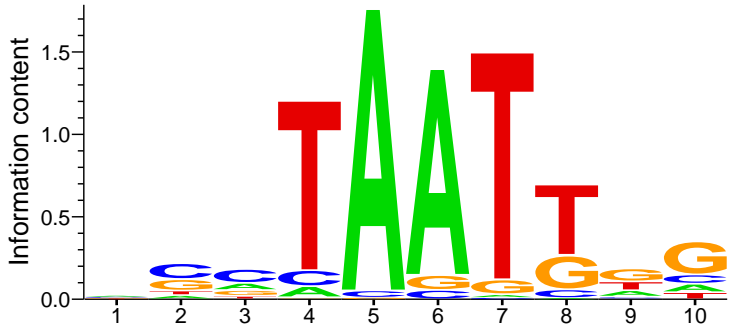

Supplement: Supplementary file 6 — Dataset EV1 [file MSB-13-910-s006.zip › Yang_Orenstein_DatasetEV1/homeodomain_DLX1_TCTTGA20NGA_TAAT_10_3.pwm.pdf]

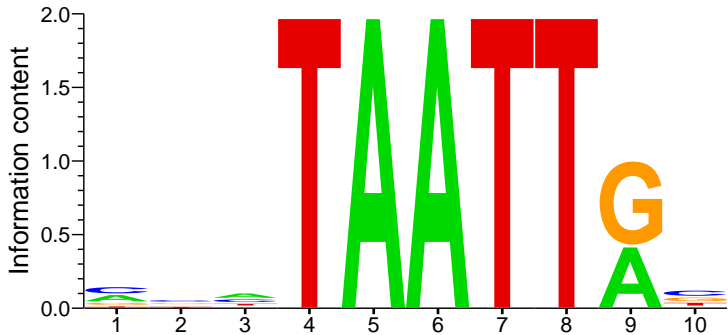

Supplement: Supplementary file 6 — Dataset EV1 [file MSB-13-910-s006.zip › Yang_Orenstein_DatasetEV1/homeodomain_Dlx1_TGAGAT20NCG_TAAT_10_5.pwm.pdf]

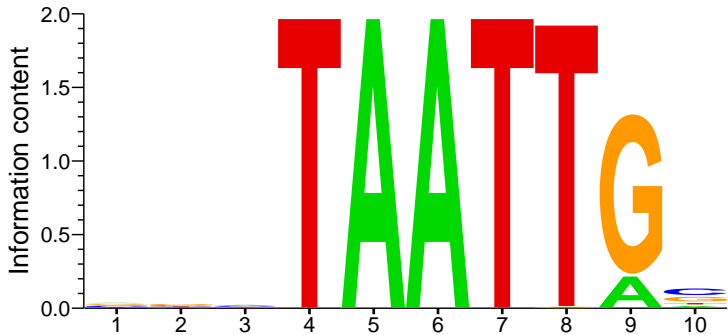

Supplement: Supplementary file 6 — Dataset EV1 [file MSB-13-910-s006.zip › Yang_Orenstein_DatasetEV1/homeodomain_Dlx2_TCGCCA20NCCT_TAAT_10_4.pwm.pdf]

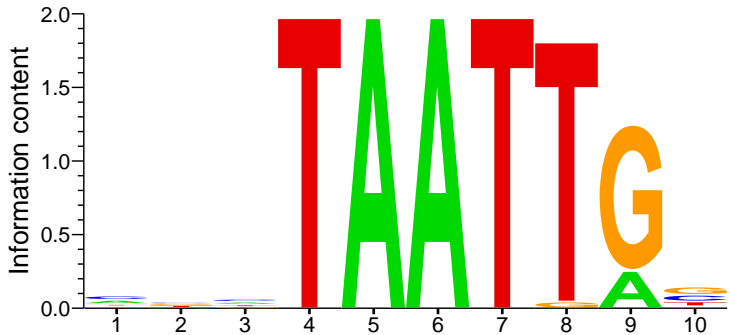

Supplement: Supplementary file 6 — Dataset EV1 [file MSB-13-910-s006.zip › Yang_Orenstein_DatasetEV1/homeodomain_DLX2_TTCATG20NTA_TAAT_10_4.pwm.pdf]

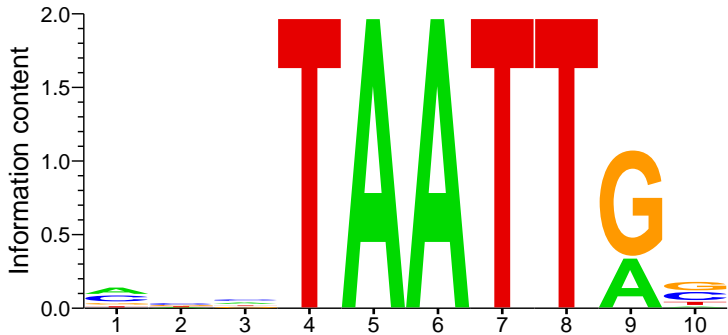

Supplement: Supplementary file 6 — Dataset EV1 [file MSB-13-910-s006.zip › Yang_Orenstein_DatasetEV1/homeodomain_DLX3_TATGTT20NCG_TAAT_10_4.pwm.pdf]

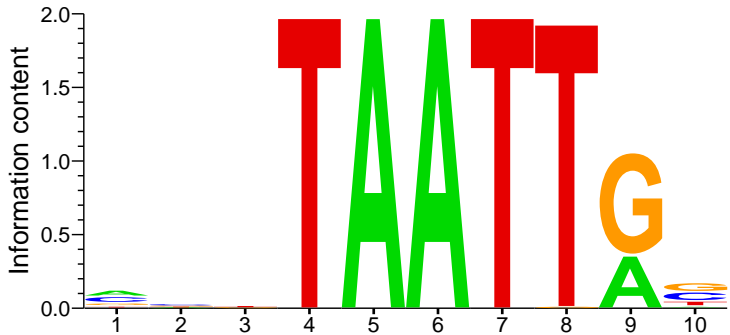

Supplement: Supplementary file 6 — Dataset EV1 [file MSB-13-910-s006.zip › Yang_Orenstein_DatasetEV1/homeodomain_DLX4_TCTTCT20NGA_TAAT_10_4.pwm.pdf]

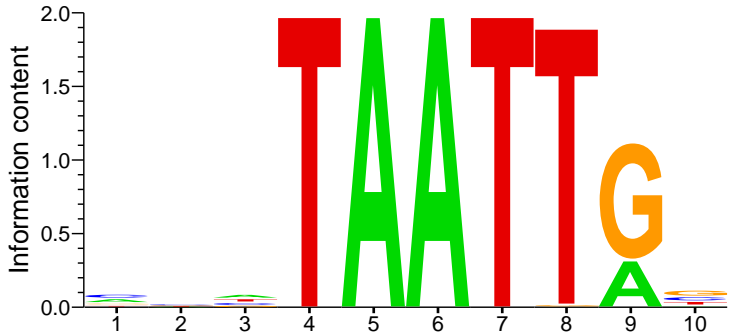

Supplement: Supplementary file 6 — Dataset EV1 [file MSB-13-910-s006.zip › Yang_Orenstein_DatasetEV1/homeodomain_DLX6_TGCGGG20NGA_TAAT_10_3.pwm.pdf]

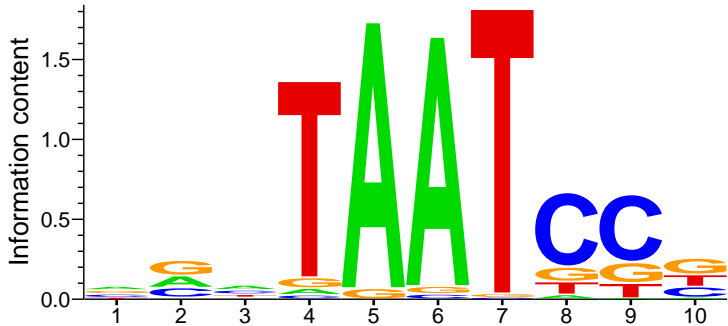

Supplement: Supplementary file 6 — Dataset EV1 [file MSB-13-910-s006.zip › Yang_Orenstein_DatasetEV1/homeodomain_DMBX1_TAACCC20NCCA_TAAT_10_3.pwm.pdf]

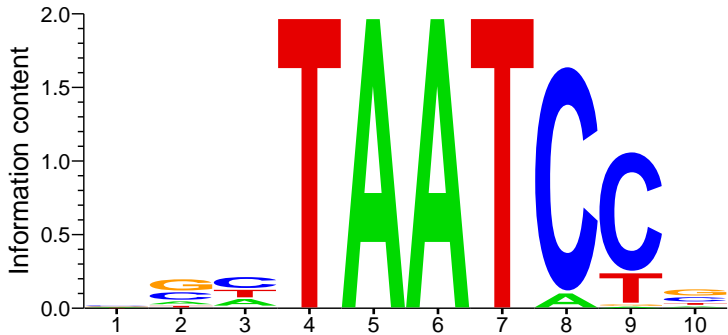

Supplement: Supplementary file 6 — Dataset EV1 [file MSB-13-910-s006.zip › Yang_Orenstein_DatasetEV1/homeodomain_DPRX_TCGAAG30NAAT_TAAT_10_6.pwm.pdf]

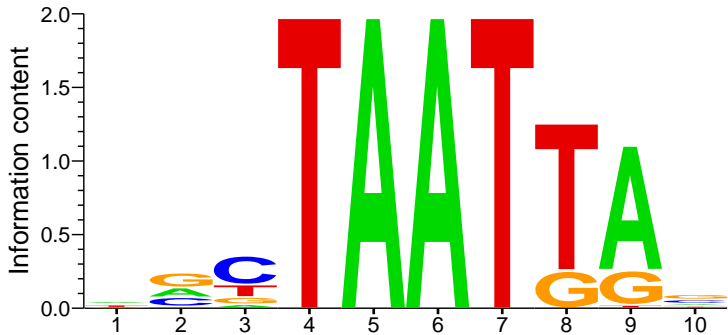

Supplement: Supplementary file 6 — Dataset EV1 [file MSB-13-910-s006.zip › Yang_Orenstein_DatasetEV1/homeodomain_EMX2_TGGTTC30NTTT_TAAT_10_5.pwm.pdf]

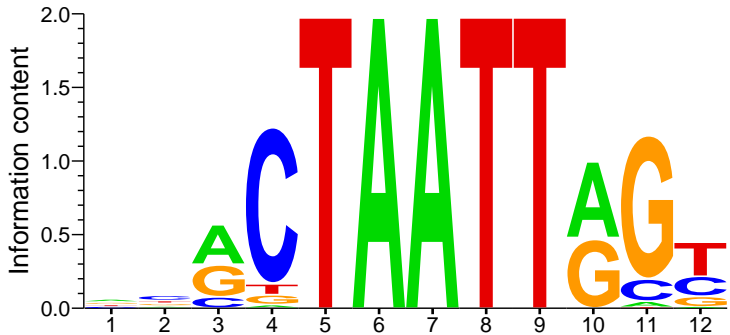

Supplement: Supplementary file 6 — Dataset EV1 [file MSB-13-910-s006.zip › Yang_Orenstein_DatasetEV1/homeodomain_EN1_TGACCT20NCCA_TAAT_12_4.pwm.pdf]

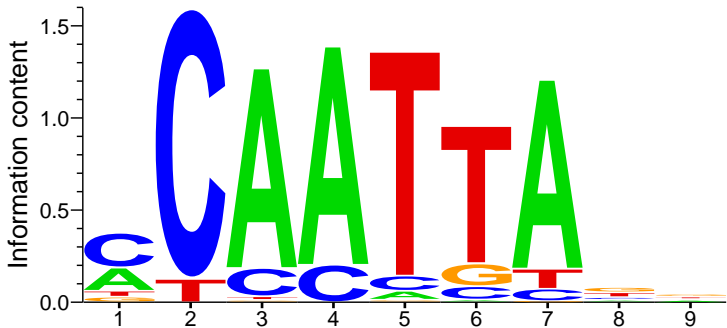

Supplement: Supplementary file 6 — Dataset EV1 [file MSB-13-910-s006.zip › Yang_Orenstein_DatasetEV1/homeodomain_En2_TCAGCT20NCG_YAATTAN_9_4.pwm.pdf]

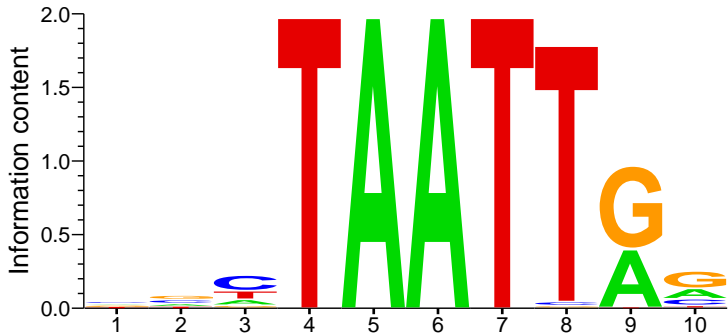

Supplement: Supplementary file 6 — Dataset EV1 [file MSB-13-910-s006.zip › Yang_Orenstein_DatasetEV1/homeodomain_ESX1_TCTTCT20NGA_TAAT_10_4.pwm.pdf]
