# Supplementary material for: Transcription factor family‐specific DNA shape readout revealed by quantitative specificity models
Source: Mol Syst Biol. 2017 Feb 6;13(2):910. doi: 10.15252/msb.20167238 (PMC5327724; doi:10.15252/msb.20167238)
Supplement: Supplementary file 6 — Dataset EV1 [file MSB-13-910-s006.zip › Yang_Orenstein_DatasetEV1/POU_POU3F4_TACTGC20NGTC_WAAT_10_4.pwm.pdf]

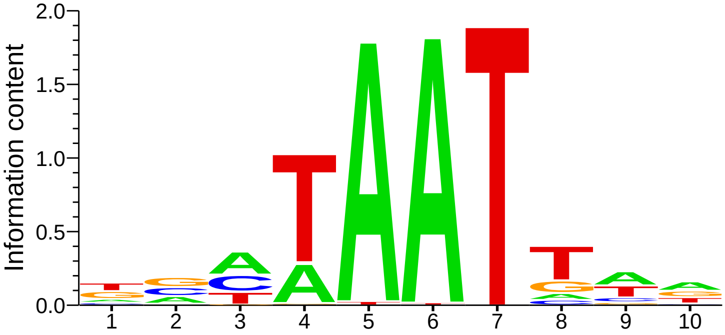
Information content
2.0
1.5
1.0
0.5
0.0
1
2
3
4
5
6
7
8
9
10
