# Supplementary material for: Transcription factor family‐specific DNA shape readout revealed by quantitative specificity models
Source: Mol Syst Biol. 2017 Feb 6;13(2):910. doi: 10.15252/msb.20167238 (PMC5327724; doi:10.15252/msb.20167238)
Supplement: Supplementary file 7 — Dataset EV2 [file MSB-13-910-s007.zip › Yang_Orenstein_DatasetEV2/POU_POU3F1_TAGCGA20NGCT_WAAT_16_4_shape.pwm.pdf]

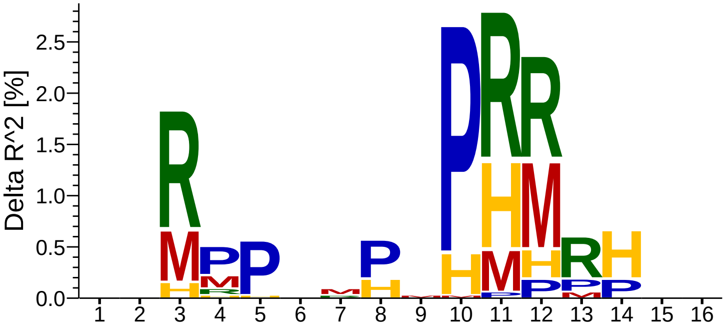
Delta R^2 [%]
0.0
0.5
1.0
1.5
2.0
2.5
1 2 3 4 5 6 7 8 9 10 11 12 13 14 15 16
