# Supplementary material for: Transcription factor family‐specific DNA shape readout revealed by quantitative specificity models
Source: Mol Syst Biol. 2017 Feb 6;13(2):910. doi: 10.15252/msb.20167238 (PMC5327724; doi:10.15252/msb.20167238)
Supplement: Supplementary file 7 — Dataset EV2 [file MSB-13-910-s007.zip › Yang_Orenstein_DatasetEV2/TBX_MGA_TAGGGC20NGGT_AGGTGTGA_12_3_shape.pwm.pdf]

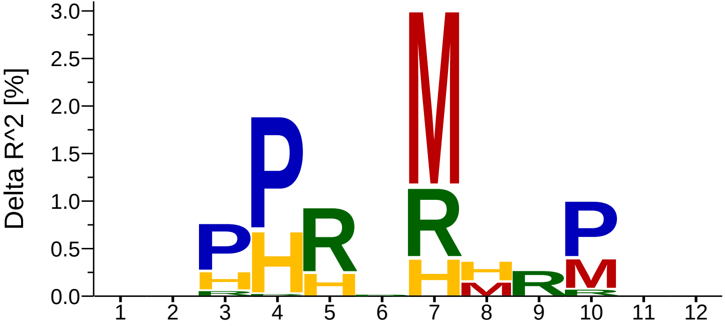

Delta R^2 [%]
3.0
2.5
2.0
1.5
1.0
0.5
0.0
1
2
3
4
5
6
7
8
9
10
11
12
