# Supplementary material for: Transcription factor family‐specific DNA shape readout revealed by quantitative specificity models
Source: Mol Syst Biol. 2017 Feb 6;13(2):910. doi: 10.15252/msb.20167238 (PMC5327724; doi:10.15252/msb.20167238)
Supplement: Supplementary file 7 — Dataset EV2 [file MSB-13-910-s007.zip › Yang_Orenstein_DatasetEV2/TBX_TBX19_TACTAT40NATC_ANGTGYGA_12_4_shape.pwm.pdf]

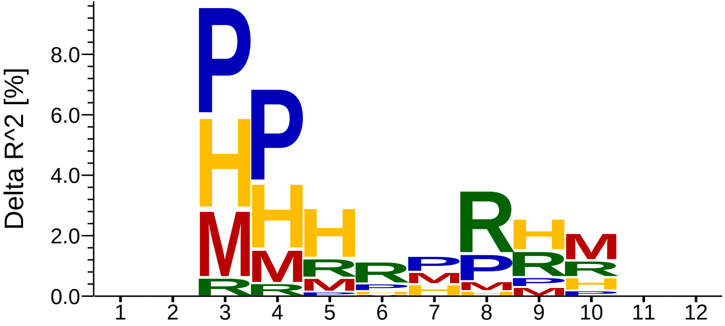
Delta R^2 [%]
0.0
2.0
4.0
6.0
8.0
1
2
3
4
5
6
7
8
9
10
11
12
